# Brønsted acid-promoted thiazole synthesis under metal-free conditions using sulfur powder as the sulfur source†

**DOI:** 10.1039/c9ra09656f

**Published:** 2020-01-23

**Authors:** Penghui Ni, Jing Tan, Rong Li, Huawen Huang, Feng Zhang, Guo-Jun Deng

**Affiliations:** Key Laboratory for Green Organic Synthesis and Application of Hunan Province, Key Laboratory of Environmentally Friendly Chemistry and Application of Ministry of Education, College of Chemistry, Xiangtan University Xiangtan 411105 China gjdeng@xtu.edu.cn; College of Science, Hunan Agricultural University Changsha 410128 China zhangf@iccas.ac.cn

## Abstract

A Brønsted acid-promoted sulfuration/annulation reaction for the one-pot synthesis of bis-substituted thiazoles from benzylamines, acetophenones, and sulfur powder has been developed. One C–N bond and multi C–S bonds were selectively formed in one pot. The choice of the Brønsted acid was the key to the high efficiency of this transformation under metal-free conditions.

## Abstract 2

A Brønsted acid-promoted protocol for the synthesis of 2,4-disubstituted thiazoles from benzylamines, acetophenones, and sulfur powder under metal-free conditions is described.

## Graphical abstract

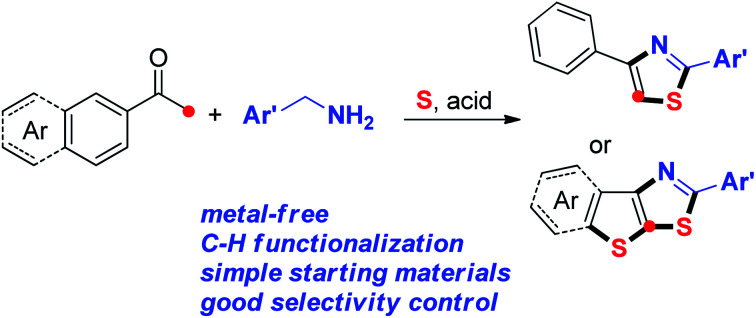

At least 50% of the biologically active compounds have a heterocyclic skeleton.^1^ Among these, the thiazole ring is an important five-membered aromatic heterocycle with nitrogen and sulfur atoms, and the unique structure has led to many applications in different pharmaceuticals and biological processes.^2^ For example (Fig. 1), antimicrobial (Abafungin),^3^ antihypertension (Arotinolol),^4^ anti-inflammatory (Meloxicam),^5^ and immunomodulatory (Fanetizole)^6^ drugs are prevalent among the drugs based on thiazole that have reached the marketplace.^7^

**Fig. 1:**
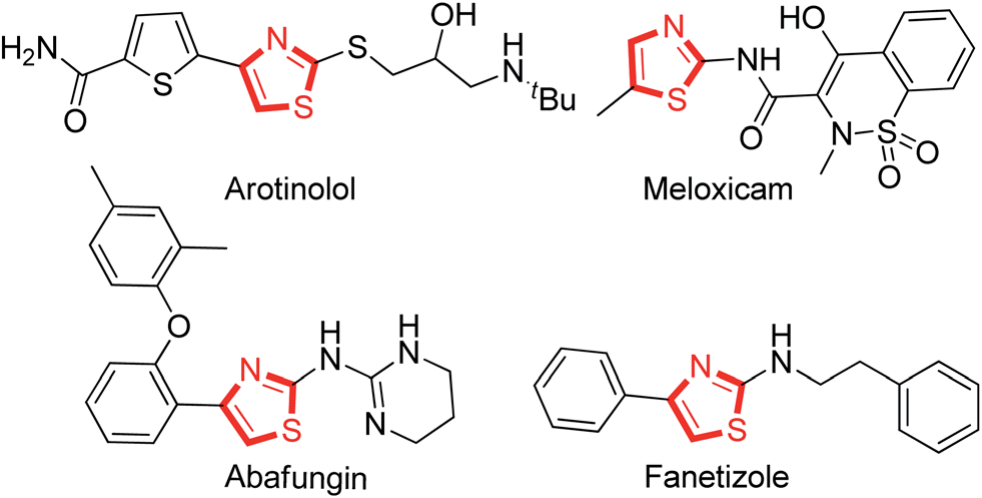
Selected commercial drugs based on thiazole.

In view of this, great efforts have been invested in the development of novel synthetic protocols to facilitate the construction of thiazole derivatives. The typical procedure for the synthesis of thiazoles involves the reaction of α-haloketones with thioureas/thioamides using catalysts such as cyclodextrin,^8^ iodine,^9^ silica chloride,^10^ baker's yeast,^11^ and others^12^ (Scheme 1a). Besides, Wu^13^ and co-workers developed a catalyst-free protocol for the construction of polysubstituted thiazoles from α-haloketones and thioureas/thioamides. Togo^14^ reported the efficient synthesis of thiazoles *via* a base-promoted 1*H*-1-(1′-alkynyl)-5-methyl-1,2,3-benziodoxathiole 3,3-dioxide reaction with thioamides. Recently, Kshirsagar^15^ and co-workers developed NIS-mediated intermolecular cyclization of styrenes and thioamides using water as the solvent. On the other hand, the transition metal-catalyzed direct coupling of pre-existing thiazole compounds provides an alternative approach (Scheme 1b).^16^ Very recently, Jiao^17^ and co-workers developed a novel Cu-catalyzed aerobic oxidative approach to obtain thiazoles using elemental sulfur as the sulfur source *via* a multiple Csp^3^–H bond cleavage strategy (Scheme 1c). In spite of synthetic efficiency, these methods suffer from limitations with respect to special substrates and transition-metal catalysts. Therefore, the development of efficient methods for the synthesis of thiazoles from simple and readily available substrates under metal-free conditions is highly desirable. It is well-known that the sulfur element is cheap, stable, and easy to handle and thus, it is an ideal sulfur source for C–S bond construction.^18^ In our continuing efforts on using elemental sulfur for the synthesis of sulfur-containing heterocycles under simple conditions,^19^ we describe a three-component strategy for thiazole formation from readily available acetophenones, benzylamines, and sulfur powder under metal-free conditions (Scheme 1d).

**Scheme 1:**
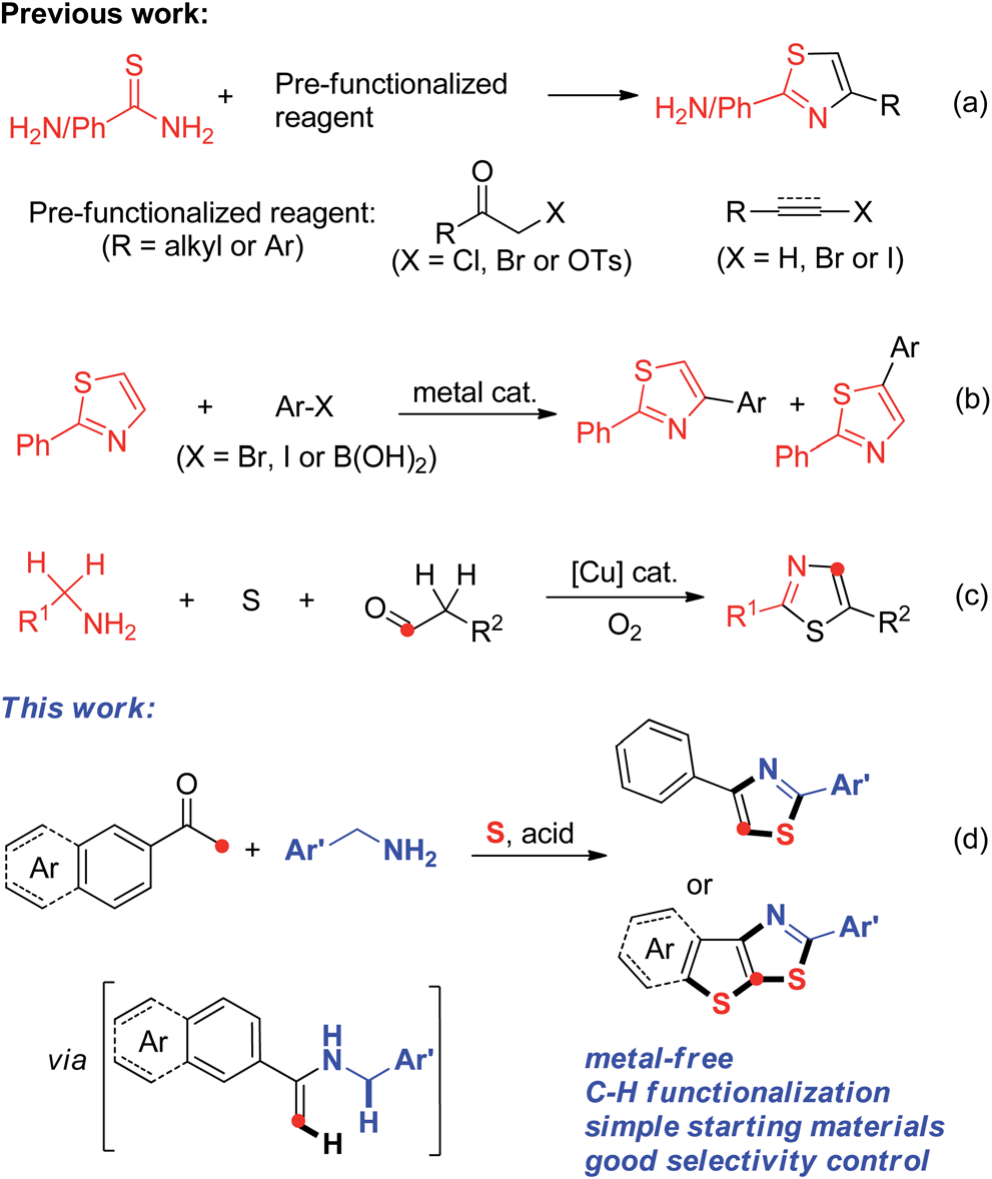
Synthesis of 2,4-disubstituted thiazoles.

We commenced our investigation using acetophenone (1a), benzylamine (2a), and sulfur powder as the model system (Table 1). When the reaction was performed using formic acid as the additive at 130 °C in DMSO (dimethyl sulfoxide) for 8 h, the desired product 3aa was obtained in a 24% yield (Table 1, entry 1). Then, a series of Brønsted acid reagents including HOAc, TFA (trifluoroacetic acid), TsOH (*p*-toluene sulfonic acid), MsOH (methanesulfonic acid), PivOH (trimethylacetic acid), benzoic acid, nicotinic acid, and isonicotinic acid were investigated (Table 1, entries 2–9). Among them, isonicotinic acid was the preferable additive for this reaction to give 3aa in a 66% yield (Table 1, entry 9). A sharp decline in the reaction yield was observed when DMF (*N*,*N*-dimethylformamide), DMAc (*N*,*N*-dimethylacetamide), NMP (*N*-methyl pyrrolidone), toluene, PhCl, and 1,4-dioxane were used as the solvents (Table 1, entries 10–15). Increasing the amount of sulfur powder or decreasing the reaction temperature both led to a lower yield of the product (entries 16–17). Meanwhile, the reaction atmosphere, such as Ar and O_2_, provided the target product in 62% and 34% yields, respectively (entries 18–19). Furthermore, only a 13% yield of the sulfuration product was observed in the absence of acid additives (entry 20).

**Table 1:** Optimization of the reaction conditions^a^

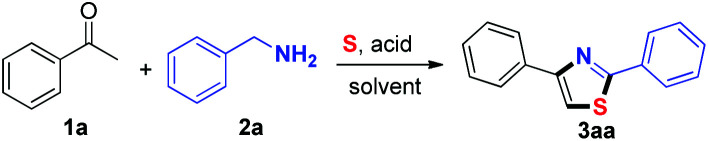			
Entry	Acid	Solvent	Yield^b^ (%)
1	Formic acid	DMSO	24
2	HOAc	DMSO	33
3	TFA	DMSO	n.d.
4	TsOH	DMSO	n.d.
5	MsOH	DMSO	n.d.
6	PivOH	DMSO	45
7	Benzoic acid	DMSO	28
8	Nicotinic acid	DMSO	54
9	Isonicotinic acid	DMSO	66
10	Isonicotinic acid	DMF	n.d.
11	Isonicotinic acid	DMAc	n.d.
12	Isonicotinic acid	NMP	n.d.
13	Isonicotinic acid	Toluene	n.d.
14	Isonicotinic acid	PhCl	n.d.
15	Isonicotinic acid	1,4-Dioxane	Trace
16^c^	Isonicotinic acid	DMSO	58
17^d^	Isonicotinic acid	DMSO	47
18^e^	Isonicotinic acid	DMSO	62
19^f^	Isonicotinic acid	DMSO	34
20		DMSO	13

a Reaction conditions: 1a (0.2 mmol), 2a (0.4 mmol), acid (0.2 mmol), S (0.4 mmol), solvent (0.6 mL), 130 °C, 8 h, under air atmosphere.

b GC yield using dodecane as the internal standard. n.d. means not detected.

c S (0.6 mmol, 3 equiv.).

d 120 °C.

e Under an argon atmosphere.

f Under an oxygen atmosphere.

Under the optimized reaction conditions, the generality of the sulfuration/annulation reaction cascade to the synthesized thiazoles was investigated (Table 2). The model reaction of 1a and 2a in the presence of sulfur powder afforded 3aa in a 63% isolated yield. Similar yields were obtained when methyl, butyl, phenyl, and methoxy substituents were located at the *para* position of the phenyl ring (3ab–3af). The results showed that the substrates with halogen functional groups (F, Cl, Br, and even I) on the phenyl ring were compatible for this transformation (3ai–3al, 3ar–3at, and 3av–3aw). The substrates bearing strong electron-withdrawing groups (–OCF_3_, –CO_2_CH_3_, –CN, –NO_2_, and –SO_2_CH_3_) were also compatible with the reaction conditions, providing the corresponding products in good yields (3ag–3ah, 3am–3ao and 3au). Acetophenones 2x and 2y bearing two substituents reacted smoothly to yield the desired products 3ax and 3ay, respectively. It should be noted that bis-heteroannulation products (3az–3ac′) could be achieved when aromatic fused ring ketones (2z–2c′) were used as the substrates under optimal conditions. In this type of reaction, two sulfur atoms were incorporated into the heterocycles and four C–S bonds were selectively formed in one pot. Aliphatic ketones such as 1-cyclohexylethanone and 3-methylbutan-2-one both failed to afford the desired product under the current reaction conditions. We also used non-methyl ketones such as propiophenone and 1,2-diphenylethan-1-one. However, we did not observe the target products.

**Table 2:** Substrate scope with respect to ketones^a^

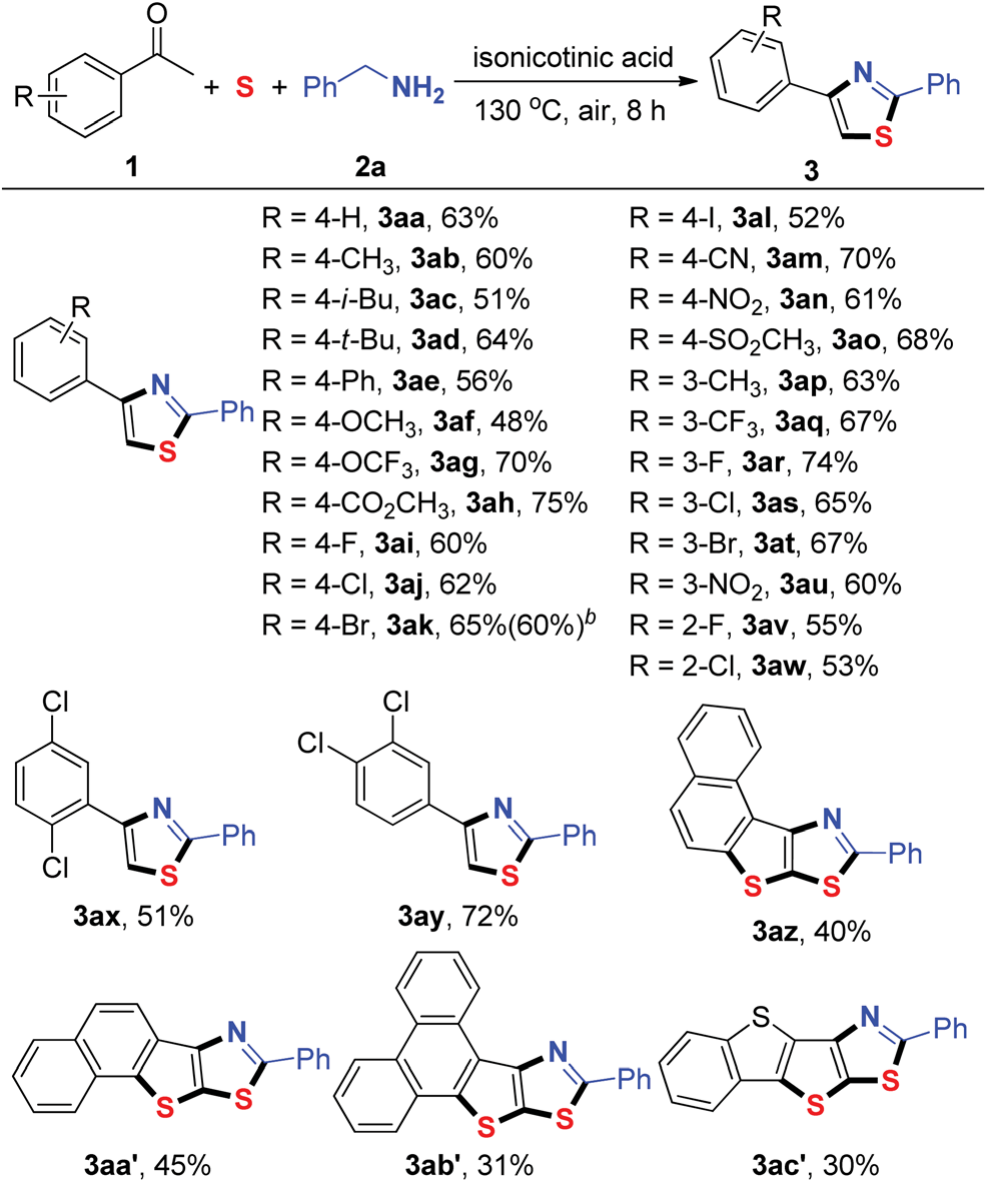

a Reaction conditions: 1 (0.2 mmol), 2a (0.4 mmol), S (0.4 mmol), isonicotinic acid (0.2 mmol), DMSO (0.6 mL), 130 °C, 8 h, under an air atmosphere, isolated yield based on 1.

b Yield of 10 mmol scale reaction.

Subsequently, various substituted benzylamines were examined under the optimized reaction conditions (Table 3). First, a series of *para*-substituted benzylamines, including electron-donating groups and electron-withdrawing groups, were converted into the corresponding thiazoles (3ba–3ga) in good yields. Furthermore, *meta* and *ortho*-substituted benzylamines were able to give the desired products in moderate to good yields (3ha–3ka). Bis-substituted benzylamines such as 3-chloro-4-fluorobenzylamine (2l) and (3,5-difluorophenyl)methanamine (2m) also successfully participated in this oxidative cyclization process, affording the desired products 3la and 3ma in 51% and 67% yields, respectively. Notably, 4-pyridinemethaneamine (2n) also reacted efficiently to afford the corresponding 3na in a moderate yield. Unfortunately, aliphatic amines failed to afford the desired product.

**Table 3:** Substrate scope with respect to benzylamines^a^

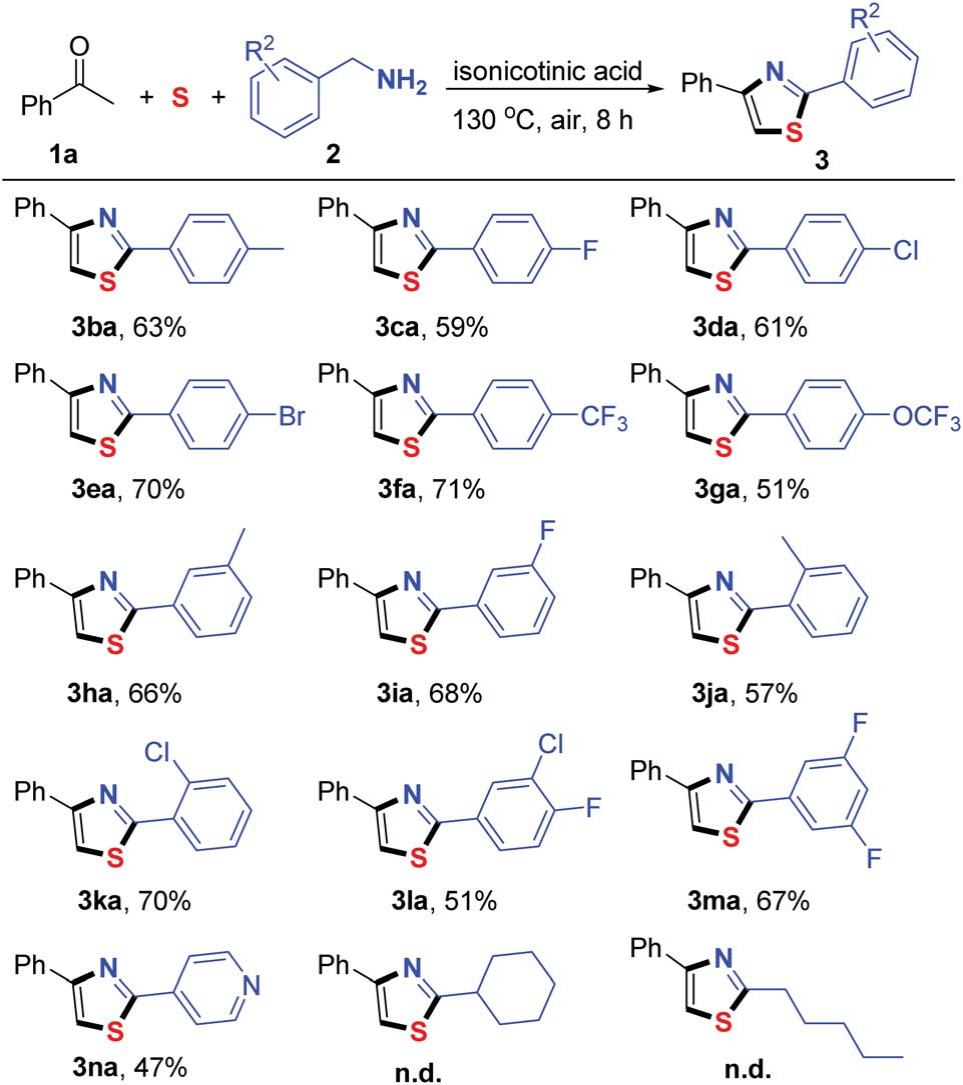

a Reaction conditions: 1 (0.2 mmol), 2a (0.4 mmol), S (0.4 mmol), isonicotinic acid (0.2 mmol), DMSO (0.6 mL), 130 °C, 8 h, under an air atmosphere, isolated yield based on 1a.

In order to understand the reaction mechanism, several control experiments were designed under different conditions (Scheme 2). The reaction of acetophenone 1a and benzothioamide 5 only yielded a trace amount of the desired product under the optimal conditions. No reaction occurred in the absence of sulfur powder (Scheme 2a). Similarly, the replacement of benzothioamide with benzamide 6 did not give the thiazole product (Scheme 2b). The treatment of *N*-(1-phenylethyl)benzothioamide 7, (*E*)-1-phenyl-*N*-(1-phenylethylidene)methanamine 8 and (*E*)-*N*-benzylidene-1-phenylethanamine 9 under the optimal reaction conditions did not afford the target product (Scheme 2c–e). However, imines 8 and 9 under the optimal conditions without isonicotinic acid provided the target product with 37% and trace amount yields, respectively (Scheme 2d and e).

**Scheme 2:**
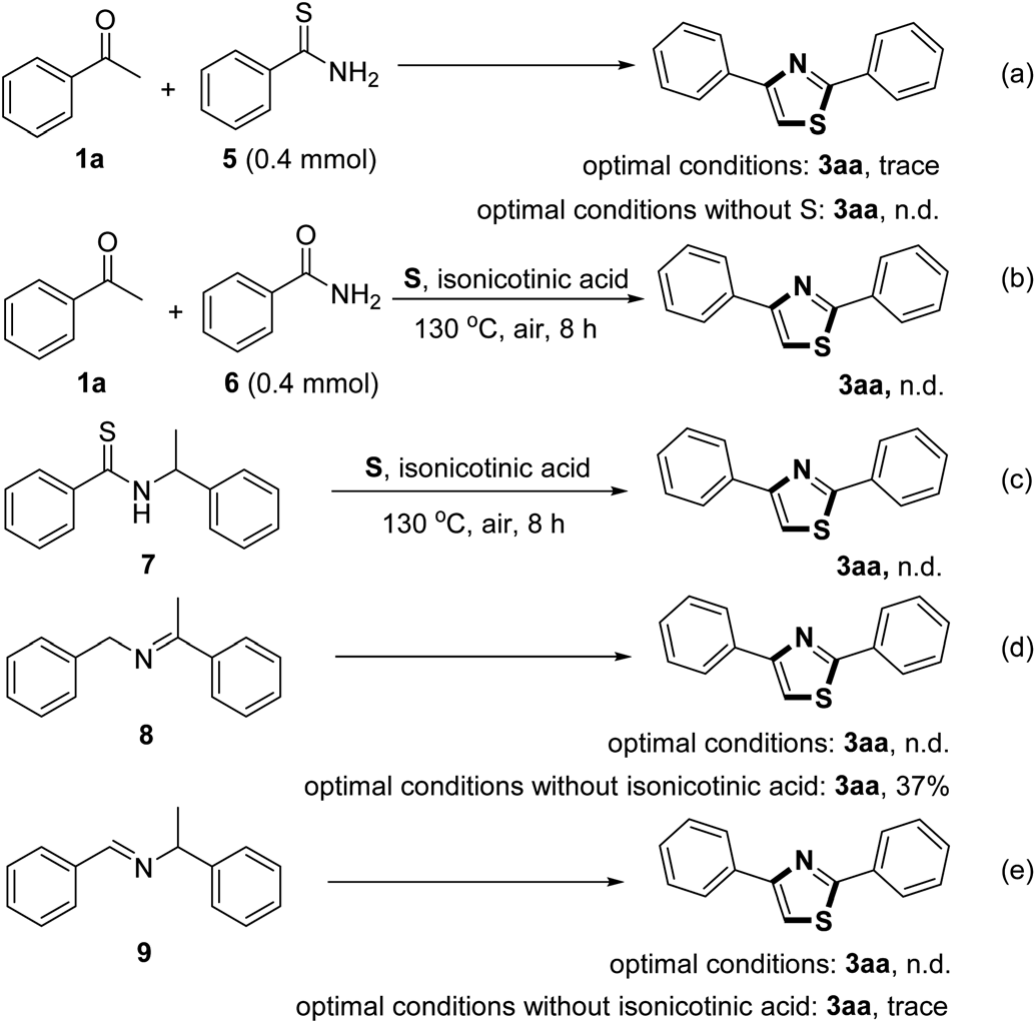
Control experiments.

On the basis of the experimental observations and previous reports,^17,18*f*,19*d*^ a possible reaction mechanism is proposed (Scheme 3). The dehydrative condensation of acetophenone (1a) and benzylamine (2a) should be the initial step, which affords the imine intermediate 8. The tautomerization of the intermediate 8 generates enamine A. Subsequently, the interaction of A and elemental sulfur delivers the poly-sulfur intermediate B through Willgerodt–Kindler type oxidation.^20^ Further oxidation and deprotonation of B affords the intermediate C. Then, intramolecular attack occurs to release S_*n*−1_ and generate the intermediate D, which finally furnishes the product 3aa by the oxidation process.

**Scheme 3:**
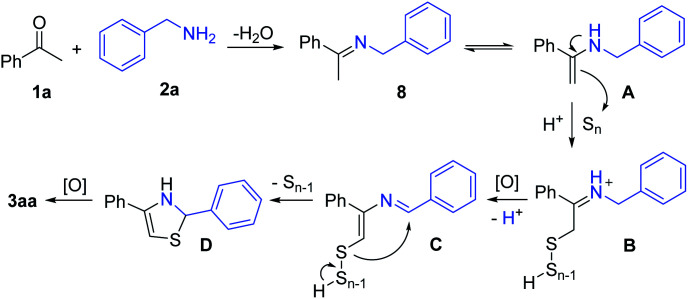
Possible reaction mechanism.

In summary, we have developed a novel Brønsted acid-promoted protocol for the synthesis of 2,4-disubstituted thiazoles from benzylamines, acetophenones, and sulfur powder under metal-free conditions. The cheap and readily available sulfur powder acted as the sulfur source to selectively assemble the thiazole derivatives. This reaction represents effective access to thiazoles from readily available starting materials with good functional group tolerance. Further studies on the mechanism are ongoing in our laboratory.

## Conflicts of interest

There are no conflicts to declare.

## Supplementary Material

RA-010-C9RA09656F-s001
