# Supplementary material for: Brønsted acid-promoted thiazole synthesis under metal-free conditions using sulfur powder as the sulfur source
Source: RSC Adv. 2020 Jan 23;10(7):3931–5. doi: 10.1039/c9ra09656f (PMC9048575; doi:10.1039/c9ra09656f)

Electronic Supplementary Material (ESI) for RSC Advances.
This journal is © The Royal Society of Chemistry 2020

Supporting Information

Brønsted acid-promoted thiazoles synthesis under metal-free conditions using sulfur powder as the sulfur source

Penghui Ni,[a] Jing Tan,[a] Rong li,[a] Huawen Huang,[a] Feng Zhang,[b]* Guo-Jun Deng[a]*

[a]Key Laboratory for Green Organic Synthesis and Application of Hunan Province, Key Laboratory of Environmentally Friendly Chemistry and Application of Ministry of Education, College of Chemistry, Xiangtan University, Xiangtan 411105, China. E-mail: gjdeng@xtu.edu.cn.

[b]College of Science, Hunan Agricultural University, Changsha, 410128, China. E-mail: zhangf@iccas.ac.cn.

Table of Contents

1. General information 2

2. General procedure for synthesis of 3aa 2

3. Procedure for gram-scale reaction 2

4. Characterization data of products 3

5. References 24

6. Copies of ^{1}H, ^{13}C and ^{19}F NMR spectra of all products 25

1. General information

All reactions were carried out under an atmosphere of air unless otherwise noted. Column chromatography was performed using silica gel (neutral) (200-300 mesh). ^{1}H, ^{13}C and ^{19}F NMR spectra were recorded on Bruker-AV (400, 100 and 376 MHz, respectively) instrument internally referenced to tetramethylsilane (TMS) or chloroform signals. Mass spectra were measured on Agilent 5975 GC-MS instrument (EI). High-resolution mass spectra were recorded at Keecloud (Shanghai) Biotechnology co. LTD. HRMS was conducted using electrospraying ionization (ESI) and was performed on a Thermo Scientific LTQ Orbitrap XL. The structures of known compounds were further corroborated by comparing their ^{1}H, ^{13}C, ^{19}F NMR data and MS data with those of literature. All reagents were obtained from commercial suppliers and used without further purification. The molecular weight of S is determined to be 32 g/mol unless otherwise noted.

2. General procedure for synthesis of 3aa

Isonicotinic acid (24.6 mg, 0.2 mmol) and sulfur powder (12.8 mg, 0.4 mmol) were added to an oven-dried reaction vessel (20 mL). The reaction vessel was sealed, acetophenone (**1a**, 24.0 μL, 0.2 mmol), benzylamine (**2a**, 44.0 μL, 0.4 mmol) and dimethyl sulfoxide (0.6 mL) were added by syringe. The reaction vessel was stirred at 130 °C for 8 h under air atmosphere. After cooling to room temperature, the reaction was diluted with ethyl acetate (20 mL) and washed with saturated salt water. The organic layer was separated, and the aqueous layer was extracted with ethyl acetate for three times. The combined organic layer was dried over sodium sulfate and the volatiles were removed under reduced pressure. The residue was purified by column chromatography on silica gel (petroleum ether/EtOAc = 400:1) to yield the desired product **3aa** as white solid (29.9 mg, 63% yield), mp 96-97 °C, R_f = 0.70 (100:1 petroleum ether/EtOAc).

3. Procedure for gram-scale reaction

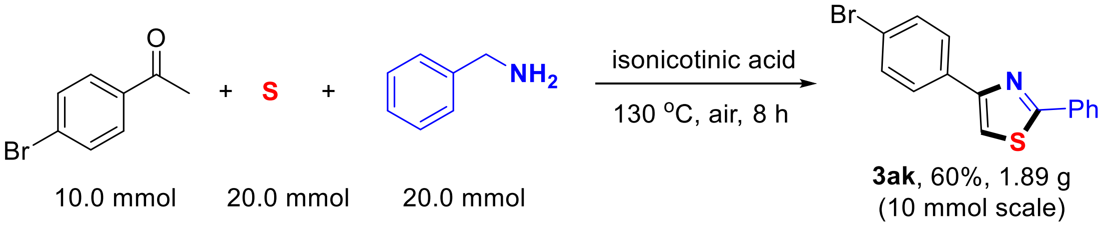

Isonicotinic acid (2.5 g, 20 mmol), 4'-bromoacetophenone (2.0 g, 10 mmol) and sulfur powder

(0.64 g, 20 mmol) were added to a round bottomed flask (50 mL). Benzylamine (2.2 mL, 20 mmol) and dimethyl sulfoxide (15 mL) were added by measuring cylinder. The reaction vessel was stirred at 130 °C for 8 h under air atmosphere. After cooling to room temperature, the reaction was diluted with ethyl acetate (25 mL) and washed with saturated salt water. The organic layer was separated, and the aqueous layer was extracted with ethyl acetate for three times. The combined organic layer was dried over sodium sulfate and the volatiles were removed under reduced pressure. The residue was purified by column chromatography on silica gel (petroleum ether/EtOAc = 400:1) to yield the desired product **3ak** as white solid (1.89 g, 60% yield), mp 126-128 °C. R_f = 0.60 (100:1 petroleum ether/EtOAc).

4. Characterization data of products

2,4-Diphenylthiazole (3aa, CAS: 1826-14-8)[1]

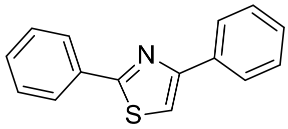

^{1}H NMR (400 MHz, $CDCl_3$, ppm) δ 8.03 (dd, J = 7.8, 1.6 Hz, 2H), 8.02 – 7.98 (m, 2H), 7.47 – 7.41 (m, 6H), 7.36 – 7.32 (m, 1H); ^{13}C NMR (100 MHz, ppm) δ 167.8, 156.2, 134.5, 133.7, 130.0, 128.9, 128.7, 128.1, 126.6, 126.4, 112.6.

2-Phenyl-4-(*p*-tolyl)thiazole (3ab, CAS: 2362-58-5)[1]

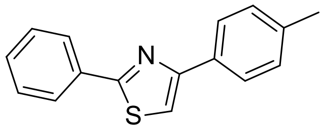

The reaction was conducted with 4'-methylacetophenone (**1b**, 27.0 µL, 0.2 mmol), benzylamine (**2a**, 44.0 µL, 0.4 mmol), isonicotinic acid (24.6 mg, 0.2 mmol) and sulfur powder (12.8 mg, 0.4 mmol). The residue was purified by column chromatography on silica gel (petroleum ether/EtOAc = 400:1) to yield the desired product **3ab** as white solid (30.1 mg, 60% yield), mp 109-110 °C. R_f = 0.70 (100:1 petroleum ether/EtOAc).

^{1}H NMR (400 MHz, $CDCl_3$, ppm) δ 8.06 – 8.03 (m, 2H), 7.89 (d, J = 8.2 Hz, 2H), 7.46 – 7.42 (m, 4H), 7.26 – 7.24 (m, 2H), 2.40 (s, 3H); ^{13}C NMR (100 MHz, ppm) δ 167.8, 156.3, 138.0, 133.7, 131.7, 130.0, 129.4, 128.9, 126.6, 126.4, 111.9, 21.3.

4-(4-Isobutylphenyl)-2-phenylthiazole (3ac)

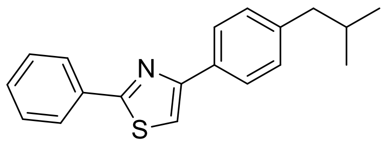

The reaction was conducted with 4'-(2-methylpropyl)acetophenone (**2c**, 38.0 μL, 0.2 mmol), benzylamine (**2a**, 44.0 μL, 0.4 mmol), isonicotinic acid (24.6 mg, 0.2 mmol) and sulfur powder (12.8 mg, 0.4 mmol). The residue was purified by column chromatography on silica gel (petroleum ether/EtOAc = 300:1) to yield the desired product **3ac** as white solid (29.9 mg, 51% yield), mp 91-92 °C. R_f = 0.80 (100:1 petroleum ether/EtOAc).

^{1}H NMR (400 MHz, $CDCl_3$, ppm) δ 8.04 (dd, J = 7.8, 1.6 Hz, 2H), 7.90 (d, J = 8.2 Hz, 2H), 7.47 – 7.40 (m, 4H), 7.23 – 7.20 (m, 2H), 2.51 (d, J = 7.2 Hz, 2H), 1.94 – 1.84 (m, 1H), 0.92 (d, J = 6.6 Hz, 6H); ^{13}C NMR (100 MHz, $CDCl_3$, ppm) δ 167.7, 156.4, 141.8, 133.7, 132.0, 129.9, 129.5, 128.9, 126.6, 126.2, 111.9, 45.2, 30.2, 22.3; HRMS (ESI) m/z calcd. for $C_{19}H_{20}NS^+$ $(M+H)^+$ 294.1311, found 294.1313.

4-(4-(*tert*-Butyl)phenyl)-2-phenylthiazole (3ad)[2]

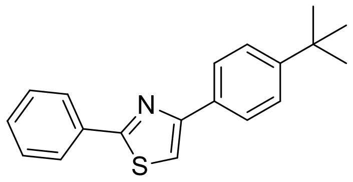

The reaction was conducted with 4'-*tert*-butylacetophenone (**2d**, 37.0 μL, 0.2 mmol), benzylamine (**2a**, 44.0 μL, 0.4 mmol), isonicotinic acid (24.6 mg, 0.2 mmol) and sulfur powder (12.8 mg, 0.4 mmol). The residue was purified by column chromatography on silica gel (petroleum ether/EtOAc = 300:1) to yield the desired product **3ad** as yellow solid (37.5 mg, 64% yield), mp 93-95 °C. R_f = 0.80 (100:1 petroleum ether/EtOAc).

^{1}H NMR (400 MHz, $CDCl_3$, ppm) δ 8.05 – 8.02 (m, 2H), 7.91 (d, J = 8.4 Hz, 2H), 7.47 – 7.41 (m, 5H), 7.40 (s, 1H), 1.35 (s, 9H); ^{13}C NMR (100 MHz, $CDCl_3$, ppm) δ 167.7, 156.3, 151.2, 133.8, 131.8, 129.9, 128.9, 126.6, 126.2, 125.6, 112.0, 34.6, 31.3.

4-([1,1'-Biphenyl]-4-yl)-2-phenylthiazole (3ae, CAS: 13355-29-8)[2]

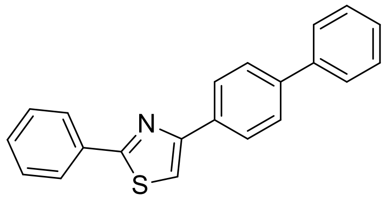

The reaction was conducted with 4-acetylbiphenyl (**2e**, 40.0 mg, 0.2 mmol), benzylamine (**2a**, 44.0 μL, 0.4 mmol), isonicotinic acid (24.6 mg, 0.2 mmol) and sulfur powder (12.8 mg, 0.4 mmol). The residue was purified by column chromatography on silica gel (petroleum ether/EtOAc = 300:1) to yield the desired product **3ae** as yellow solid (35.1 mg, 56% yield), mp 158-160 ^{o}C. R_f = 0.70 (100:1 petroleum ether/EtOAc).

^{1}H NMR (400 MHz, $CDCl_3$, ppm) δ 8.07 – 8.05 (m, 4H), 7.69 – 7.64 (m, 4H), 7.49 – 7.43 (m, 6H), 7.37 – 7.34 (m, 1H); ^{13}C NMR (100 MHz, $CDCl_3$, ppm) δ 167.9, 155.9, 140.8, 140.7, 133.7, 133.5, 130.1, 128.9, 128.8, 127.4, 127.0, 126.8, 126.6, 112.6.

4-(4-Methoxyphenyl)-2-phenylthiazole (3af, CAS: 2362-68-7)[3]

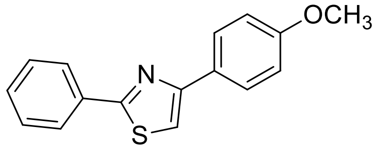

The reaction was conducted with 4'-methoxyacetophenone (**2f**, 30.0 mg, 0.2 mmol), benzylamine (**2a**, 44.0 μL, 0.4 mmol), isonicotinic acid (24.6 mg, 0.2 mmol) and sulfur powder (12.8 mg, 0.4 mmol). The residue was purified by column chromatography on silica gel (petroleum ether/EtOAc = 30:1) to yield the desired product **3af** as yellow solid (25.6 mg, 48% yield), mp 118-120 ^{o}C. R_f = 0.60 (10:1 petroleum ether/EtOAc).

^{1}H NMR (400 MHz, $CDCl_3$, ppm) δ 8.06 – 8.00 (m, 2H), 7.92 (d, J = 8.6 Hz, 2H), 7.51 – 7.40 (m, 3H), 7.32 (s, 1H), 6.96 (d, J = 8.7 Hz, 2H), 3.84 (s, 3H); ^{13}C NMR (100 MHz, $CDCl_3$, ppm) δ 167.7, 159.6, 156.0, 133.8, 129.9, 128.9, 127.7, 127.5, 126.5, 114.0, 110.9, 55.3.

2-Phenyl-4-(4-(trifluoromethoxy)phenyl)thiazole (3ag)

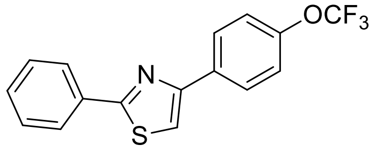

The reaction was conducted with 4'-(trifluoromethoxy)acetophenone (**2g**, 33.0 μL, 0.2 mmol), benzylamine (**2a**, 44.0 μL, 0.4 mmol), isonicotinic acid (24.6 mg, 0.2 mmol) and sulfur powder (12.8 mg, 0.4 mmol). The residue was purified by column chromatography on silica gel (petroleum ether/EtOAc = 200:1) to yield the desired product **3ag** as white solid (44.9 mg, 70% yield), mp 100-101 °C. R_f = 0.60 (100:1 petroleum ether/EtOAc).

^{1}H NMR (400 MHz, $CDCl_3$, ppm) δ 8.03 – 7.98 (m, 4H), 7.47 – 7.43 (m, 4H), 7.27 (d, J = 8.2 Hz, 2H); ^{13}C NMR (100 MHz, $CDCl_3$, ppm) δ 168.2, 154.8, 149.0, 133.5, 133.2, 130.2, 128.9, 127.8, 126.6, 121.2, 120.5 (q, J = 257.0 Hz), 113.1; ^{19}F NMR (376 MHz, $CDCl_3$, ppm) δ -57.6; HRMS (ESI) m/z calcd. for $C_{16}H_{11}F_3NOS^+$ $(M+H)^+$ 322.0508, found 322.0510.

Methyl-4-(2-phenylthiazol-4-yl)benzoate (3ah)[3]

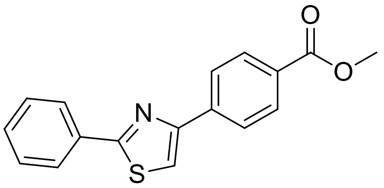

The reaction was conducted with methyl 4-acetylbenzoate (**2h**, 36.0 mg, 0.2 mmol), benzylamine (**2a**, 44.0 μL, 0.4 mmol), isonicotinic acid (24.6 mg, 0.2 mmol) and sulfur powder (12.8 mg, 0.4 mmol). The residue was purified by column chromatography on silica gel (petroleum ether/EtOAc = 20:1) to yield the desired product **3ah** as light yellow solid (44.3 mg, 75% yield), mp 151-152 °C. R_f = 0.60 (10:1 petroleum ether/EtOAc).

^{1}H NMR (400 MHz, $CDCl_3$, ppm) δ 8.18 – 7.97 (m, 6H), 7.58 (s, 1H), 7.51 – 7.40 (m, 3H), 3.94 (s, 3H); ^{13}C NMR (100 MHz, $CDCl_3$, ppm) δ 168.2, 166.8, 155.0, 138.5, 133.4, 130.2, 130.1, 129.4, 128.9, 126.6, 126.2, 114.5, 52.1.

4-(4-Fluorophenyl)-2-phenylthiazole (3ai, CAS: 329079-72-3)[2]

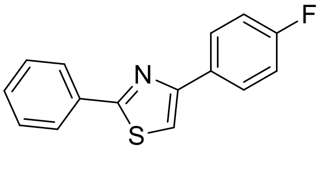

The reaction was conducted with 4'- fluoroacetophenone (**2i**, 24.0 μL, 0.2 mmol), benzylamine (**2a**, 44.0 μL, 0.4 mmol), isonicotinic acid (24.6 mg, 0.2 mmol) and sulfur powder (12.8 mg, 0.4 mmol). The residue was purified by column chromatography on silica gel (petroleum ether/EtOAc =

300:1) to yield the desired product **3ai** as white solid (30.6 mg, 60% yield), mp 104-105 °C. R_f = 0.70 (100:1 petroleum ether/EtOAc).

^{1}H NMR (400 MHz, $CDCl_3$, ppm) δ 8.03 – 8.00 (m, 2H), 7.98 – 7.93 (m, 2H), 7.48 – 7.42 (m, 3H), 7.38 (s, 1H), 7.15 – 7.09 (m, 2H); ^{13}C NMR (100 MHz, $CDCl_3$, ppm) δ 168.0, 162.7 (d, J = 247.4 Hz), 155.2, 133.6, 130.7, 130.7 (d, J = 3.2 Hz), 128.9, 128.1 (d, J = 8.1 Hz), 126.5, 115.6 (d, J = 21.7 Hz), 112.2; ^{19}F NMR (376 MHz, $CDCl_3$, ppm) δ -113.7.

4-(4-Chlorophenyl)-2-phenylthiazole (3aj, CAS: 122395-24-8)[2]

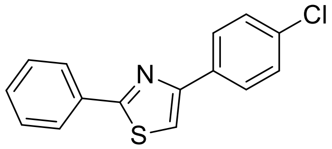

The reaction was conducted with 4'-chloroacetophenone (**2j**, 26.0 μL, 0.2 mmol), benzylamine (**2a**, 44.0 μL, 0.4 mmol), isonicotinic acid (24.6 mg, 0.2 mmol) and sulfur powder (12.8 mg, 0.4 mmol). The residue was purified by column chromatography on silica gel (petroleum ether/EtOAc = 300:1) to yield the desired product **3aj** as white solid (33.6 mg, 62% yield), mp 133-134 °C. R_f = 0.70 (100:1 petroleum ether/EtOAc).

^{1}H NMR (400 MHz, $CDCl_3$, ppm) δ 8.02 – 8.00 (m, 2H), 7.91 (d, J = 8.5 Hz, 2H), 7.48 – 7.38 (m, 6H).; ^{13}C NMR (100 MHz, $CDCl_3$, ppm) δ 168.1, 155.0, 133.9, 133.5, 132.9, 130.2, 128.9, 128.9, 127.7, 126.6, 112.9.

4-(4-Bromophenyl)-2-phenylthiazole (3ak CAS: 2362-69-8)[2]

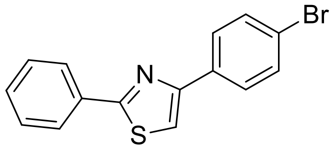

The reaction was conducted with 4'-bromoacetophenone (**2k**, 40.0 mg, 0.2 mmol), benzylamine (**2a**, 44.0 μL, 0.4 mmol), isonicotinic acid (24.6 mg, 0.2 mmol) and sulfur powder (12.8 mg, 0.4 mmol). The residue was purified by column chromatography on silica gel (petroleum ether/EtOAc = 400:1) to yield the desired product **3ak** as white solid (40.9 mg, 65% yield), mp 126-128 °C. R_f = 0.60 (100:1 petroleum ether/EtOAc).

^{1}H NMR (400 MHz, $CDCl_3$, ppm) δ 8.02 – 8.00 (m, 2H), 7.86 – 7.83 (m, 2H), 7.56 – 7.53 (m, 2H),

7.47 – 7.43 (m, 4H); ^{13}C NMR (100 MHz, $CDCl_3$, ppm) δ 168.1, 155.0, 133.45, 133.4, 131.8, 130.2, 128.9, 127.9, 126.6, 122.1, 113.0.

4-(4-Iodophenyl)-2-phenylthiazole (3al, CAS: 2227-65-8)[2]

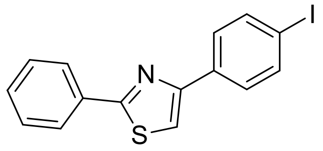

The reaction was conducted with 4'-iodoacetophenone (**2l**, 50.0 mg, 0.2 mmol), benzylamine (**2a**, 44.0 μL, 0.4 mmol), isonicotinic acid (24.6 mg, 0.2 mmol) and sulfur powder (12.8 mg, 0.4 mmol). The residue was purified by column chromatography on silica gel (petroleum ether/EtOAc = 400:1) to yield the desired product **3al** as white solid (37.8 mg, 52% yield), mp 114-116 °C. R_f = 0.60 (100:1 petroleum ether/EtOAc).

^{1}H NMR (400 MHz, $CDCl_3$, ppm) δ 8.02 – 8.00 (m, 2H), 7.76 – 7.70 (m, 4H), 7.45 – 7.44 (m, 4H); ^{13}C NMR (100 MHz, $CDCl_3$, ppm) δ 168.1, 155.1, 137.8, 133.9, 133.5, 130.2, 128.9, 128.1, 126.6, 113.1, 93.8.

4-(2-Phenylthiazol-4-yl)benzonitrile (3am)[4]

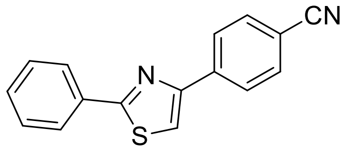

The reaction was conducted with 4'-cyanoacetophenone (**2m**, 29.0 mg, 0.2 mmol), benzylamine (**2a**, 44.0 μL, 0.4 mmol), isonicotinic acid (24.6 mg, 0.2 mmol) and sulfur powder (12.8 mg, 0.4 mmol). The residue was purified by column chromatography on silica gel (petroleum ether/EtOAc = 15:1) to yield the desired product **3am** as yellow solid (36.7 mg, 70% yield), mp 135-137 °C. R_f = 0.60 (10:1 petroleum ether/EtOAc).

^{1}H NMR (400 MHz, $CDCl_3$, ppm) δ 8.06 (d, J = 8.4 Hz, 2H), 8.01 – 7.98 (m, 2H), 7.68 (d, J = 8.4 Hz, 2H), 7.57 (s, 1H), 7.48 – 7.45 (m, 3H); ^{13}C NMR (100 MHz, $CDCl_3$, ppm) δ 168.5, 154.0, 138.3, 133.1, 132.5, 130.4, 128.9, 126.7, 126.5, 118.9, 115.3, 111.2.

4-(4-Nitrophenyl)-2-phenylthiazole (3an, CAS: 2521-25-7)[5]

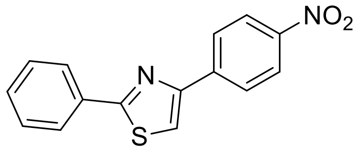

The reaction was conducted with 4'-nitroacetophenone (**2n**, 33.5 mg, 0.2 mmol), benzylamine (**2a**, 44.0 μL, 0.4 mmol), isonicotinic acid (24.6 mg, 0.2 mmol) and sulfur powder (12.8 mg, 0.4 mmol). The residue was purified by column chromatography on silica gel (petroleum ether/EtOAc = 10:1) to yield the desired product **3an** as yellow solid (34.4 mg, 61% yield), mp 123-124 °C. R_f = 0.70 (8:1 petroleum ether/EtOAc).

^{1}H NMR (400 MHz, $CDCl_3$, ppm) *δ* 8.27 (d, *J* = 8.9 Hz, 2H), 8.12 (d, *J* = 8.8 Hz, 2H), 8.03 – 8.00 (m, 2H), 7.64 (s, 1H), 7.48 – 7.45 (m, 3H); ^{13}C NMR (100 MHz, $CDCl_3$, ppm) *δ* 168.7, 153.6, 147.1, 140.2, 133.1, 130.5, 129.0, 126.9, 126.6, 124.1, 116.0.

4-(4-(Methylsulfonyl)phenyl)-2-phenylthiazole (3ao)

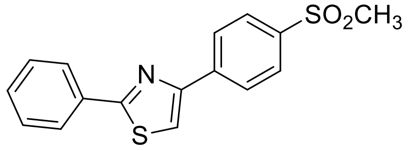

The reaction was conducted with 4'-methylsulphonylacetophenone (**2o**, 40.0 mg, 0.2 mmol), benzylamine (**2a**, 44.0 μL, 0.4 mmol), isonicotinic acid (24.6 mg, 0.2 mmol) and sulfur powder (12.8 mg, 0.4 mmol). The residue was purified by column chromatography on silica gel (petroleum ether/EtOAc = 5:1) to yield the desired product **3ao** as red solid (42.8 mg, 68% yield), mp 192-193 °C. R_f = 0.50 (3:1 petroleum ether/EtOAc).

^{1}H NMR (400 MHz, $CDCl_3$, ppm) *δ* 8.19 (d, *J* = 8.4 Hz, 2H), 8.05 –8.00 (m, 4H), 7.65 (s, 1H), 7.49 – 7.47 (m, 3H), 3.09 (s, 3H); ^{13}C NMR (100 MHz, $CDCl_3$, ppm) *δ* 168.6, 154.0, 139.5, 139.4, 133.2, 130.4, 129.0, 127.9, 127.1, 126.6, 115.5, 44.6; HRMS (ESI) m/z calcd. for for $C_{16}H_{14}NO_2S_2^+$ $(M+H)^+$ 316.0461, found 316.0465.

2-Phenyl-4-(*m*-tolyl)thiazole (3ap)

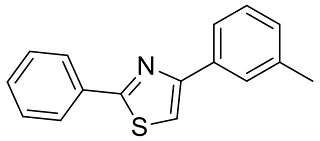

The reaction was conducted with 3'-methylacetophenone (**2p**, 26.0 μL, 0.2 mmol), benzylamine (**2a**, 44.0 μL, 0.4 mmol), isonicotinic acid (24.6 mg, 0.2 mmol) and sulfur powder (12.8 mg, 0.4 mmol). The residue was purified by column chromatography on silica gel (petroleum ether/EtOAc = 400:1) to yield the desired product **3ap** as colourless oily liquid (31.6 mg, 63% yield). R_f = 0.80 (100:1 petroleum ether/EtOAc).

^{1}H NMR (400 MHz, $CDCl_3$, ppm) δ 8.04 (dd, J = 8.0, 1.6 Hz, 2H), 7.83 (s, 1H), 7.76 (d, J = 7.8 Hz, 1H), 7.47 – 7.41 (m, 4H), 7.32 (t, J = 7.6 Hz, 1H), 7.16 (d, J = 7.6 Hz, 1H), 2.42 (s, 3H); ^{13}C NMR (100 MHz, $CDCl_3$, ppm) δ 167.8, 156.4, 138.3, 134.4, 133.7, 130.0, 128.9, 128.9, 128.6, 127.2, 126.6, 123.5, 112.5, 21.5; HRMS (ESI) m/z calcd. for $C_{16}H_{14}NS^+$ $(M+H)^+$ 252.0842, found 252.0843.

2-Phenyl-4-(3-(trifluoromethyl)phenyl)thiazole (3aq)

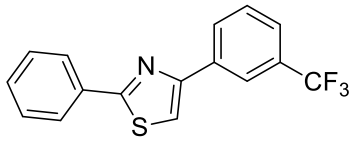

The reaction was conducted with 3′-(trifluoromethyl)acetophenone (**2q**, 30.0 μL, 0.2 mmol), benzylamine (**2a**, 44.0 μL, 0.4 mmol), isonicotinic acid (24.6. mg, 0.2 mmol) and sulfur powder (12.8 mg, 0.4 mmol). The residue was purified by column chromatography on silica gel (petroleum ether/EtOAc = 400:1) to yield the desired product **3aq** as white solid (40.9 mg, 67% yield), mp 112-114 °C. R_f = 0.70 (100:1 petroleum ether/EtOAc).

^{1}H NMR (400 MHz, $CDCl_3$, ppm) δ 8.26 (s, 1H), 8.13 (d, J = 7.6 Hz, 1H), 8.04 –8.02 (m, 2H), 7.59 (d, J = 7.8 Hz, 1H), 7.54 (d, J = 7.7 Hz, 1H), 7.51 (s, 1H), 7.48 – 7.43 (m, 3H); ^{13}C NMR (100 MHz, $CDCl_3$, ppm) δ 168.3, 154.6, 135.1, 133.4, 131.1 (q, J = 32.3 Hz), 130.3, 129.5, 129.2, 129.0, 126.6, 124.6 (q, J = 3.7 Hz), 124.1 (q, J = 270.8 Hz), 123.2 (q, J = 3.8 Hz), 113.8; ^{19}F NMR (376 MHz, $CDCl_3$, ppm) δ -62.6; HRMS calcd. for $C_{16}H_{11}F_3NS^+$ $(M+H)^+$ 306.0559, found 306.0558.

4-(3-Fluorophenyl)-2-phenylthiazole (3ar)

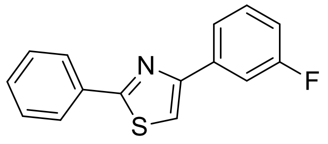

The reaction was conducted with 3'-fluoroacetophenone (**2r**, 24.0 μL, 0.2 mmol), benzylamine (**2a**, 44.0 μL, 0.4 mmol), isonicotinic acid (24.6 mg, 0.2 mmol) and sulfur powder (12.8 mg, 0.4 mmol). The residue was purified by column chromatography on silica gel (petroleum ether/EtOAc = 400:1) to yield the desired product **3ar** as white solid (37.7 mg, 74% yield), mp 85-86 °C. R_f = 0.80 (100:1 petroleum ether/EtOAc).

^{1}H NMR (400 MHz, $CDCl_3$, ppm) *δ* 8.03 – 8.01 (m, 2H), 7.74 – 7.71 (m, 2H), 7.47 – 7.41 (m, 4H), 7.39 – 7.35 (m, 1H), 7.06 – 7.01 (m, 1H); ^{13}C NMR (100 MHz, $CDCl_3$, ppm) *δ* 168.0, 163.2 (d, *J* = 245.1 Hz), 154.9, 136.6 (d, *J* = 8.2 Hz), 133.5, 130.2 (d, *J* = 8.2 Hz), 130.1, 128.9, 126.6, 121.9 (d, *J* = 2.8 Hz), 114.9 (d, *J* = 21.3 Hz), 113.5, 113.4 (d, *J* = 23.0 Hz); ^{19}F NMR (376 MHz, $CDCl_3$, ppm) *δ* -113.0; HRMS (ESI) m/z calcd. for $C_{15}H_{11}FNS^+$ $(M+H)^+$ 256.0591, found 256.0590.

4-(3-Chlorophenyl)-2-phenylthiazole (3as)

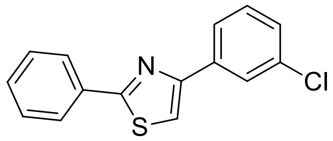

The reaction was conducted with 3'-chloroacetonenone (**2s**, 26.0 μL, 0.2 mmol), benzylamine (**2a**, 44.0 μL, 0.4 mmol), isonicotinic acid (24.6 mg, 0.2 mmol) and sulfur powder (12.8 mg, 0.4 mmol). The residue was purified by column chromatography on silica gel (petroleum ether/EtOAc = 400:1) to yield the desired product **3as** as white solid (35.2 mg, 65% yield), mp 80-81 °C. R_f = 0.80 (100:1 petroleum ether/EtOAc).

^{1}H NMR (400 MHz, $CDCl_3$, ppm) *δ* 8.03 – 8.00 (m, 3H), 7.83 (d, *J* = 7.4 Hz, 1H), 7.48 – 7.41 (m, 4H), 7.37 – 7.29 (m, 2H); ^{13}C NMR (100 MHz, $CDCl_3$, ppm) *δ* 168.1, 154.7, 136.1, 134.7, 133.5, 130.2, 129.9, 128.9, 128.1, 126.6, 124.4, 113.5; HRMS (ESI) m/z calcd. for $C_{15}H_{11}ClNS^+$ $(M+H)^+$ 272.0295, found 272.0300.

4-(3-Bromophenyl)-2-phenylthiazole (3at, CAS: 2362-60-9)

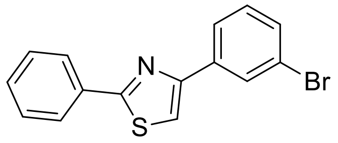

The reaction was conducted with 3'-bromoacetophenone (**3t**, 27.0 μL, 0.2 mmol), benzylamine (**2a**, 44.0 μL, 0.4 mmol), isonicotinic acid (24.6 mg, 0.2 mmol) and sulfur powder (12.8 mg, 0.4 mmol). The residue was purified by column chromatography on silica gel (petroleum ether/EtOAc = 300:1) to yield the desired product **3at** as white solid (42.2 mg, 67% yield), mp 97-98 °C. R_f = 0.70 (100:1 petroleum ether/EtOAc).

^{1}H NMR (400 MHz, $CDCl_3$, ppm) δ 8.15 (s, 1H), 8.02 – 7.99 (m, 2H), 7.86 (d, J = 7.8 Hz, 1H), 7.45 – 7.42 (m, 5H), 7.27 (t, J = 7.9 Hz, 1H); ^{13}C NMR (100 MHz, $CDCl_3$, ppm) δ 168.1, 154.5, 136.3, 133.4, 131.0, 130.2, 129.4, 128.9, 126.5, 124.8, 122.9, 113.5; HRMS (ESI) m/z calcd. for $C_{15}H_{11}BrNS^+$ $(M+H)^+$ 315.9790, found 315.9798.

4-(3-Nitrophenyl)-2-phenylthiazole (3au, CAS: 2521-26-8)

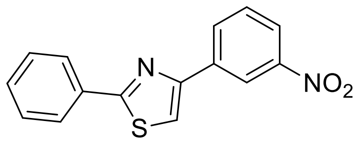

The reaction was conducted with 3'-nitroacetophenone (**2u**, 33.5 mg, 0.2 mmol), benzylamine (**2a**, 44.0 μL, 0.4 mmol), isonicotinic acid (24.6 mg, 0.2 mmol) and sulfur powder (12.8 mg, 0.4 mmol). The residue was purified by column chromatography on silica gel (petroleum ether/EtOAc = 10:1) to yield the desired product **3au** as yellow solid (33.8 mg, 60% yield), mp 143-145 °C. R_f = 0.60 (5:1 petroleum ether/EtOAc).

^{1}H NMR (400 MHz, $CDCl_3$, ppm) δ 8.80 (s, 1H), 8.30 (d, J = 7.8 Hz, 1H), 8.18 – 8.16 (m, 1H), 8.04 – 8.01 (m, 2H), 7.60 – 7.56 (m, 2H), 7.49 – 7.46 (m, 3H); ^{13}C NMR (100 MHz, $CDCl_3$, ppm) δ 168.6, 153.6, 148.7, 136.0, 133.2, 132.1, 130.4, 129.6, 129.0, 126.6, 122.6, 121.2, 114.5; HRMS (ESI) m/z calcd for $C_{15}H_{11}N_2O_2S^+$ $(M+H)^+$ 283.0536, found 283.0541.

4-(2-Fluorophenyl)-2-phenylthiazole (3av)

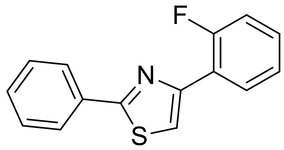

The reaction was conducted with 2'-fluoroacetophenone (**2v**, 25.0 μL, 0.2 mmol), benzylamine (**2a**, 44.0 μL, 0.4 mmol), isonicotinic acid (24.6 mg, 0.2 mmol) and sulfur powder (12.8 mg, 0.4 mmol). The residue was purified by column chromatography on silica gel (petroleum ether/EtOAc = 400:1) to yield the desired product **3av** as white solid (28.1 mg, 55% yield), mp 67-68 ^{o}C. R_f = 0.60 (100:1 petroleum ether/EtOAc).

^{1}H NMR (400 MHz, $CDCl_3$, ppm) δ 8.40 – 8.36 (m, 1H), 8.05 – 8.02 (m, 2H), 7.75 (d, J = 2.2 Hz, 1H), 7.48 – 7.42 (m, 3H), 7.30 – 7.23 (m, 2H), 7.17 – 7.12 (m, 1H)); ^{13}C NMR (100 MHz, $CDCl_3$, ppm) δ 166.7, 160.3 (d, J = 249.8 Hz), 149.7, 133.5, 130.2 (d, J = 3.2 Hz), 130.1, 129.2 (d, J = 8.7 Hz), 128.9, 126.6, 124.4 (d, J = 3.4 Hz), 122.2 (d, J = 11.3 Hz), 117.4 (d, J = 15.1 Hz), 115.9 (d, J = 22.5 Hz); ^{19}F NMR (376 MHz, $CDCl_3$, ppm) δ –114.6; HRMS (ESI) m/z calcd. for $C_{15}H_{11}FNS^+$ $(M+H)^+$ 256.0591, found 256.0596.

4-(2-Chlorophenyl)-2-phenylthiazole (3aw)

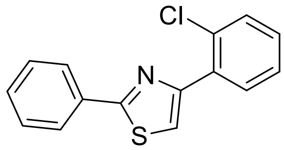

The reaction was conducted with 2'-chloroacetophenone (**2w**, 26.0 μL, 0.2 mmol), benzylamine (**2a**, 44.0 μL, 0.4 mmol), isonicotinic acid (24.6 mg, 0.2 mmol) and sulfur powder (12.8 mg, 0.4 mmol). The residue was purified by column chromatography on silica gel (petroleum ether/EtOAc = 400 :1) to yield the desired product **3aw** as white solid (28.7 mg, 53% yield), mp 71-72 ^{o}C. R_f = 0.70 (100:1 petroleum ether/EtOAc).

^{1}H NMR (400 MHz, $CDCl_3$, ppm) δ 8.08 (dd, J = 7.8, 1.7 Hz, 1H), 8.03 – 8.01 (m, 2H), 7.83 (s, 1H), 7.49 – 7.42 (m, 4H), 7.38 – 7.34 (m, 1H), 7.30 – 7.26 (m, 1H); ^{13}C NMR (100 MHz, $CDCl_3$, ppm) δ 166.7, 152.5, 133.5, 133.1, 132.0, 131.6, 130.4, 130.1, 129.0, 128.9, 126.9, 126.6, 117.9; HRMS (ESI) m/z calcd. for $C_{15}H_{11}ClNS^+$ $(M+H)^+$ 272.0295, found 272.0300.

4-(2,5-Dichlorophenyl)-2-phenylthiazole (3ax)

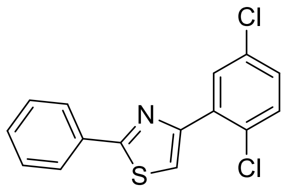

The reaction was conducted with 2',5'-dichloroacetophenone (**2x**, 29.0 μL, 0.2 mmol), benzylamine (**2a**, 44.0 μL, 0.4 mmol), isonicotinic acid (24.6 mg, 0.2 mmol) and sulfur powder (12.8 mg, 0.4 mmol). The residue was purified by column chromatography on silica gel (petroleum ether/EtOAc = 400 :1) to yield the desired product **3ax** as white solid (31.1 mg, 51% yield), mp 92-93 °C. R_f = 0.70 (100:1 petroleum ether/EtOAc).

^{1}H NMR (400 MHz, $CDCl_3$, ppm) δ 8.15 (d, J = 2.5 Hz, 1H), 8.03 – 8.01 (m, 2H), 7.91 (s, 1H), 7.48 – 7.44 (m, 3H), 7.40 (d, J = 8.5 Hz, 1H), 7.26 – 7.23 (m, 1H); ^{13}C NMR (100 MHz, $CDCl_3$, ppm) δ 167.0, 151.1, 134.3, 133.2, 132.9, 131.6, 131.4, 130.3, 130.0, 129.0, 128.8, 126.6, 118.7; HRMS (ESI) m/z calcd. for $C_{15}H_{10}Cl_2NS^+$ $(M+H)^+$ 305.9906, found 305.9910.

4-(3,4-Dichlorophenyl)-2-phenylthiazole (3ay)

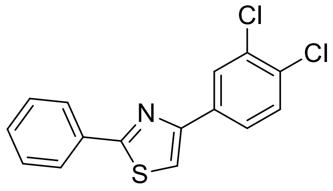

The reaction was conducted with 3',4'-dichloroacetophenone (**2y**, 37.8 mg, 0.2 mmol), benzylamine (**2a**, 44.0 μL, 0.4 mmol), isonicotinic acid (24.6 mg, 0.2 mmol) and sulfur powder (12.8 mg, 0.4 mmol). The residue was purified by column chromatography on silica gel (petroleum ether/EtOAc = 400 :1) to yield the desired product **3ay** as white solid (43.1 mg, 72% yield), mp 121-122 °C. R_f = 0.70 (100:1 petroleum ether/EtOAc).

^{1}H NMR (400 MHz, $CDCl_3$, ppm) δ 8.09 – 8.08 (m, 1H), 8.01 – 7.99 (m, 2H), 7.75 (dd, J = 8.4, 2.0 Hz, 1H), 7.47 – 7.43 (m, 5H); ^{13}C NMR (100 MHz, $CDCl_3$, ppm) δ 168.3, 153.7, 134.3, 133.3, 132.9, 131.9, 130.6, 130.3, 128.9, 128.2, 126.6, 125.4, 113.7; HRMS (ESI) m/z calcd. for $C_{15}H_{10}Cl_2NS^+$ $(M+H)^+$ 305.9906, found 305.9902.

9-Phenylnaphtho[1',2':4,5]thieno[3,2-d]thiazole (3az)

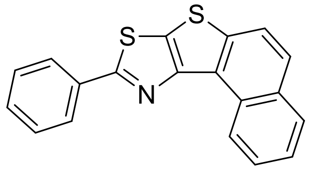

The reaction was conducted with 1'-acetonaphthone (**2z**, 33.0 μL, 0.2 mmol), benzylamine (**2a**, 44.0 μL, 0.4 mmol), isonicotinic acid (24.6 mg, 0.2 mmol) and sulfur powder (12.8 mg, 0.4 mmol). The residue was purified by column chromatography on silica gel (petroleum ether/EtOAc = 400 :1) to yield the desired product **3az** as white solid (25.4 mg, 40% yield), mp 158-160 °C. R_f = 0.70 (100:1 petroleum ether/EtOAc).

^{1}H NMR (400 MHz, $CDCl_3$, ppm) δ 9.61 (d, J = 8.2 Hz, 1H), 8.13 – 8.11 (m, 2H), 7.95 (d, J = 8.1 Hz, 1H), 7.81 – 7.71 (m, 3H), 7.60 – 7.56 (m, 1H), 7.54 – 7.43(m, 3H); ^{13}C NMR (100 MHz, $CDCl_3$, ppm) δ 170.4, 157.3, 140.2, 134.1, 131.4, 130.7, 130.3, 129.0, 128.7, 128.0, 126.7, 126.7, 125.8, 125.7, 124.9, 121.1; HRMS (ESI) m/z calcd. for $C_{19}H_{12}NS_2^+$ $(M+H)^+$ 318.0406, found 318.0408.

8-Phenylnaphtho[2',1':4,5]thieno[3,2-*d*]thiazole (3aa')[6]

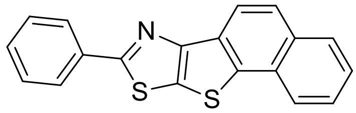

The reaction was conducted with 2'-acetonaphthone (**2a'**, 34.0 mg, 0.2 mmol), benzylamine (**2a**, 44.0 μL, 0.4 mmol), isonicotinic acid (24.6 mg, 0.2 mmol) and sulfur powder (12.8 mg, 0.4 mmol). The residue was purified by column chromatography on silica gel (petroleum ether/EtOAc = 400 :1) to yield the desired product **3aa'** as white solid (28.5 mg, 45% yield), mp 192-193 °C. R_f = 0.70 (100:1 petroleum ether/EtOAc).

^{1}H NMR (400 MHz, $CDCl_3$, ppm) δ 8.27 (d, J = 8.6 Hz, 1H), 8.04 (d, J = 7.6 Hz, 3H), 7.94 (d, J = 7.9 Hz, 1H), 7.85 (d, J = 8.6 Hz, 1H), 7.61 – 7.40 (m, 5H); ^{13}C NMR (100 MHz, $CDCl_3$, ppm) δ 170.8, 157.4, 140.2, 134.0, 131.4, 130.3, 129.8, 129.2, 129.0, 128.2, 126.8, 126.6, 126.1, 125.9, 122.6, 120.2.

11-Phenylphenanthro[9',10':4,5]thieno[3,2-d]thiazole (3ab')

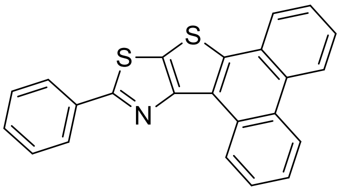

The reaction was conducted with 9-acetylphenanthrene (**2b'**, 45.0 mg, 0.2 mmol), benzylamine (**2a**, 44.0 μL, 0.4 mmol), isonicotinic acid (24.6 mg, 0.2 mmol) and sulfur powder (12.8 mg, 0.4 mmol). The residue was purified by column chromat ography on silica gel (petroleum ether/EtOAc = 400 :1) to yield the desired product **3ab'** as white solid (22.7 mg, 31% yield), mp 183-184 °C. R_f = 0.70 (100:1 petroleum ether/EtOAc).

^{1}H NMR (400 MHz, $CDCl_3$, ppm) δ 9.74 (d, J = 8.1 Hz, 1H), 8.72 – 8.70 (m, 2H), 8.16 – 8.14 (m, 2H), 8.09 – 8.06 (m, 1H), 7.80 (t, J = 7.5 Hz, 1H), 7.73 – 7.69 (m, 1H), 7.66 – 7.61 (m, 2H), 7.55 – 7.47 (m, 3H); ^{13}C NMR (100 MHz, $CDCl_3$, ppm) δ 170.4, 158.4, 139.4, 134.1, 130.3, 130.0, 129.1, 128.9, 128.8, 128.4, 128.2, 127.4, 127.2, 126.7, 126.6, 126.5, 125.6, 125.1, 123.7, 123.1, 122.9; HRMS (ESI) m/z calcd. for $C_{23}H_{14}NS_2^+$ $(M+H)^+$ 368.0562, found 368.0567.

2-Phenylbenzo[4',5']thieno[2',3':4,5]thieno[3,2-*d*]thiazole (3ac')[6]

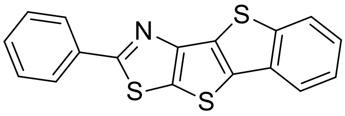

The reaction was conducted with 1-(benzo[*b*]thiophen-2-yl)ethanone (**2c'**, 35.4 mg, 0.2 mmol), benzylamine (**2a**, 44.0 μL, 0.4 mmol), isonicotinic acid (24.6 mg, 0.2 mmol) and sulfur powder (12.8 mg, 0.4 mmol). The residue was purified by column chromat ography on silica gel (petroleum ether/EtOAc = 50 :1) to yield the desired product **3ac'** as white solid (19.4 mg, 30% yield), mp 210-211 °C. R_f = 0.70 (100:1 petroleum ether/EtOAc).

^{1}H NMR (400 MHz, $CDCl_3$, ppm) δ 8.00 – 7.94 (m, 2H), 7.89 (d, J = 7.9 Hz, 1H), 7.77 (d, J = 8.0 Hz, 1H), 7.49 – 7.44 (m, 4H), 7.41 (t, J = 7.5 Hz, 1H); ^{13}C NMR (100 MHz, $CDCl_3$, ppm) δ 166.9, 151.6, 139.2, 137.0, 134.5, 132.7, 130.7, 129.1, 126.5, 125.6, 125.1, 123.0, 122.9, 120.2, 119.2.

4-Phenyl-2-(*p*-tolyl)thiazole (3ba, CAS: 2227-61-4)[3]

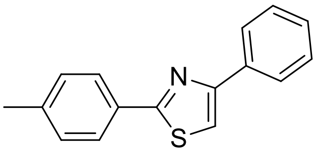

The reaction was conducted with acetophenone (**1a**, 24.0 μL, 0.2 mmol), 4-mthylbenzylamine (**2b**, 51.0 μL, 0.4 mmol), isonicotinic acid (24.6 mg, 0.2 mmol) and sulfur powder (12.8 mg, 0.4 mmol). The residue was purified by column chromatography on silica gel (petroleum ether/EtOAc = 400:1) to yield the desired product **3ba** as white solid (31.6 mg, 63% yield), mp 126-127 °C. R_f = 0.70 (100:1 petroleum ether/EtOAc).

^{1}H NMR (400 MHz, $CDCl_3$, ppm) δ 7.99 (d, J = 7.4 Hz, 2H), 7.93 (d, J = 8.1 Hz, 2H), 7.45 – 7.42 (m, 3H), 7.34 (t, J = 7.4 Hz, 1H), 7.25 (d, J = 8.0 Hz, 2H), 2.39 (s, 3H); ^{13}C NMR (100 MHz, $CDCl_3$, ppm) δ 168.0, 156.1, 140.2, 134.6, 131.1, 129.5, 128.7, 128.1, 126.5, 126.4, 112.1, 21.4.

2-(4-Fluorophenyl)-4-phenylthiazole (3ca, CAS: 1095792-53-2)[7]

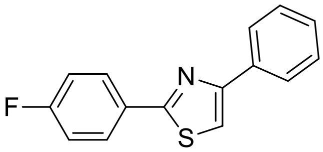

The reaction was conducted with acetophenone (**1a**, 24.0 μL, 0.2 mmol), 4-fluorobenzylamine (**2c**, 46.0 μL, 0.4 mmol), isonicotinic acid (24.6 mg, 0.2 mmol) and sulfur powder (12.8 mg, 0.4 mmol). The residue was purified by column chromatography on silica gel (petroleum ether/EtOAc = 300:1) to yield the desired product **3ca** as white solid (30.1 mg, 59% yield), mp 117-118 °C. R_f = 0.60 (100:1 petroleum ether/EtOAc).

^{1}H NMR (400 MHz, $CDCl_3$, ppm) δ 8.05 – 7.99 (m, 4H), 7.48 – 7.44 (m, 3H), 7.39 – 7.35 (m, 1H), 7.18 – 7.14 (m, 2H); ^{13}C NMR (100 MHz, $CDCl_3$, ppm) δ 166.6, 163.8 (d, J = 250.3 Hz), 156.3, 134.3, 130.1 (d, J = 3.1 Hz), 128.7, 128.5 (d, J = 8.5 Hz), 128.2, 126.4, 116.1, 115.9 (d, J = 22.0 Hz); ^{19}F NMR (376 MHz, $CDCl_3$, ppm) δ –110.5.

2-(4-Chlorophenyl)-4-phenylthiazole (3da, CAS: 2227-72-7)[3]

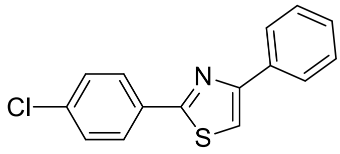

The reaction was conducted with acetophenone (**1a**, 24.0 μL, 0.2 mmol), 4-chlorobenzylamine (**2d**, 50.0 μL, 0.4 mmol), isonicotinic acid (24.6 mg, 0.2 mmol) and sulfur powder (12.8 mg, 0.4 mmol). The residue was purified by column chromatography on silica gel (petroleum ether/EtOAc = 300:1) to yield the desired product **3da** as white solid (33.1 mg, 61% yield), mp 104-105 °C. R_f = 0.65 (100:1 petroleum ether/EtOAc).

^{1}H NMR (400 MHz, $CDCl_3$, ppm) δ 7.97 – 7.93 (m, 4H), 7.50 – 7.38 (m, 5H), 7.36 – 7.32 (m, 1H); ^{13}C NMR (100 MHz, $CDCl_3$, ppm) δ 166.4, 156.3, 135.9, 134.2, 132.1, 129.1, 128.7, 128.2, 127.7, 126.4, 112.8.

2-(4-Bromophenyl)-4-phenylthiazole (3ea, CAS: 859471-47-9)[8]

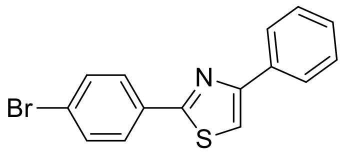

The reaction was conducted with acetophenone (**1a**, 24.0 μL, 0.2 mmol), 4-bromobenzylamine (**2e**, 52.0 μL, 0.4 mmol), isonicotinic acid (24.6 mg, 0.2 mmol) and sulfur powder (12.8 mg, 0.4 mmol). The residue was purified by column chromatography on silica gel (petroleum ether/EtOAc = 300:1) to yield the desired product **3ea** as white solid (44.1 mg, 70% yield), mp 104-105 °C. R_f = 0.60 (100:1 petroleum ether/EtOAc).

^{1}H NMR (400 MHz, $CDCl_3$, ppm) δ 7.97 (d, J = 7.3 Hz, 2H), 7.89 (d, J = 8.5 Hz, 2H), 7.57 (d, J = 8.4 Hz, 2H), 7.49 – 7.41 (m, 3H), 7.35 (t, J = 7.3 Hz, 1H); ^{13}C NMR (100 MHz, $CDCl_3$, ppm) δ 166.5, 156.4, 134.2, 132.6, 132.0, 128.7, 128.3, 127.9, 126.4, 124.2, 112.8.

4-Phenyl-2-(4-(trifluoromethyl)phenyl)thiazole (3fa)[3]

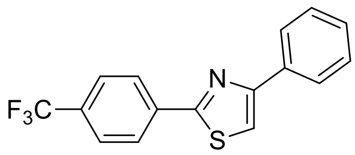

The reaction was conducted with acetophenone (**1a**, 24.0 μL, 0.2 mmol),

4-(trifluoromethyl)benzylamine (**2f**, 57.0 μL, 0.4 mmol), isonicotinic acid (24.6 mg, 0.2 mmol) and sulfur powder (12.8 mg, 0.4 mmol). The residue was purified by column chromatography on silica gel (petroleum ether/EtOAc = 300:1) to yield the desired product **3fa** as white solid (43.3 mg, 71% yield), mp 119-120 °C. R_f = 0.70 (100:1 petroleum ether/EtOAc).

^{1}H NMR (400 MHz, $CDCl_3$, ppm) δ 8.13 (d, J = 8.1 Hz, 2H), 7.99 – 7.97 (m, 2H), 7.69 (d, J = 8.3 Hz, 2H), 7.51 (s, 1H), 7.45 (t, J = 7.5 Hz, 2H), 7.38 – 7.35 (m, 1H); ^{13}C NMR (100 MHz, $CDCl_3$, ppm) δ 165.9, 156.8, 136.8, 134.1, 131.6 (q, J = 32.4 Hz), 128.8, 128.4, 126.7, 126.4, 125.9 (q, J = 3.7 Hz), 123.9 (q, J = 270.6 Hz), 113.62; ^{19}F NMR (376 MHz, $CDCl_3$, ppm) δ –62.7.

4-Phenyl-2-(4-(trifluoromethoxy)phenyl)thiazole (3ga)

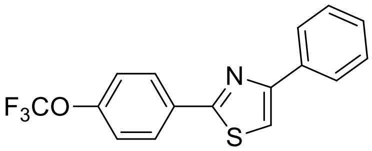

The reaction was conducted with acetophenone (**1a**, 24.0 μL, 0.2 mmol), 4-(trifluoromethoxy)benzylamine (**2g**, 68.0 μL, 0.4 mmol), isonicotinic acid (24.6 mg, 0.2 mmol) and sulfur powder (12.8 mg, 0.4 mmol). The residue was purified by column chromatography on silica gel (petroleum ether/EtOAc = 300:1) to yield the desired product **3ga** as white solid (32.7 mg, 51% yield), mp 100-101 °C. R_f = 0.50 (100:1 petroleum ether/EtOAc).

^{1}H NMR (400 MHz, $CDCl_3$, ppm) δ 8.07 (d, J = 8.6 Hz, 2H), 7.99 (d, J = 7.8 Hz, 2H), 7.49 (s, 1H), 7.45 (t, J = 7.6 Hz, 2H), 7.36 (t, J = 7.3 Hz, 1H), 7.31 (d, J = 8.4 Hz, 2H); ^{13}C NMR (100 MHz, $CDCl_3$, ppm) δ 166.1, 156.5, 150.3, 134.3, 132.4, 128.8, 128.3, 128.1, 126.4, 121.2, 120.4 (q, J = 256.2 Hz), 113.0; ^{19}F NMR (376 MHz, $CDCl_3$, ppm) δ -57.7; HRMS (ESI) m/z calcd. for $C_{16}H_{11}F_3NOS^+$ $(M+H)^+$ 322.0508, found 322.0511.

4-Phenyl-2-(*m*-tolyl)thiazole (3ha, CAS: 2227-70-5)

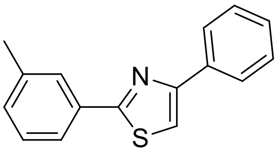

The reaction was conducted with acetophenone (**1a**, 24.0 μL, 0.2 mmol), 3-methylbenzylamine (**2h**, 55 μL, 0.4 mmol), isonicotinic acid (24.6 mg, 0.2 mmol) and sulfur powder (12.8 mg, 0.4

mmol). The residue was purified by column chromatography on silica gel (petroleum ether/EtOAc = 300:1) to yield the desired product **3ha** as white solid (33.1 mg, 66% yield), mp 72-73 °C. R_f = 0.60 (100:1 petroleum ether/EtOAc).

^{1}H NMR (400 MHz, $CDCl_3$, ppm) δ 8.00 – 7.99 (m, 2H), 7.88 (s, 1H), 7.82 (d, J = 7.7 Hz, 1H), 7.46 – 7.42 (m, 3H), 7.36 – 7.32 (m, 2H), 7.25 – 7.23 (m, 1H), 2.43 (s, 3H); ^{13}C NMR (100 MHz, $CDCl_3$, ppm) δ 168.1, 156.2, 138.7, 134.5, 133.6, 130.8, 128.8, 128.7, 128.1, 127.1, 126.4, 123.8, 112.5, 21.4; HRMS (ESI) m/z calcd. for $C_{16}H_{14}NS^+$ $(M+H)^+$ 252.0842, found 252.0845.

2-(3-Fluorophenyl)-4-phenylthiazole (3ia)

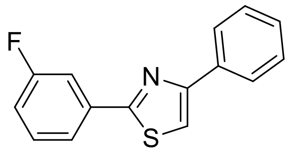

The reaction was conducted with acetophenone (**1a**, 24.0 μL, 0.2 mmol), 3-fluorobenzylamine (**2i**, 46.0 μL, 0.4 mmol), isonicotinic acid (24.6 mg, 0.2 mmol) and sulfur powder (12.8 mg, 0.4 mmol). The residue was purified by column chromatography on silica gel (petroleum ether/EtOAc = 200:1) to yield the desired product **3ia** as light yellow solid (34.7 mg, 68% yield), mp 91-92 °C. R_f = 0.60 (100:1 petroleum ether/EtOAc).

^{1}H NMR (400 MHz, $CDCl_3$, ppm) δ 7.98 – 7.96 (m, 2H), 7.80 – 7.75 (m, 2H), 7.45 – 7.42 (m, 3H), 7.40 – 7.33 (m, 2H), 7.13 – 7.08 (m, 1H); ^{13}C NMR (100 MHz, $CDCl_3$, ppm) δ 166.2, 163.0 (d, J = 246.6 Hz), 156.4, 135.7 (d, J = 8.2 Hz), 134.2, 130.5 (d, J = 8.2 Hz), 128.7, 128.3, 126.4, 122.3 (d, J = 2.8 Hz), 116.8 (d, J = 21.4 Hz), 113.4 (d, J = 23.5 Hz), 113.1; ^{19}F NMR (376 MHz, $CDCl_3$, ppm) δ -112.3; HRMS (ESI) m/z calcd. for $C_{15}H_{11}FNS^+$ $(M+H)^+$ 256.0591, found 256.0594.

4-Phenyl-2-(*o*-tolyl)thiazole (3ja)

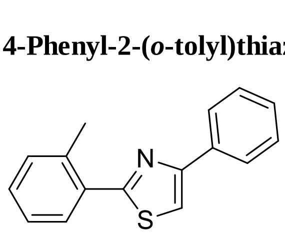

The reaction was conducted with acetophenone (**1a**, 24.0 μL, 0.2 mmol), 2-methylbenzylamine (**2j**, 50.0 μL, 0.4 mmol), isonicotinic acid (24.6 mg, 0.2 mmol) and sulfur powder (12.8 mg, 0.4 mmol). The residue was purified by column chromatography on silica gel (petroleum ether/EtOAc =

300:1) to yield the desired product **3ja** as colourless oily liquid (28.6 mg, 57% yield). R_f = 0.70 (100:1 petroleum ether/EtOAc).

^{1}H NMR (400 MHz, $CDCl_3$, ppm) δ 7.99 (d, J = 7.4 Hz, 2H), 7.78 (d, J = 7.5 Hz, 1H), 7.53 (s, 1H), 7.44 (t, J = 7.6 Hz, 2H), 7.36 – 7.24 (m, 4H), 2.69 (s, 3H); ^{13}C NMR (100 MHz, $CDCl_3$, ppm) δ 167.6, 155.6, 136.7, 134.6, 133.0, 131.6, 129.9, 129.4, 128.7, 128.1, 126.4, 126.1, 113.0, 21.7; HRMS (ESI) m/z calcd. for $C_{16}H_{14}NS^+$ $(M+H)^+$ 252.0842, found 252.0846.

2-(2-Chlorophenyl)-4-phenylthiazole (3ka)

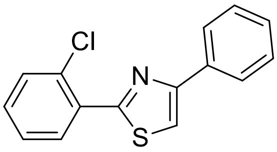

The reaction was conducted with acetophenone (**1a**, 24.0 μL, 0.2 mmol), 2-chlorobenzylamine (**2k**, 52.0 μL, 0.4 mmol), isonicotinic acid (24.6 mg, 0.2 mmol) and sulfur powder (12.8 mg, 0.4 mmol). The residue was purified by column chromatography on silica gel (petroleum ether/EtOAc = 300:1) to yield the desired product **3ka** as white solid (37.9 mg, 70% yield), mp 67-68 °C. R_f = 0.50 (100:1 petroleum ether/EtOAc).

^{1}H NMR (400 MHz, $CDCl_3$, ppm) δ 8.39 (dd, J = 7.7, 1.9 Hz, 1H), 8.01 – 7.99 (m, 2H), 7.61 (s, 1H), 7.49 (dd, J = 7.8, 1.4 Hz, 1H), 7.44 (t, J = 7.6 Hz, 2H), 7.40 – 7.31 (m, 3H); ^{13}C NMR (100 MHz, $CDCl_3$, ppm) δ 163.1, 154.9, 134.3, 131.9, 131.0, 130.6, 130.3, 128.7, 128.2, 127.0, 126.4, 114.6; HRMS (ESI) m/z calcd. for $C_{15}H_{11}ClNS^+$ $(M+H)^+$ 272.0295, found 272.0299.

2-(3-Chloro-4-fluorophenyl)-4-phenylthiazole (3la)

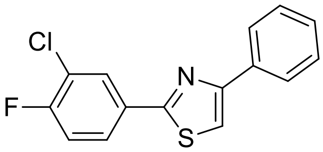

The reaction was conducted with acetophenone (**1a**, 24.0 μL, 0.2 mmol), 3-chloro-4-fluorobenzylamine (**2l**, 64.0 mg, 0.4 mmol), isonicotinic acid (24.6 mg, 0.2 mmol) and sulfur powder (12.8 mg, 0.4 mmol). The residue was purified by column chromatography on silica gel (petroleum ether/EtOAc = 300:1) to yield the desired product **3la** as white solid (29.5 mg, 51% yield), mp 108-109 °C. R_f = 0.50 (100:1 petroleum ether/EtOAc).

^{1}H NMR (400 MHz, $CDCl_3$, ppm) δ 8.12 (dd, J = 7.0, 2.2 Hz, 1H), 7.97 (d, J = 7.3 Hz, 2H), 7.88 – 7.85 (m, 1H), 7.47 – 7.43 (m, 3H), 7.36 (t, J = 7.3 Hz, 1H), 7.21 (t, J = 8.6 Hz, 1H); ^{13}C NMR (100 MHz, $CDCl_3$, ppm) δ 165.1, 159.1 (d, J = 252.7 Hz), 156.5, 134.1, 131.0 (d, J = 3.9 Hz), 128.8, 128.7, 128.4, 126.4, 126.3, 121.9 (d, J = 18.4 Hz), 117.0 (d, J = 21.8 Hz), 113.0; ^{19}F NMR (376 MHz, $CDCl_3$, ppm) δ -113.0; HRMS (ESI) m/z calcd. for $C_{15}H_{10}ClFNS^+$ $(M+H)^+$ 290.0201, found 290.0205.

2-(3, 5-Difluorophenyl)-4-phenylthiazole (3ma)

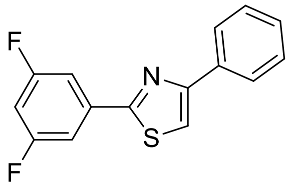

The reaction was conducted with acetophenone (**1a**, 24.0 μL, 0.2 mmol), (3,5-difluorophenyl)methanamine (**2m**, 58.0 mg, 0.4 mmol), isonicotinic acid (24.6 mg, 0.2 mmol) and sulfur powder (12.8 mg, 0.4 mmol). The residue was purified by column chromatography on silica gel (petroleum ether/EtOAc = 300:1) to yield the desired product **3ma** as white solid (36.6 mg, 67% yield), mp 135-136 °C. R_f = 0.50 (100:1 petroleum ether/EtOAc).

^{1}H NMR (400 MHz, $CDCl_3$, ppm) δ 7.96 (d, J = 7.6 Hz, 2H), 7.59 – 7.51 (m, 2H), 7.48 (s,1H), 7.44 (t, J = 7.5 Hz, 2H), 7.35 (t, J = 7.3 Hz, 1H), 6.85 (t, J = 8.7 Hz, 1H); ^{13}C NMR (100 MHz, $CDCl_3$, ppm) δ 164.9 (t, J = 3.6 Hz), 163.2 (dd, J = 249.1, 12.7 Hz), 156.6, 136.6 (t, J = 10.0 Hz), 134.0, 128.8, 128.5, 126.4, 113.5, 109.4 (dd, J = 27.2, 11.7 Hz), 105.1 (t, J = 25.5 Hz); ^{19}F NMR (376 MHz, $CDCl_3$, ppm) δ -108.6; HRMS (ESI) m/z calcd. for $C_{15}H_{10}F_2NS^+$ $(M+H)^+$ 274.0497, found 274.0451.

4-Phenyl-2-(pyridin-4-yl)thiazole (3na, CAS: 106950-18-9)[9]

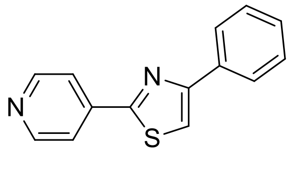

The reaction was conducted with acetophenone (**1a**, 24.0 μL, 0.2 mmol), 4-pyridinemethaneamine (**2n**, 44.0 μL, 0.4 mmol), isonicotinic acid (24.6 mg, 0.2 mmol) and sulfur powder (12.8 mg, 0.4 mmol). The residue was purified by column chromatography on silica gel (petroleum ether/EtOAc

= 15:1) to yield the desired product **3na** as brown solid (22.4 mg, 47% yield), mp 109-110 °C. R_f = 0.50 (5:1 petroleum ether/EtOAc).

^{1}H NMR (400 MHz, $CDCl_3$, ppm) δ 8.72 (d, J = 5.1 Hz, 2H), 7.98 (d, J = 8.2 Hz, 2H), 7.89 (d, J = 4.9 Hz, 2H), 7.58 (s, 1H), 7.46 (t, J = 7.7 Hz, 2H), 7.38 (t, J = 7.3 Hz, 1H); ^{13}C NMR (100 MHz, $CDCl_3$, ppm) δ 164.7, 157.1, 150.5, 140.4, 133.9, 128.8, 128.5, 126.4, 120.3, 114.3.

***N*-(1-Phenylethyl)benzothioamide (7, CAS: 64551-86-6)[10]**

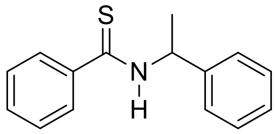

The reaction was conducted with benzaldehyde (4 mmol), 1-phenylethanamine (6 mmol), sulfur powder (8 mmol), and $Na_2S \cdot 9H_2O$ (10 mol%). The residue was purified by column chromatography on silica gel (petroleum ether/ dichloromethane = 1:1) to yield the desired product **7** as yellow solid (0.63 g, 65% yield).

^{1}H NMR (400 MHz, $CDCl_3$, ppm) δ 7.83 (d, J = 6.8 Hz, 1H), 7.72 – 7.60 (m, 2H), 7.45 – 7.24 (m, 8H), 5.97 – 5.78 (m, 1H), 1.64 (s, 3H); ^{13}C NMR (100 MHz, $CDCl_3$, ppm) δ 197.8, 141.7, 141.2, 130.8, 128.6, 128.2, 127.6, 126.5, 126.4, 54.9, 20.1.

(E)-1-Phenyl-*N*-(1-phenylethylidene)methanamine (8, CAS: 14428-98-9)[11]

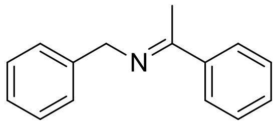

To a solution of benzylamine (6 mmol) in ethanol (25 mL) wasadded the acetophenone (5 mmol) and appropriate molecular sieve, the reaction mixturewas allowed to reflux for 4 h using Dean–Stark apparatus. The progress of the reaction was checked by TLC. Thesolvent was removed in vacuo and the residue was recrystallized with ethanol to yield the desired product **8** as white solid (0.71 g, 68% yield).

^{1}H NMR (400 MHz, CD_3OD-d_4, ppm) δ 7.70 (d, J = 8.1 Hz, 2H), 7.58 – 7.32 (m, 6H), 7.23 (d, J = 8.0 Hz, 2H), 4.09 (s, 2H), 2.36 (s, 3H).

(E)-*N*-(1-Phenylethyl)benzothioamide (9, CAS: 98393-39-6)[12]

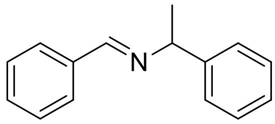

To a solution of 1-phenylethanamine (6 mmol) in ethanol (25 mL) wasadded the benzaldehyde (5 mmol) and appropriate molecular sieve, the reaction mixturewas allowed to reflux for 4 h using Dean–Stark apparatus. The progress of the reaction was checked by TLC. Thesolvent was removed in vacuo and the residue was recrystallized with ethanol to yield the desired product **9** as white solid (0.65 g, 62% yield).

^{1}H NMR (400 MHz, $CDCl_3$, ppm) δ 8.54 (s, 1H), 7.68 (s, 2H), 7.44 – 6.91 (m, 8H), 4.33 – 3.91 (m, 1H), 1.50 (d, J = 4.9 Hz, 3H).

5. References

[1] M. Narender, M. S. Reddy, R. Sridhar, Y. V. D. Nageswar and K. Rama Rao, *Tetrahedron Letters*, 2005, **46**, 5953.

[2] T. Yamamoto and H. Togo, *Eur. J. Org. Chem.*, 2018, 4187.

[3] S. Tani, T. N. Uehara, J. Yamaguchia and K. Itami, *Chem. Sci.*, 2014, **5**, 123.

[4] T. Rozsa, M. Duma, L.Vlase, I. Ionuţ, A. Pîrnău, B.Tiperciuc and O. Oniga, *J. Heterocyclic Chem.*, 2015, **52**, 999.

[5] M. Ueno and H. Togo, *Synthesis*, 2004, 2673.

[6] H. W. Huang, Z. H. Xu, X. C. Ji, B. Li and G.-J. Deng, *Org. Lett.*, 2018, **20**, 4917.

[7] S. K. Kim, J.-H. Kim, Y. C. Park, J. W. Kim and E. K. Yum, *Tetrahedron*, 2013, **69**, 10990.

[8] B. L. C. King and F. M. Miller, *J. Am. Chem. Soc.*, 1948, **71**, 367.

[9] D. Kumar, N. Maruthi Kumar, G. Patel, S. Gupta and R. S. Varma, *Tetrahedron Letters*, 2011, **52**, 1983.

[10] K. Okamoto, T. Yamamoto and T. Kanbara, *Synlett*, 2007, 2687.

[11] R. Sarma and D. Prajapati, *Chem. Commun.*, 2011, **47**, 9525.

[12] Alison E. Wendlandt and Shannon S. Stahl, *Org. Lett.*, 2012, **14**, 2850

6. Copies of ^{1}H, ^{13}C and ^{19}F NMR spectra of all products

^{1}H and ^{13}C NMR spectra of **3aa**

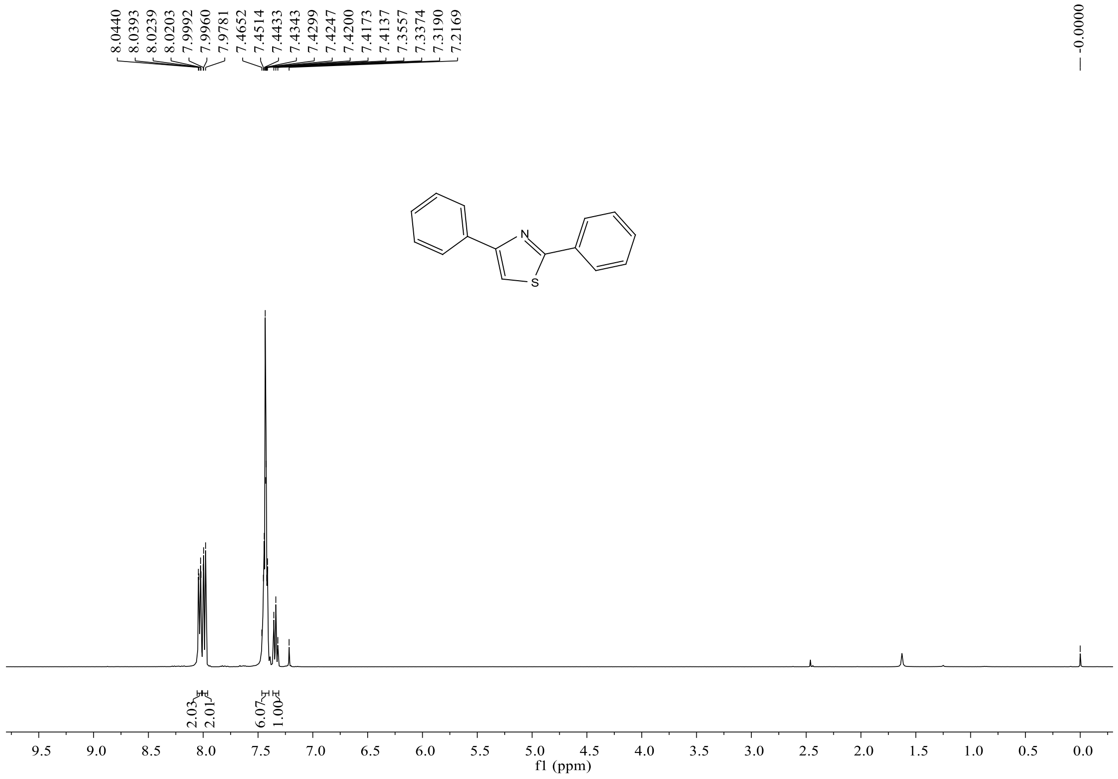

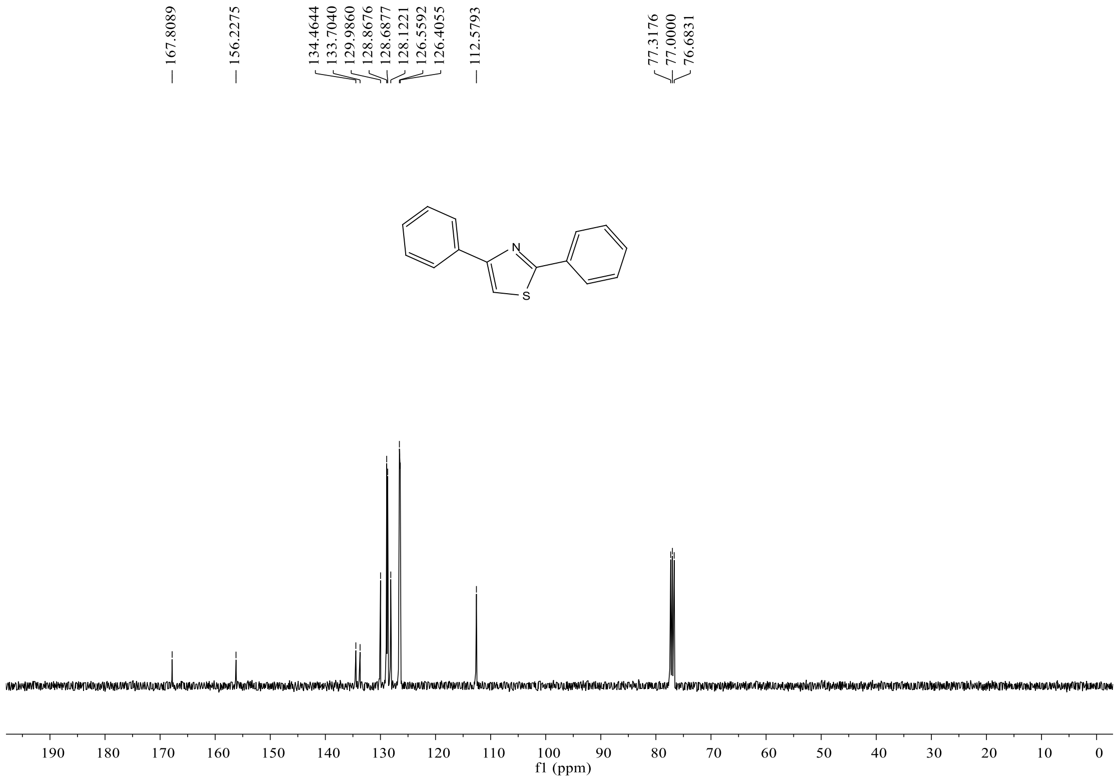

^{1}H and ^{13}C NMR spectra of **3ab**

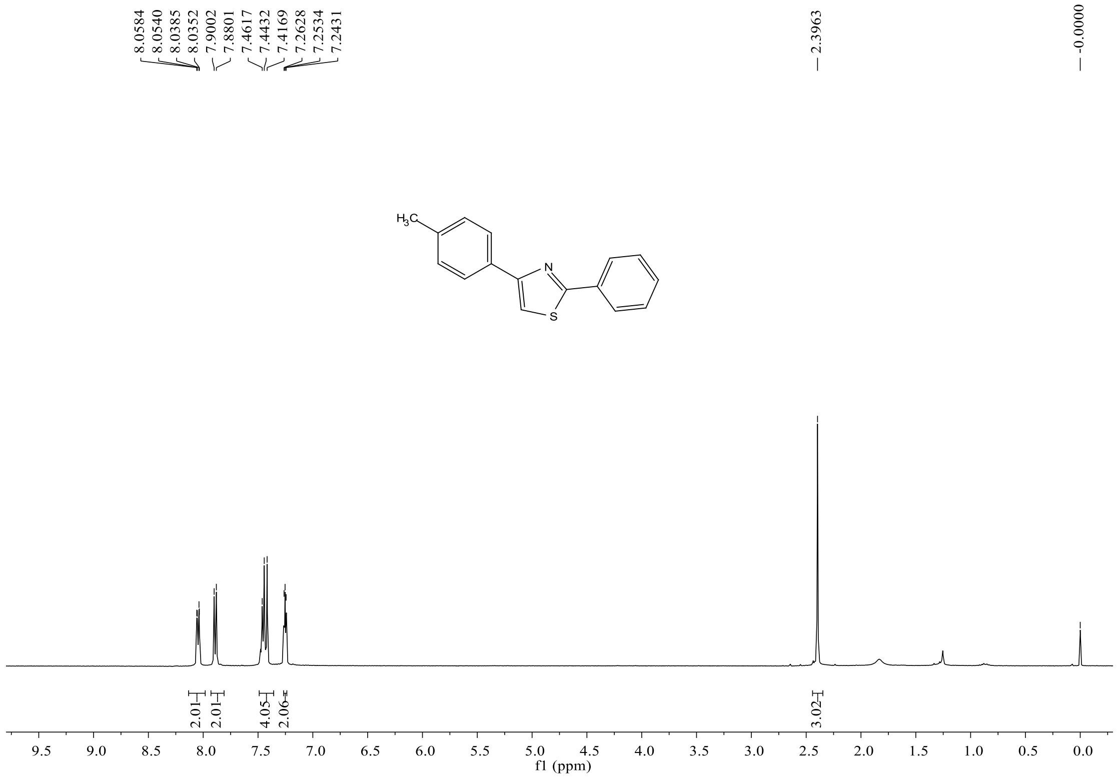

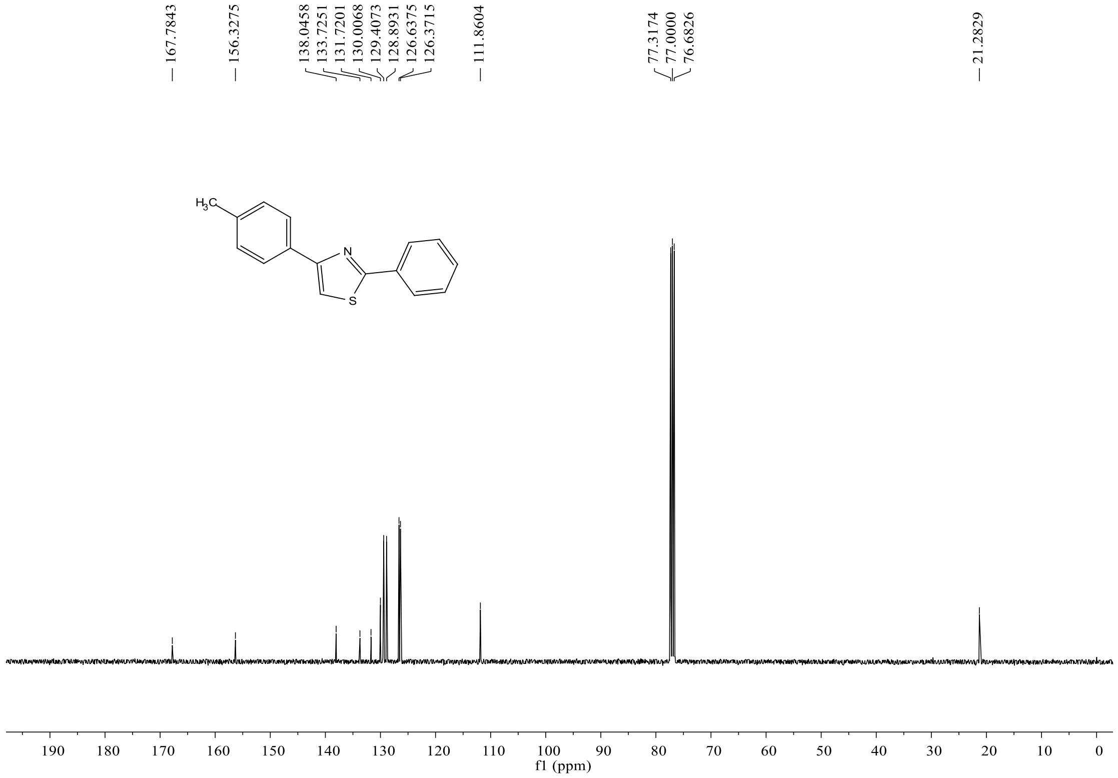

^{1}H and ^{13}C NMR spectra of **3ac**

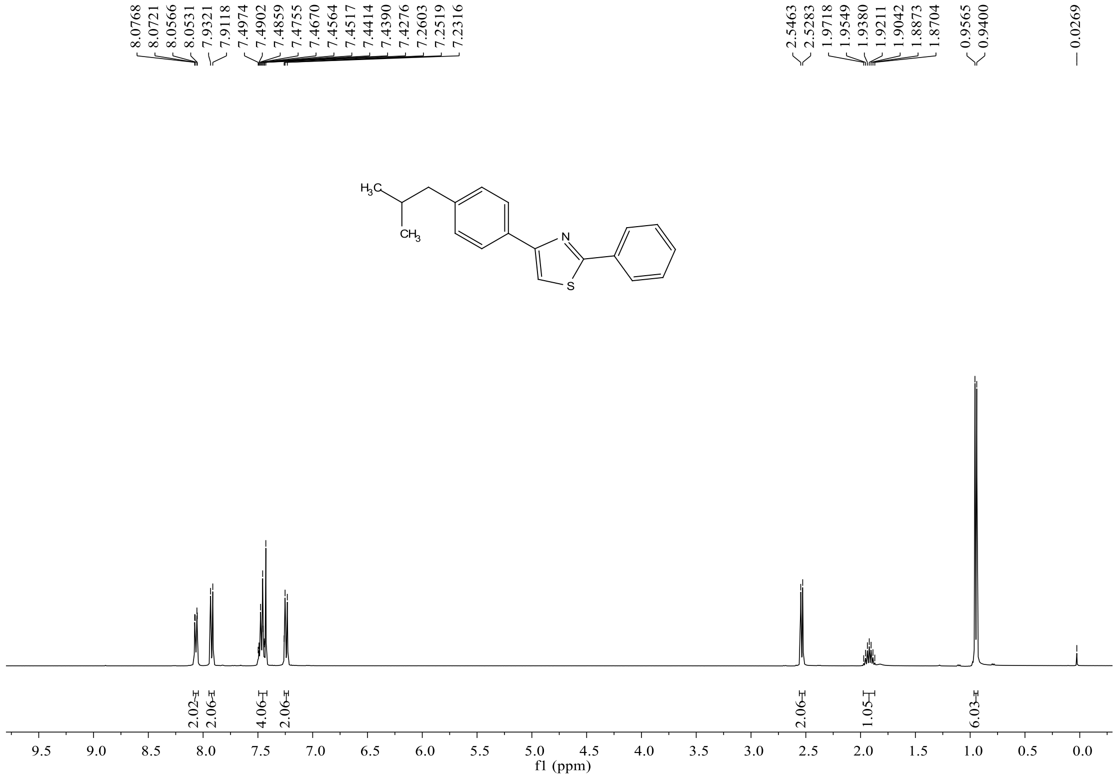

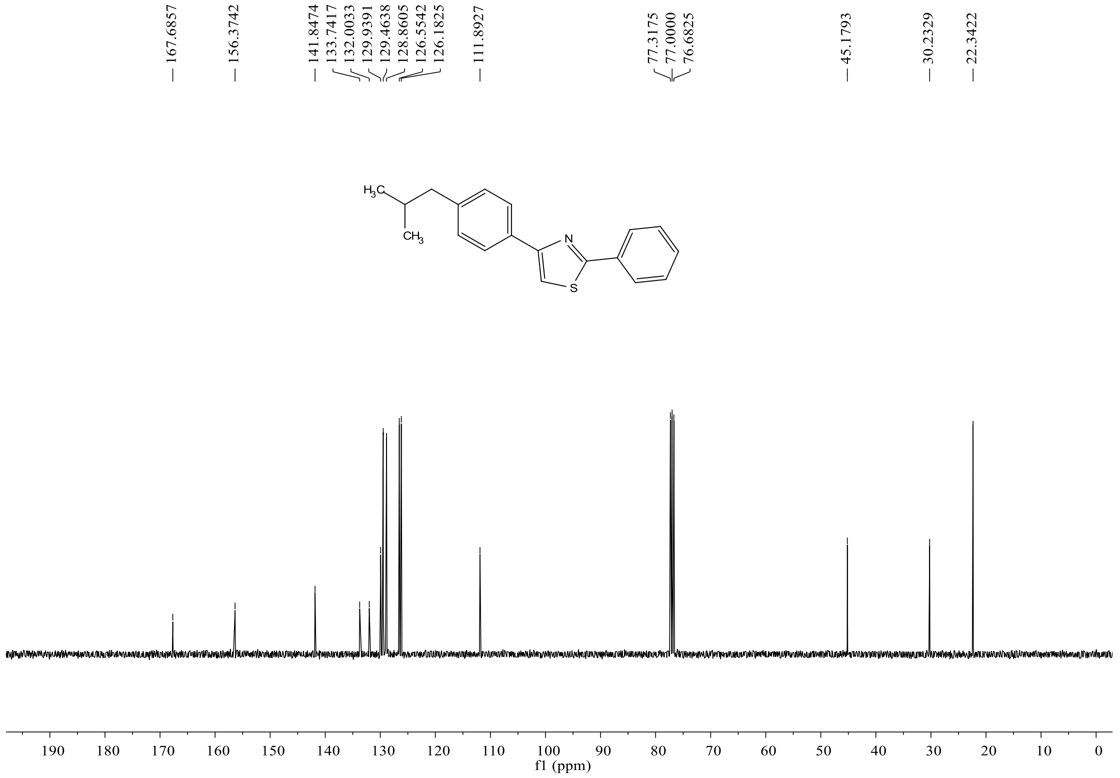

^{1}H and ^{13}C NMR spectra of **3ad**

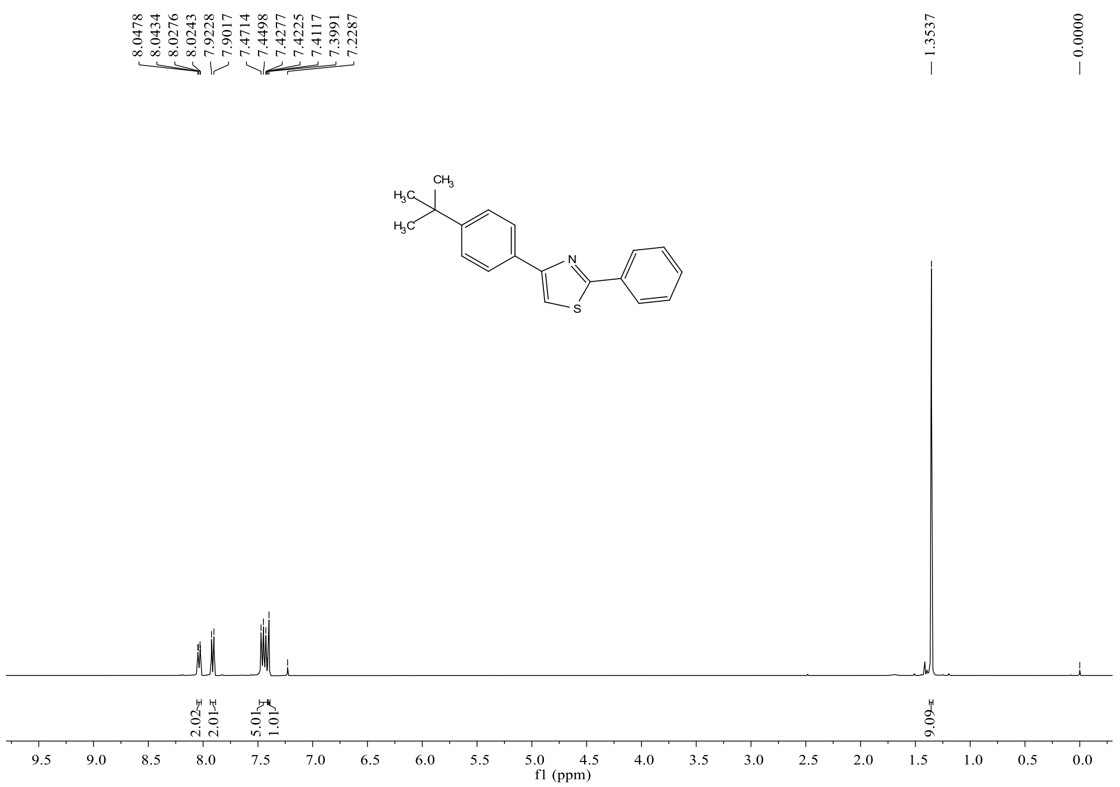

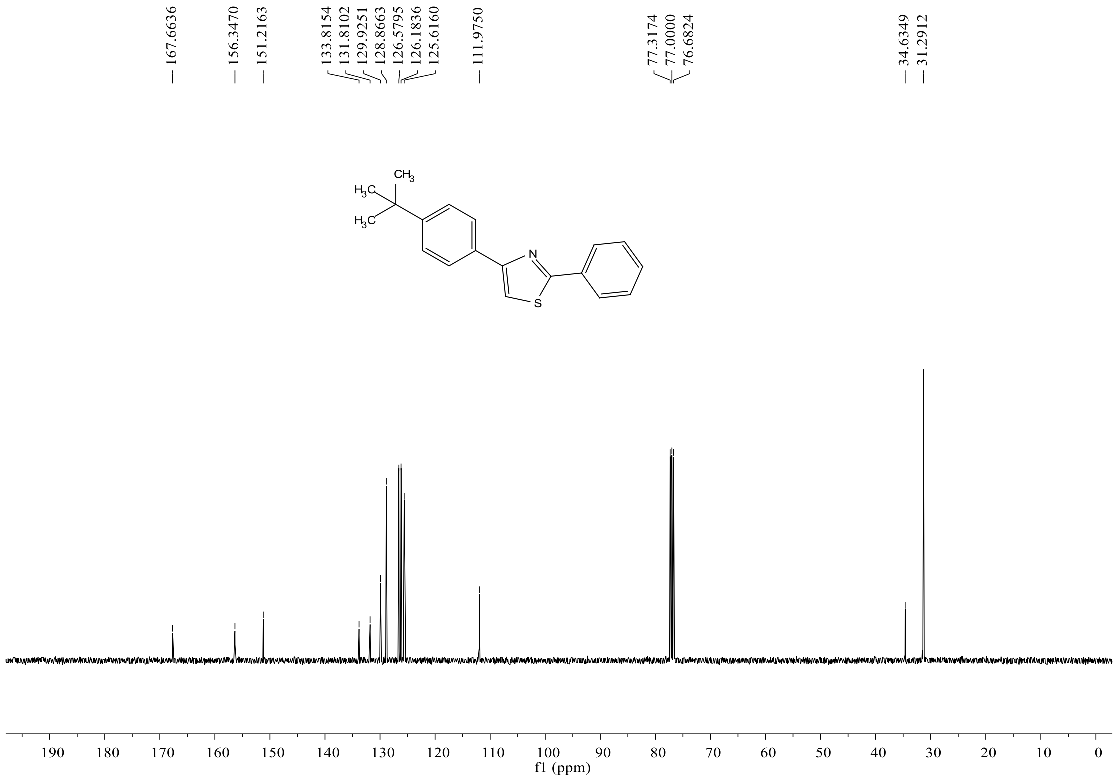

^{1}H and ^{13}C NMR spectra of **3ae**

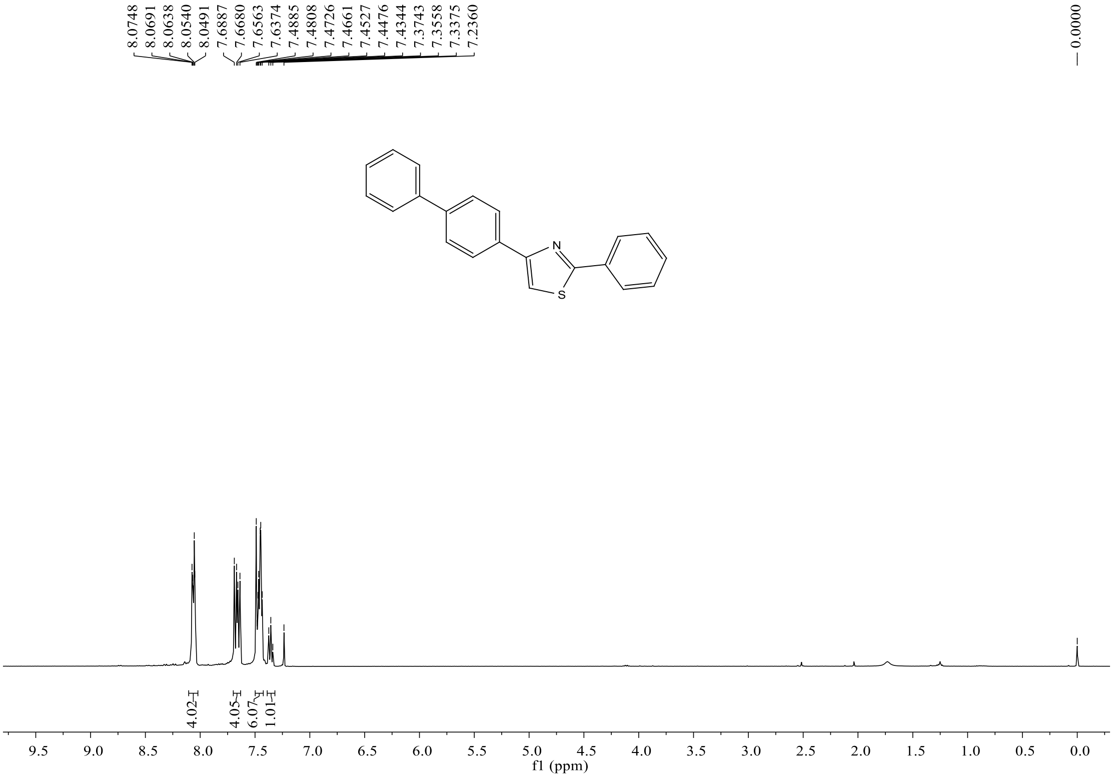

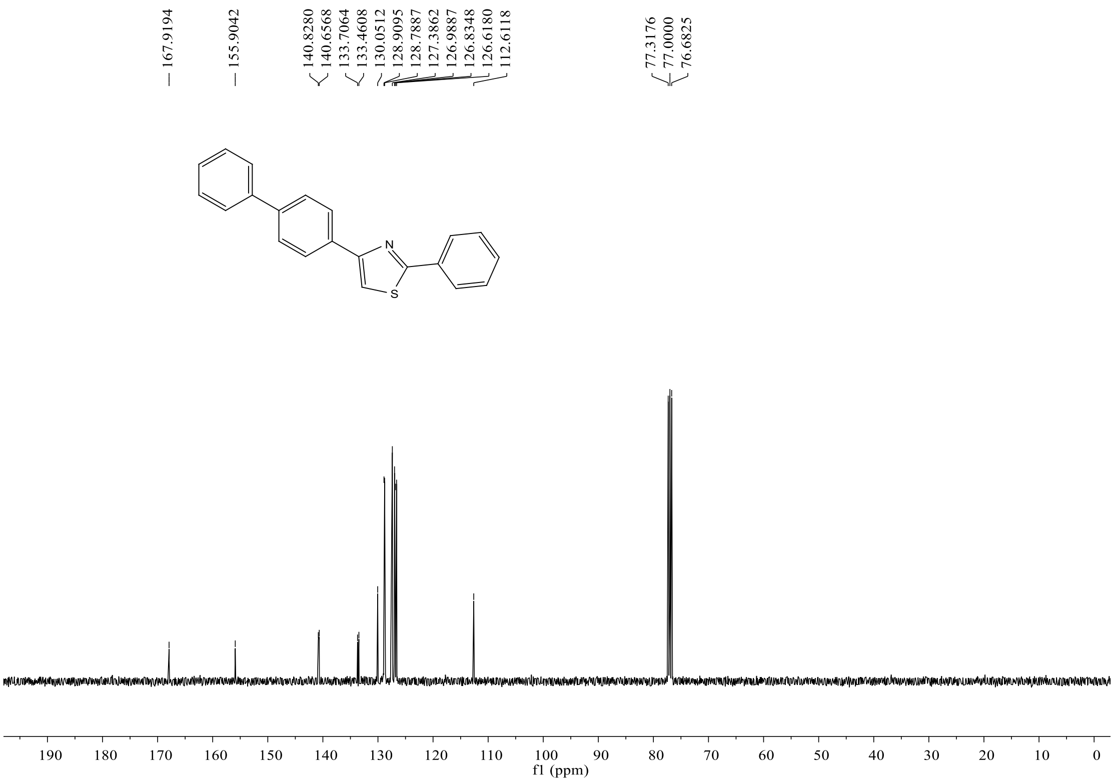

^{1}H and ^{13}C NMR spectra of **3af**

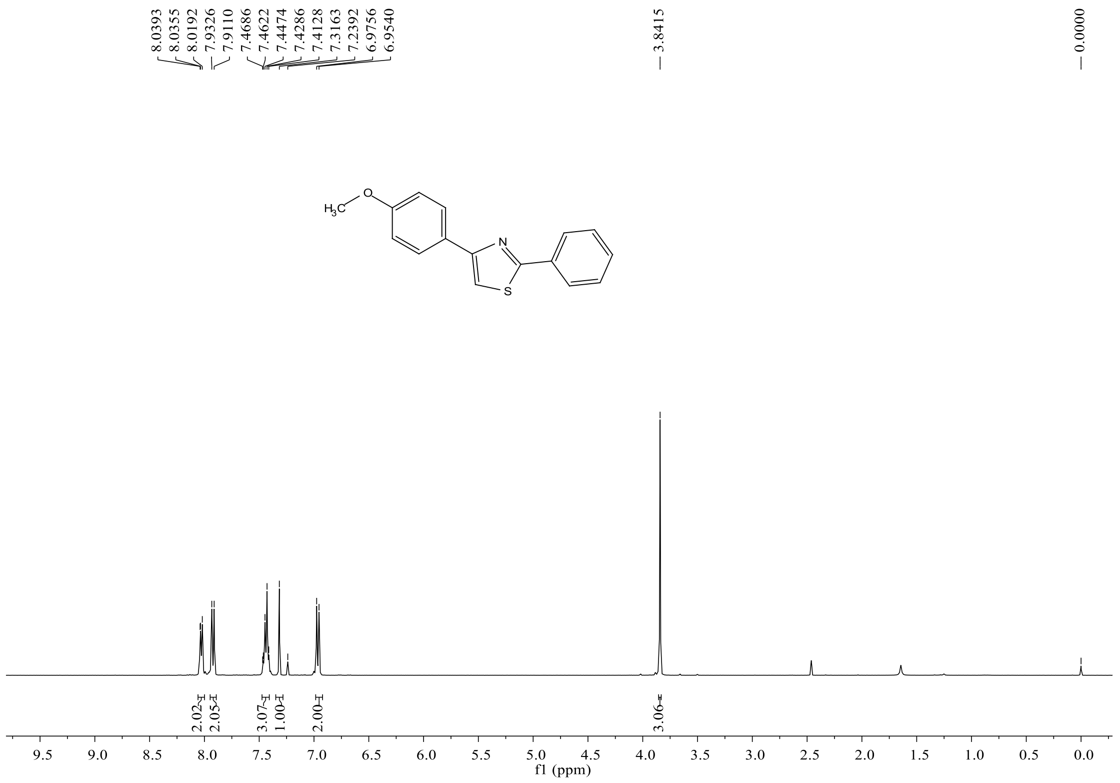

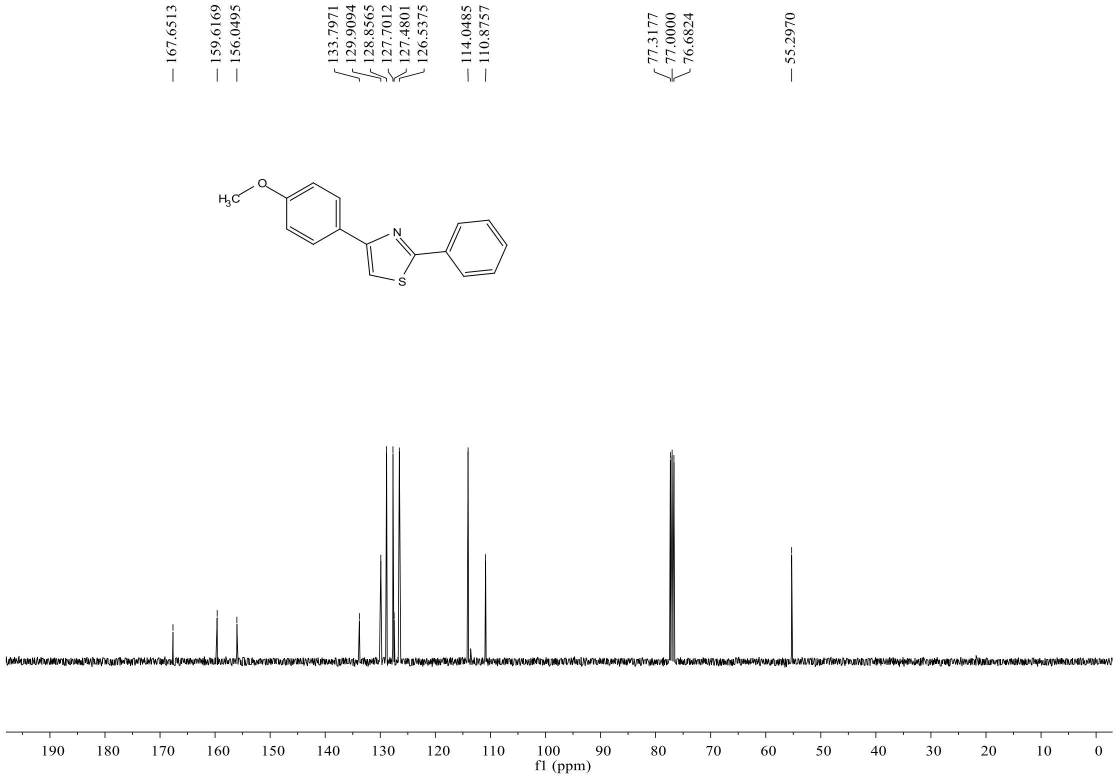

^{1}H, ^{13}C and ^{19}F NMR spectra of **3ag**

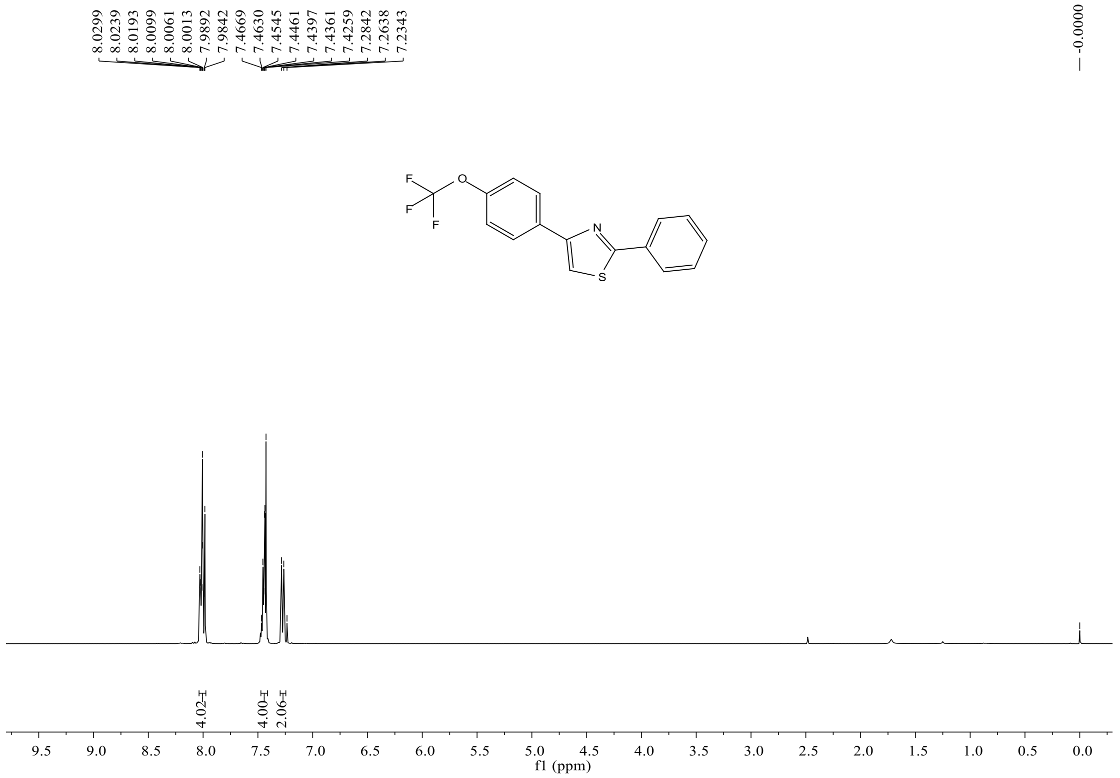

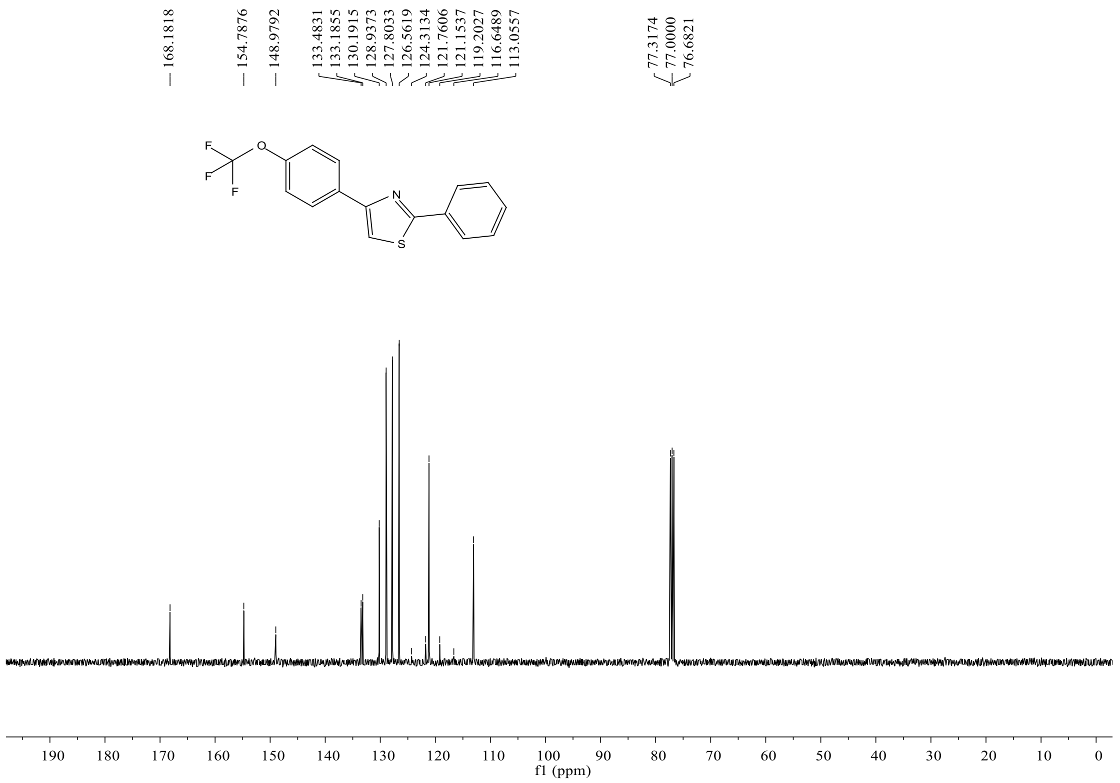

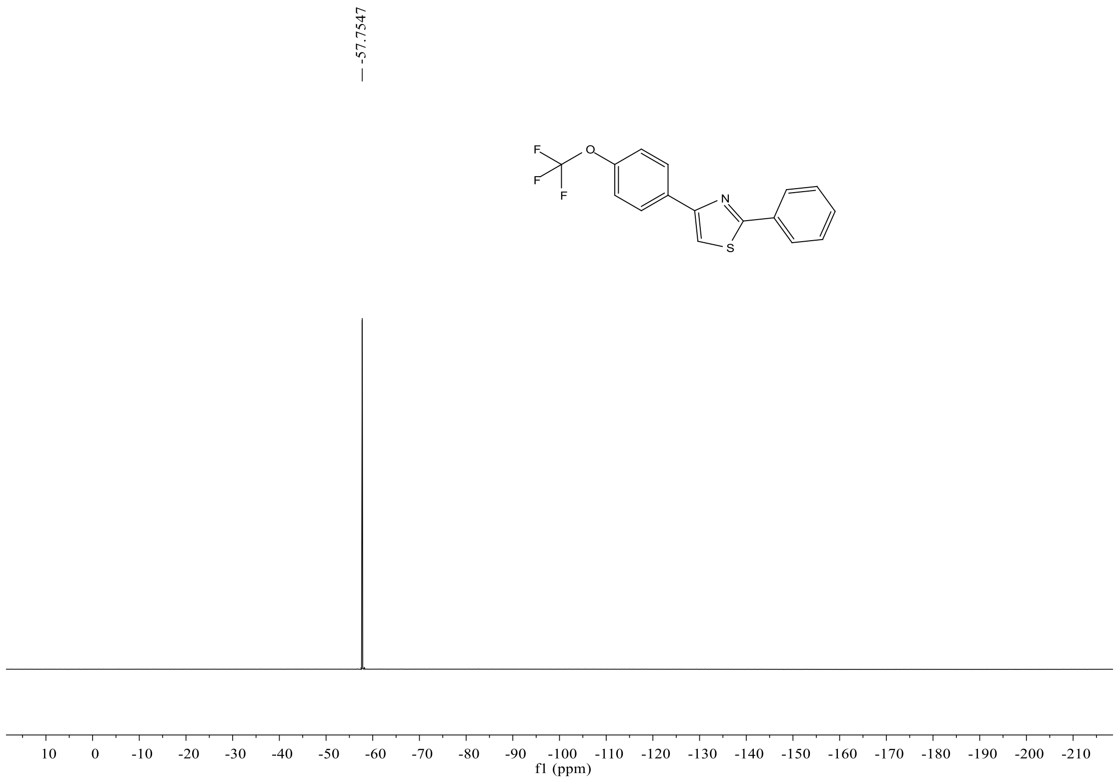

^{1}H and ^{13}C NMR spectra of **3ah**

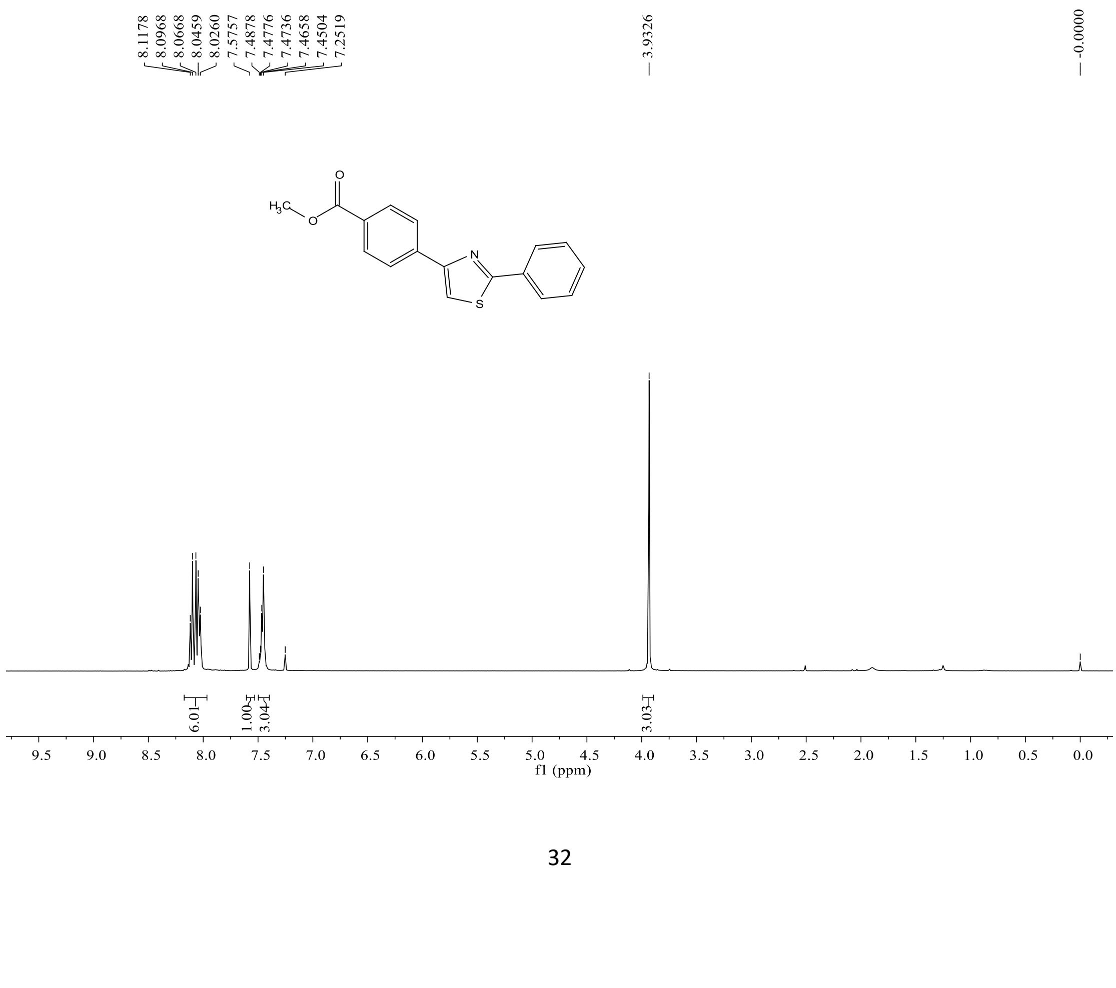

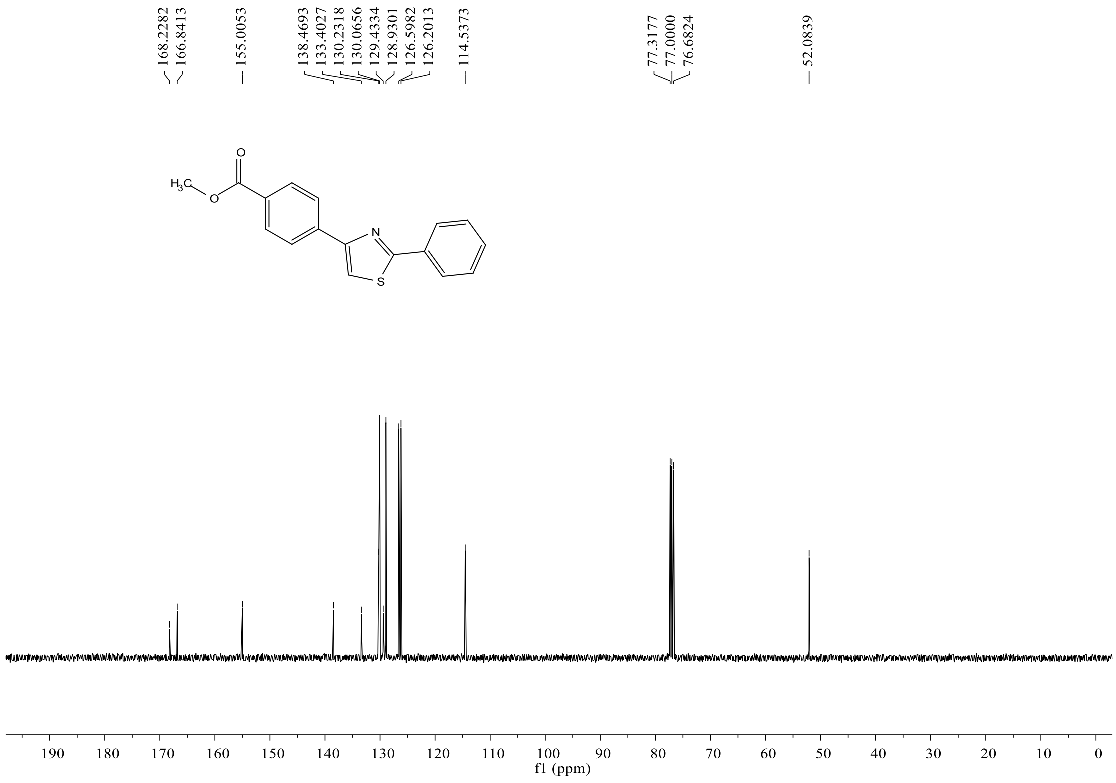

^{1}H, ^{13}C and ^{19}F NMR spectra of **3ai**

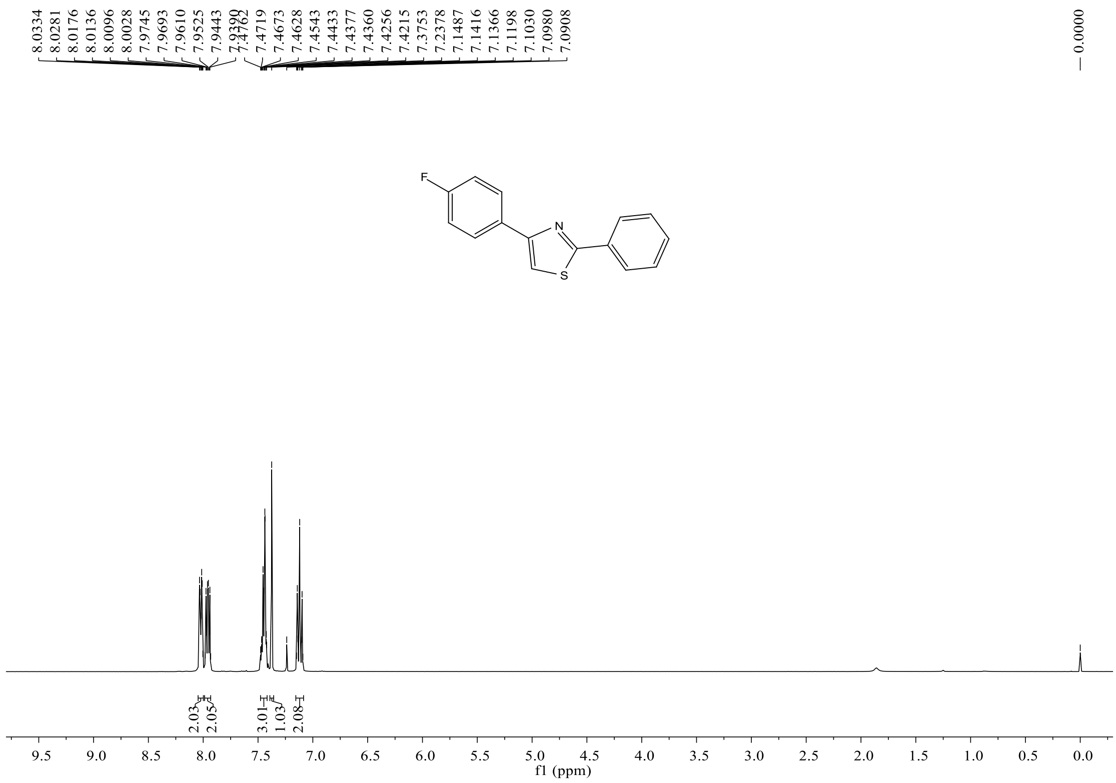

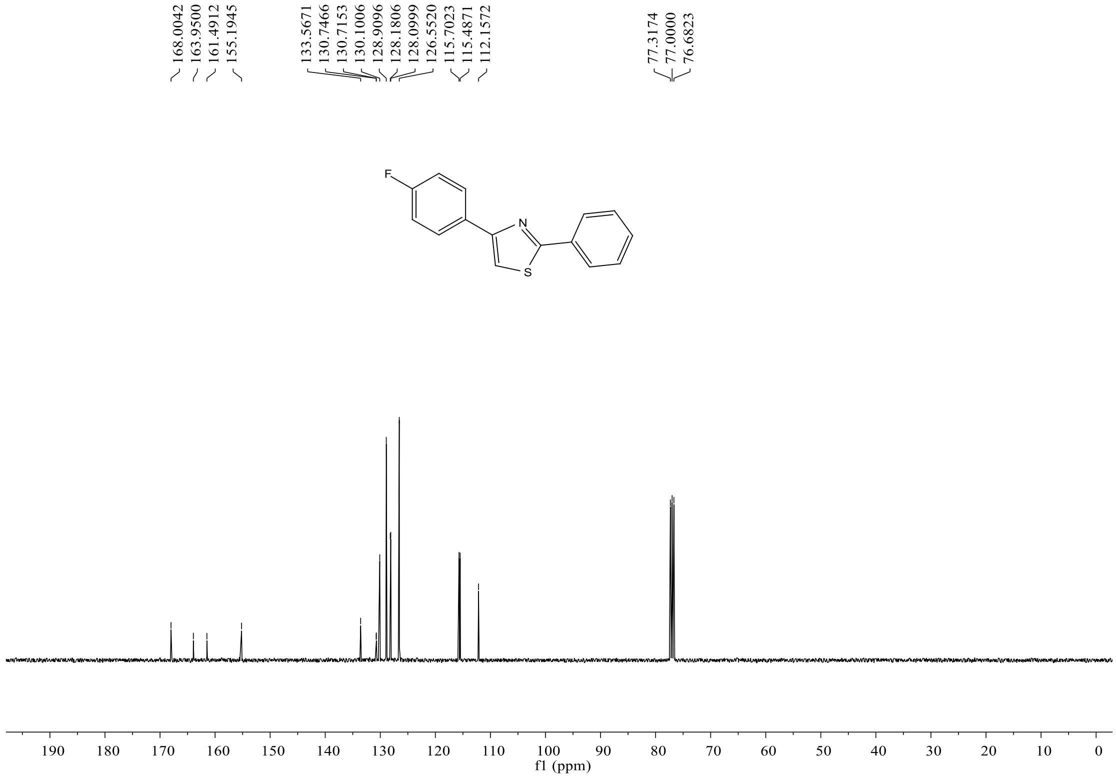
168.0042
163.9500
161.4912
155.1945
133.5671
130.7466
130.7153
130.1006
128.9096
128.1806
128.0999
126.5520
115.7023
115.4871
112.1572
77.3174
77.0000
76.6823
F
N
S
f1 (ppm)

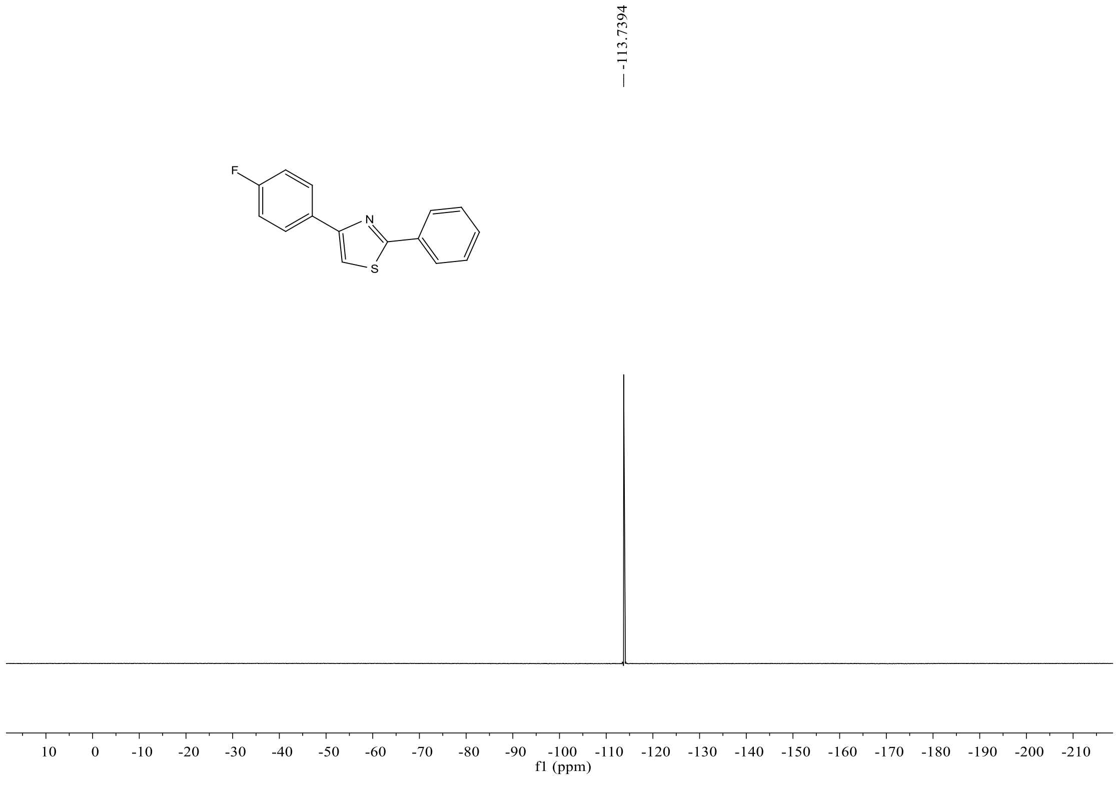
-113.7394
F
N
S
f1 (ppm)

^{1}H and ^{13}C NMR spectra of **3aj**

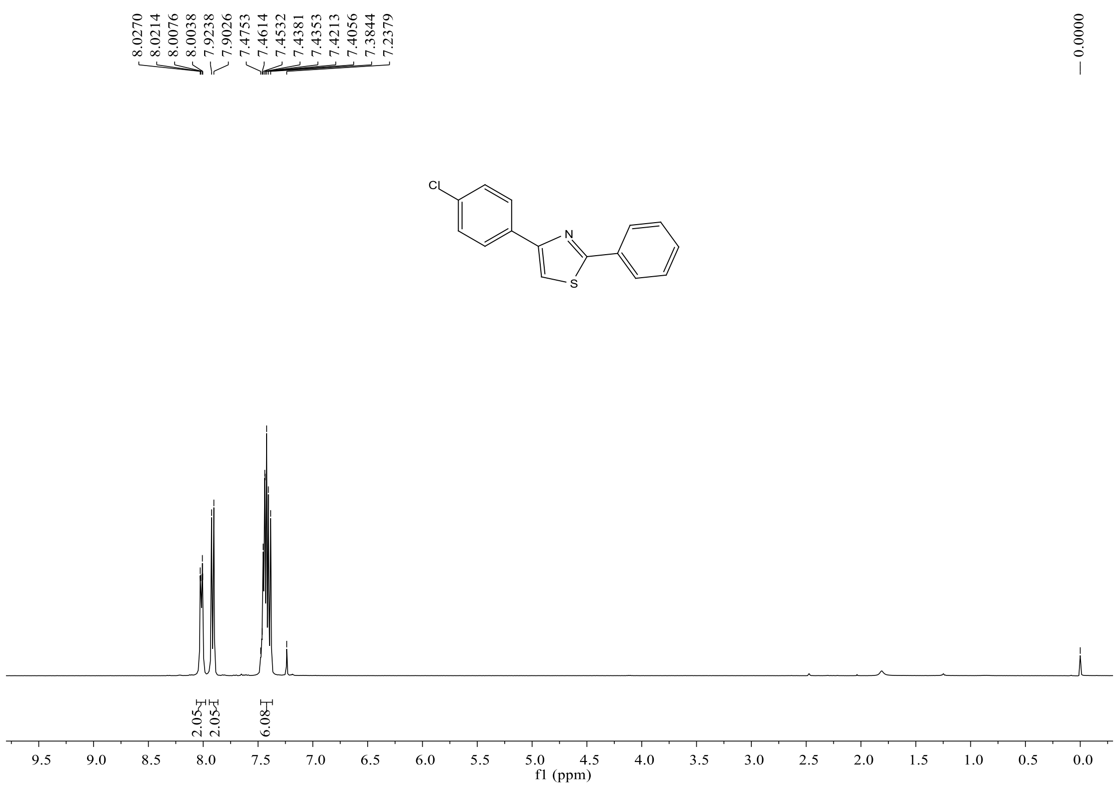

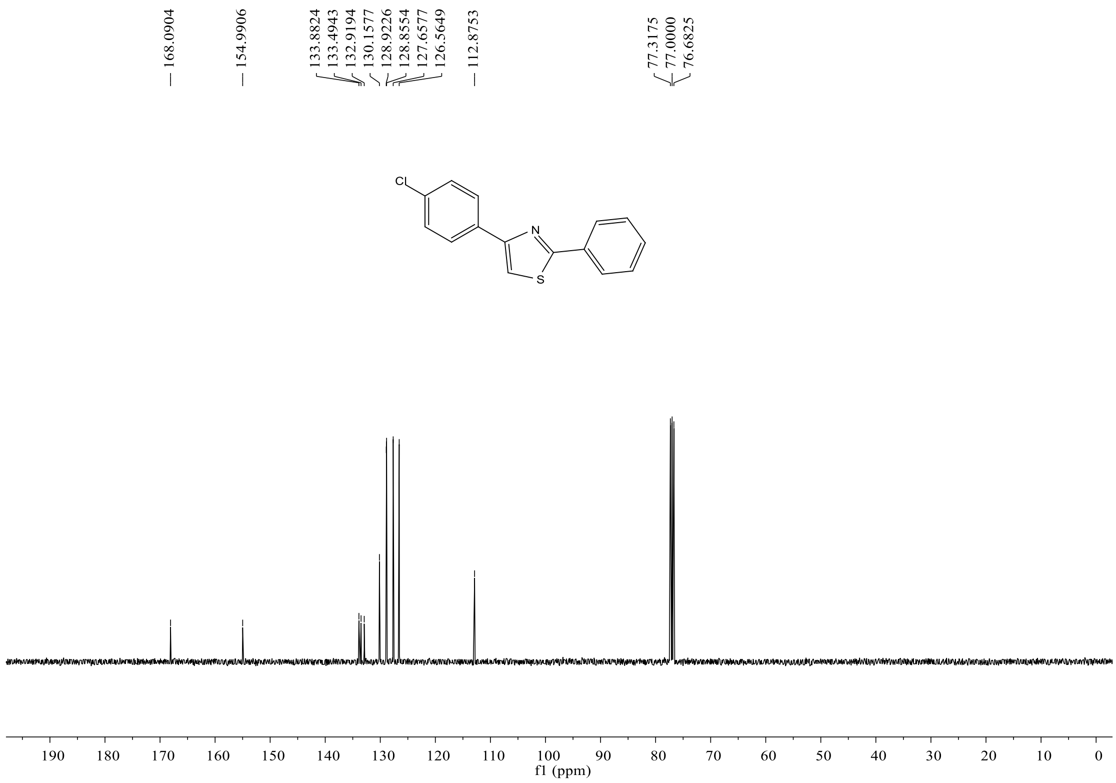

^{1}H and ^{13}C NMR spectra of **3ak**

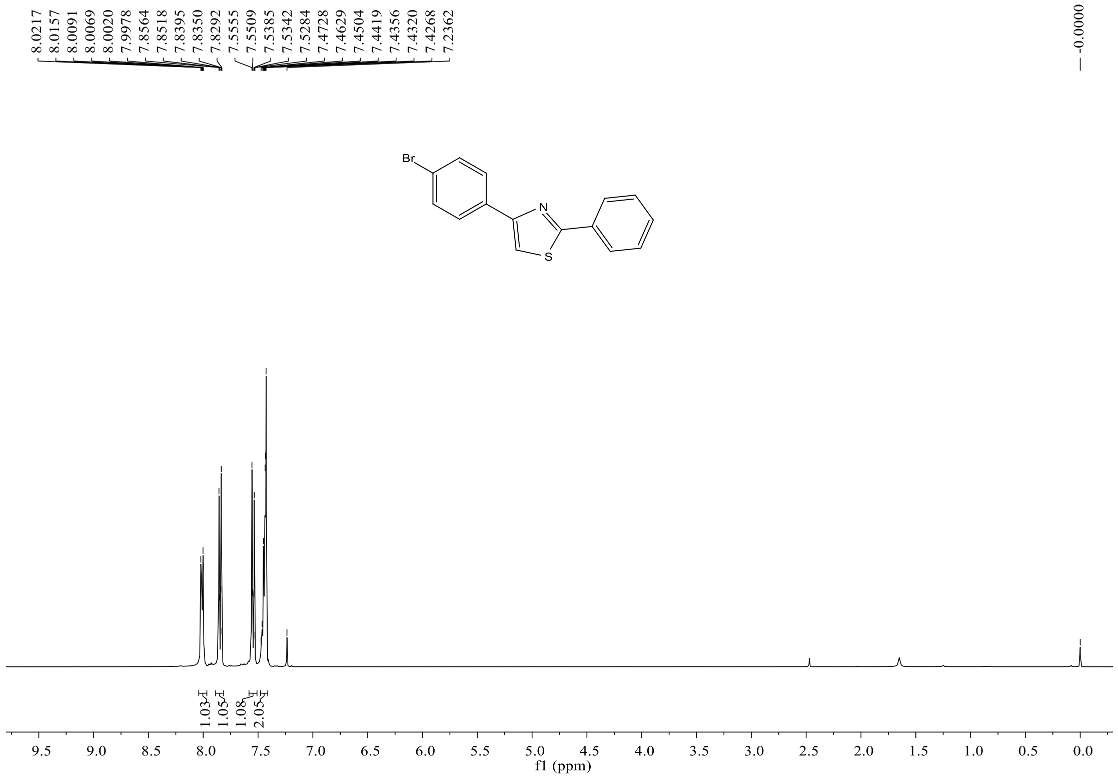

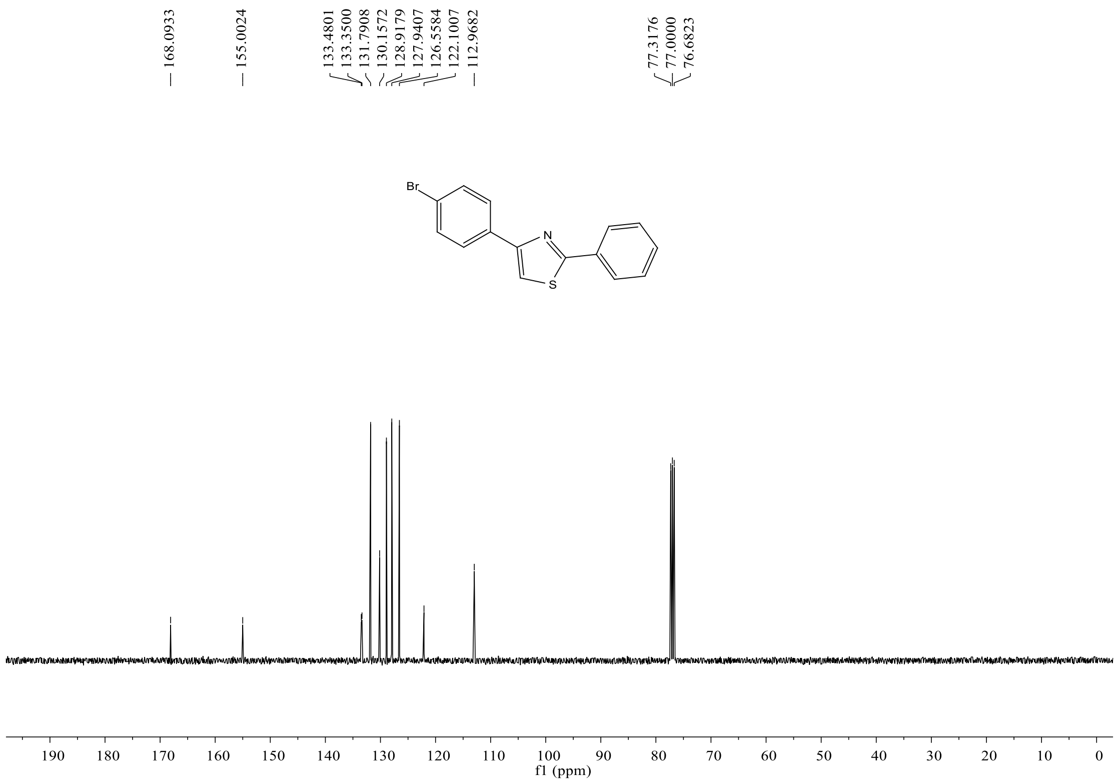

^1H and ^{13}C NMR spectra of **3al**

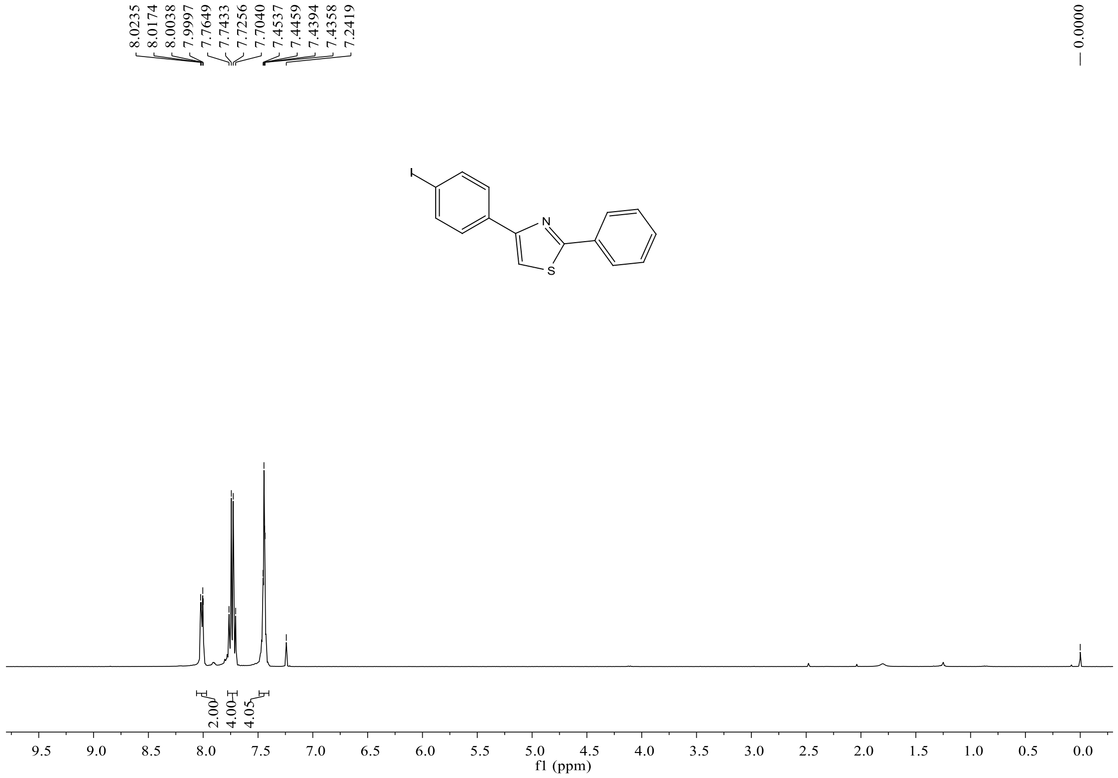

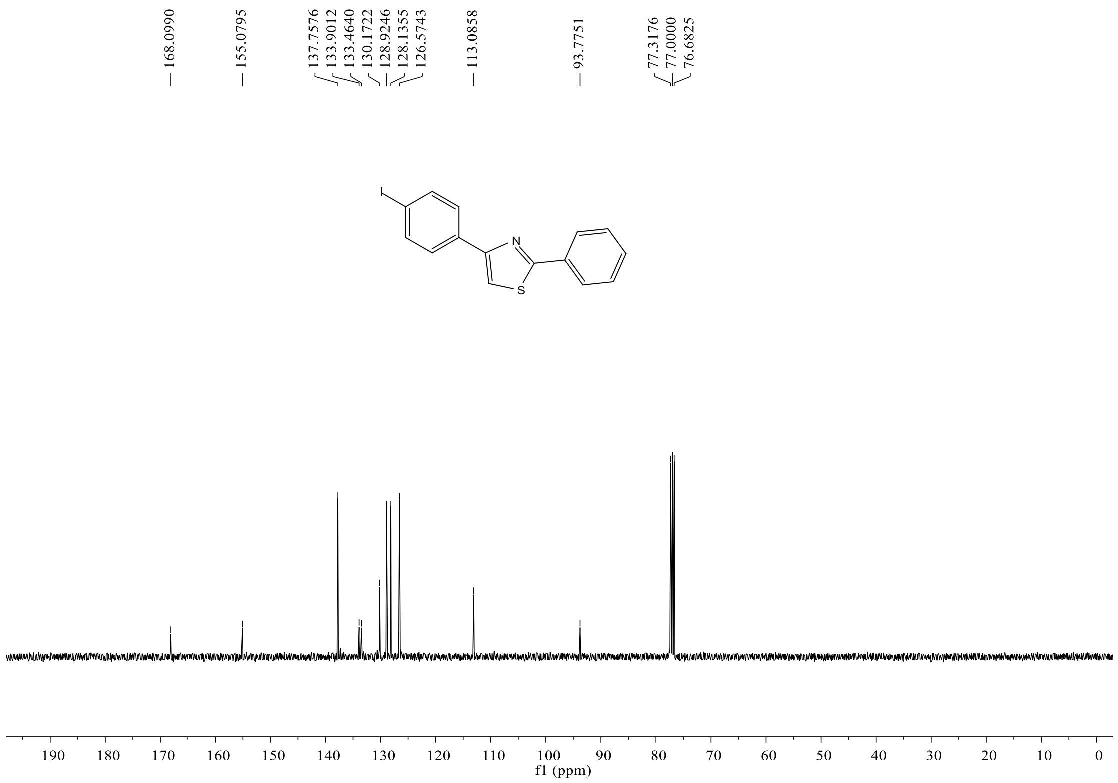

^{1}H and ^{13}C NMR spectra of **3am**

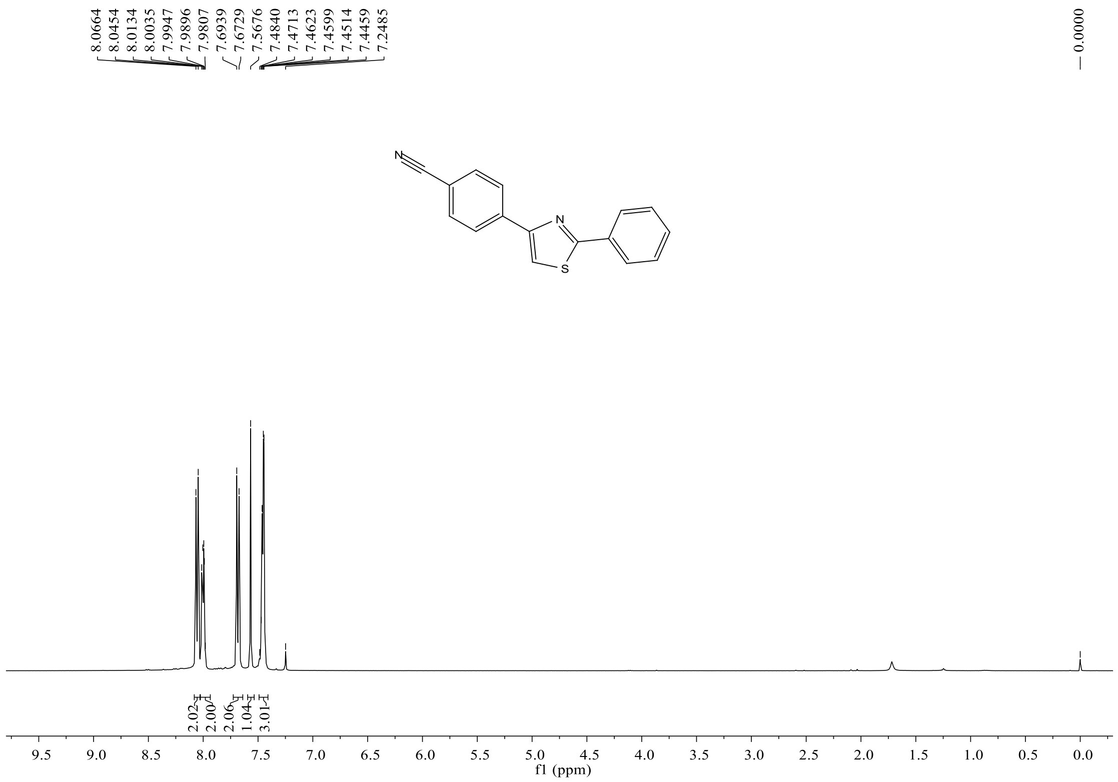

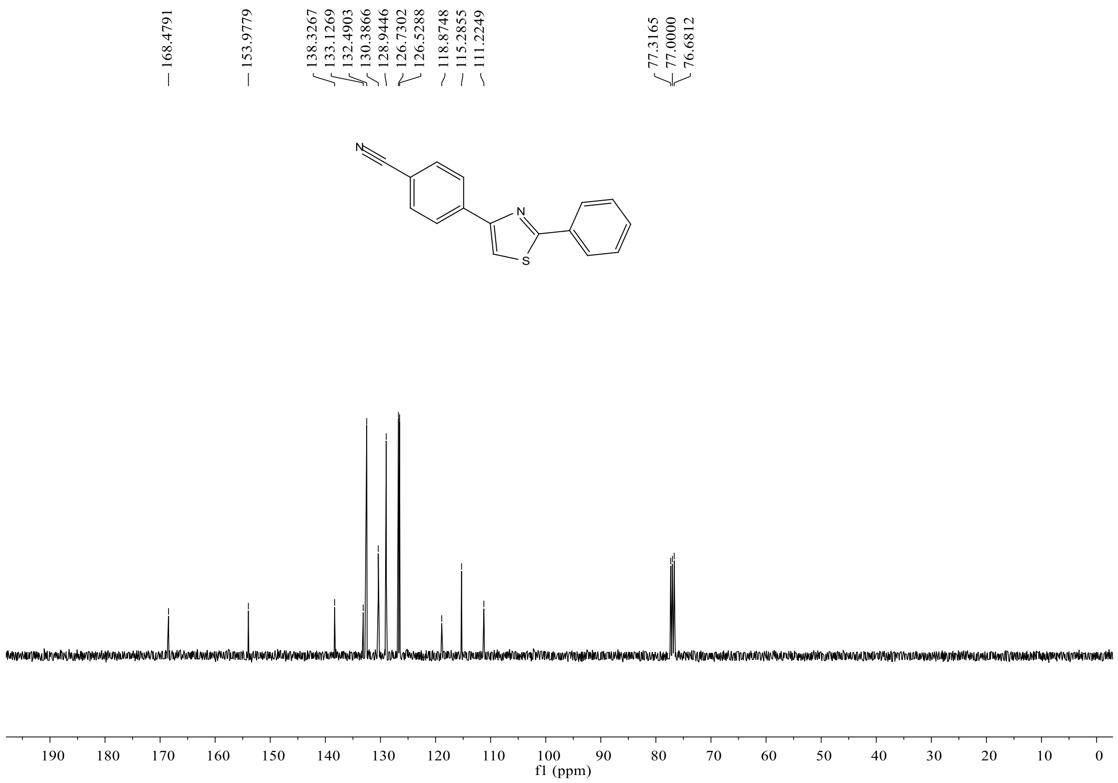

^{1}H and ^{13}C NMR spectra of **3an**

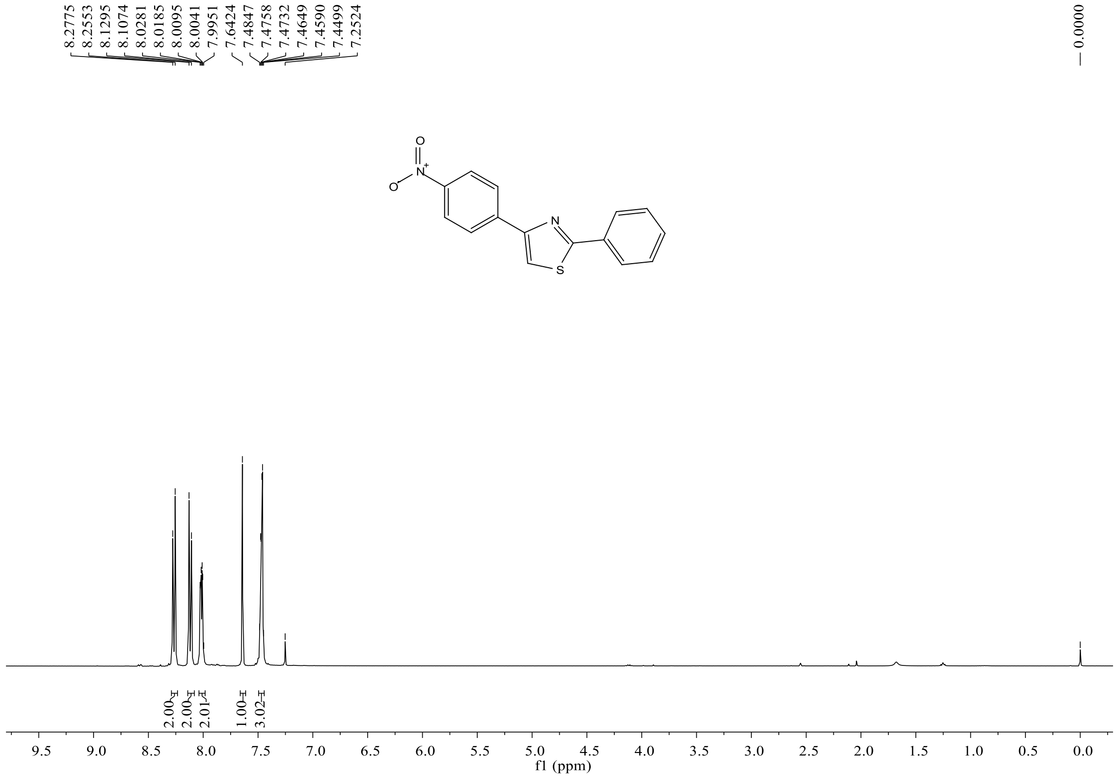

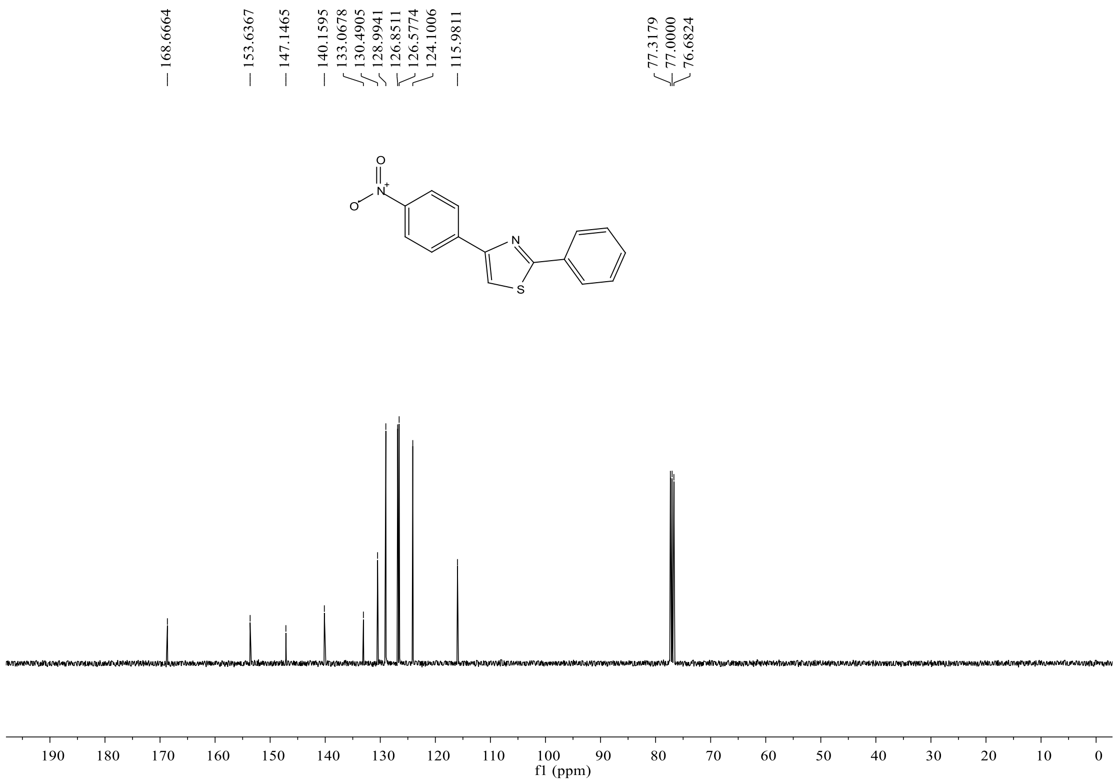

^{1}H and ^{13}C NMR spectra of **3ao**

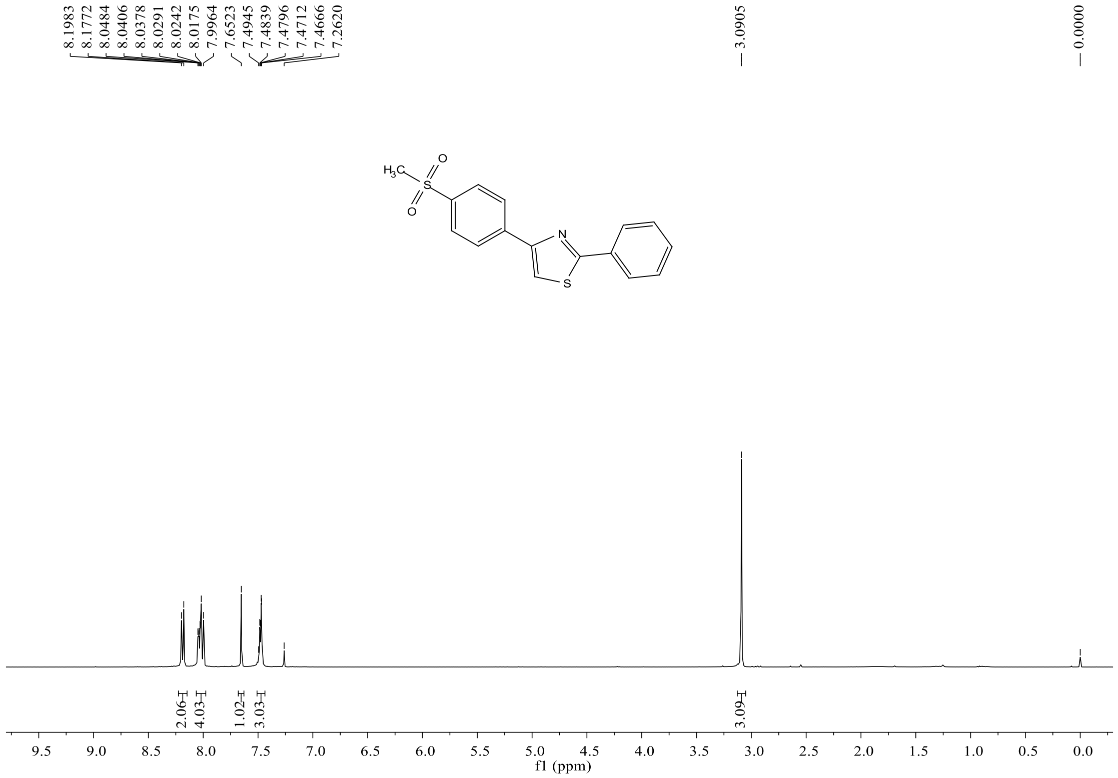

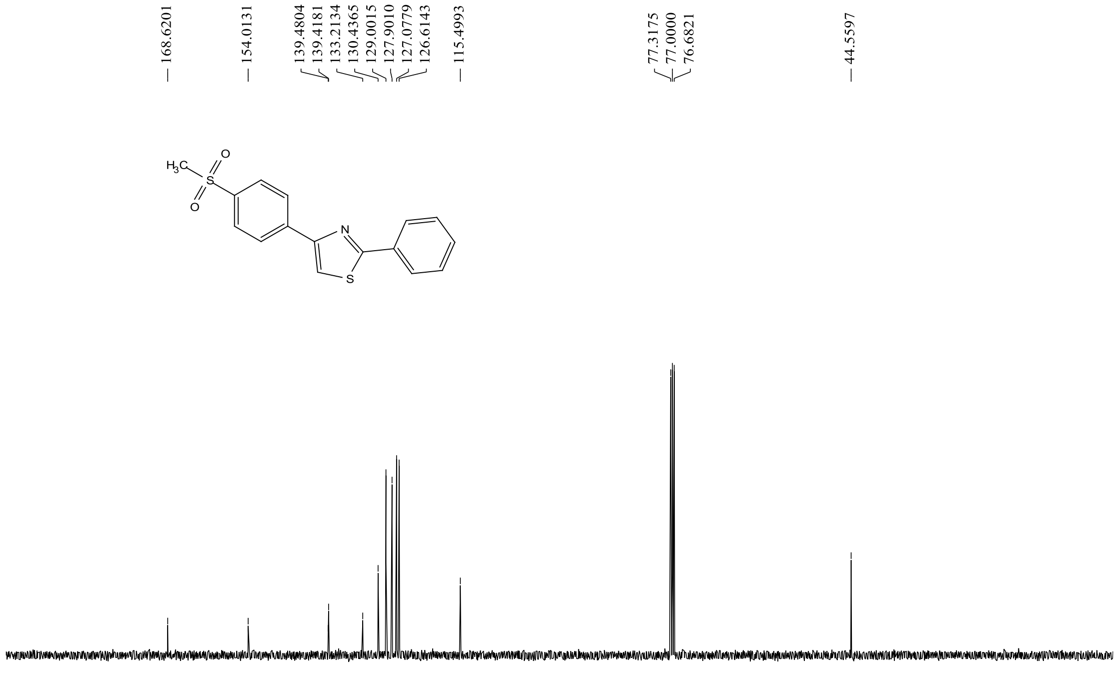

^{1}H and ^{13}C NMR spectra of **3ap**

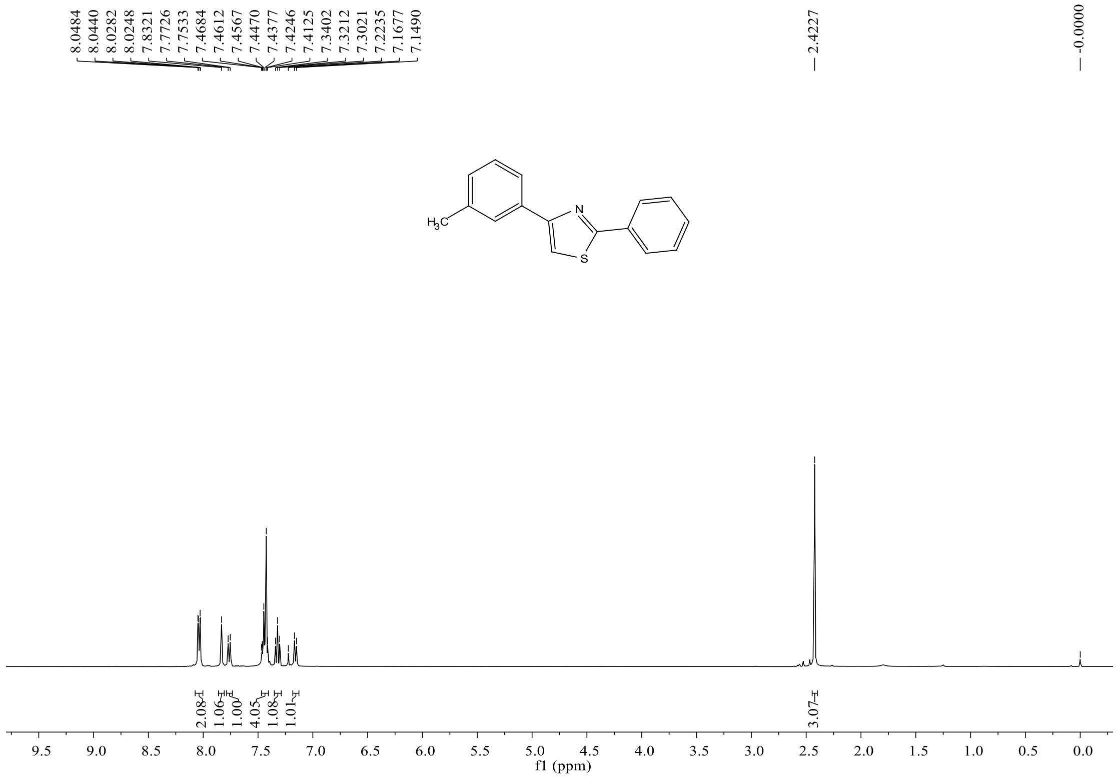

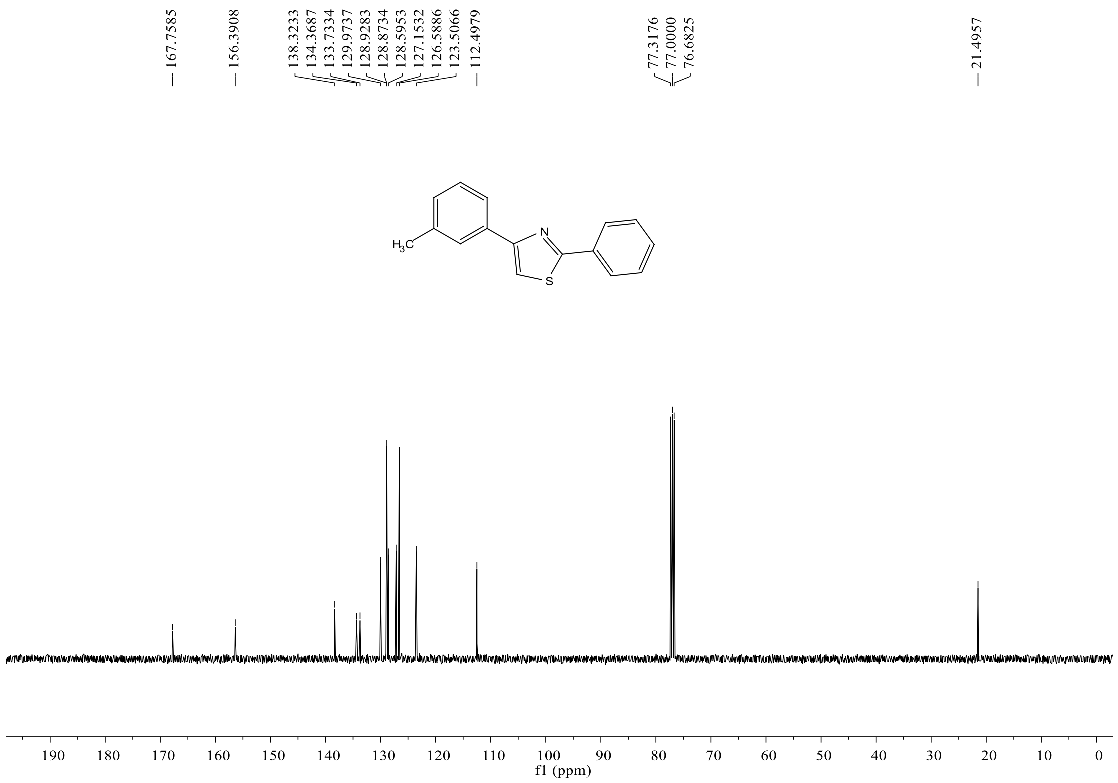

^{1}H, ^{13}C and ^{19}F NMR spectra of **3aq**

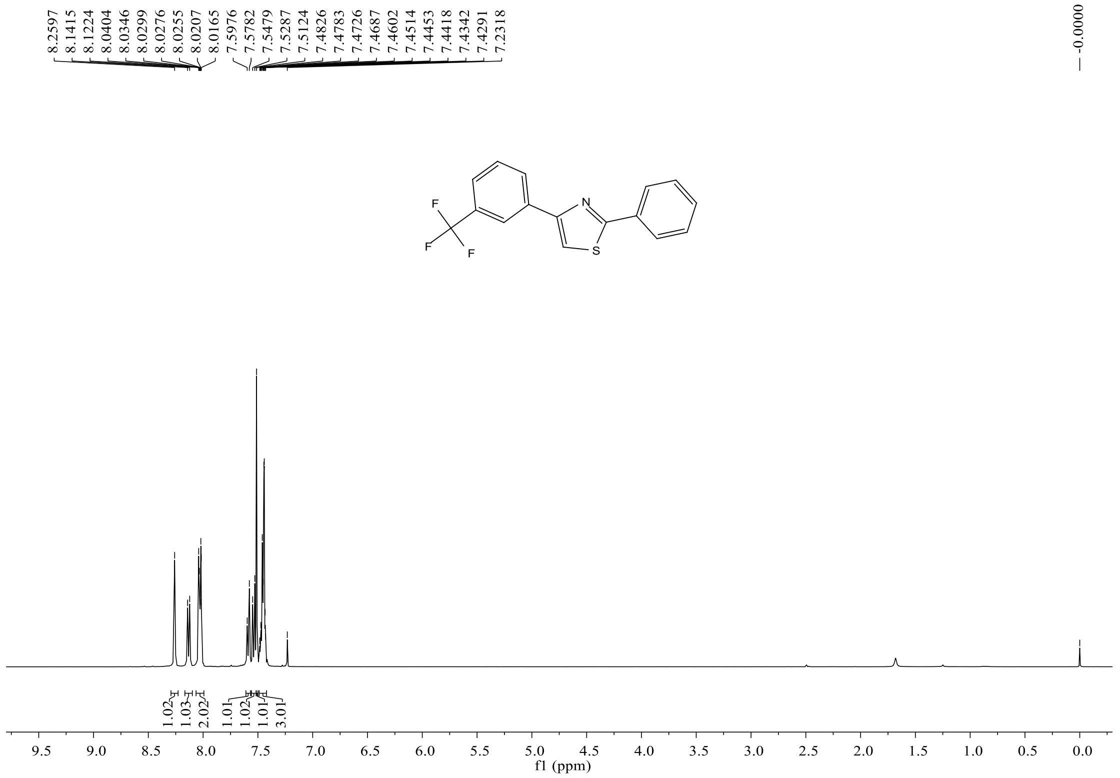

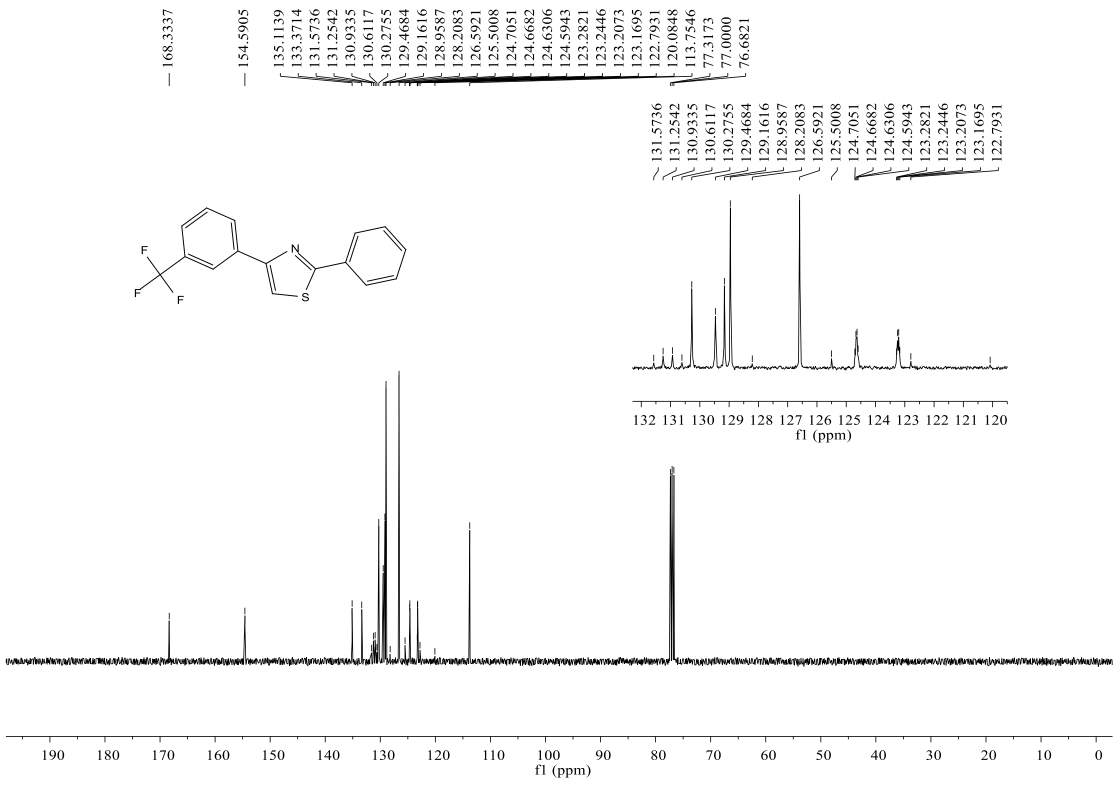

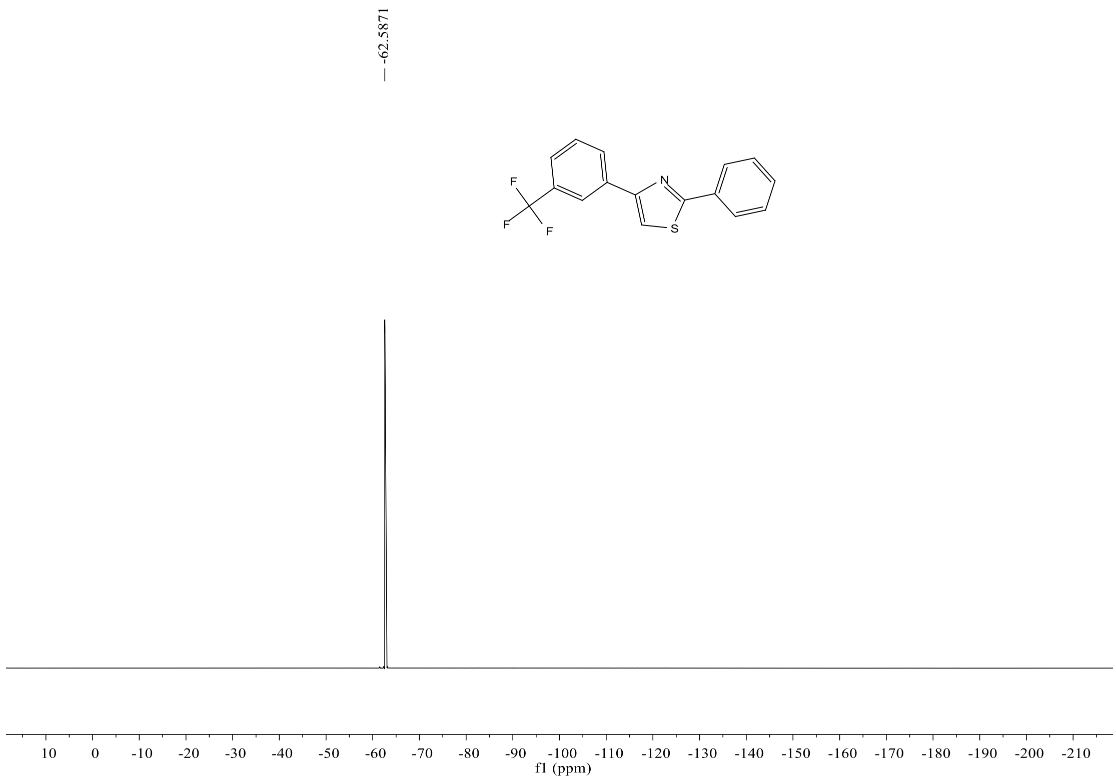

^{1}H, ^{13}C and ^{19}F NMR spectra of **3ar**

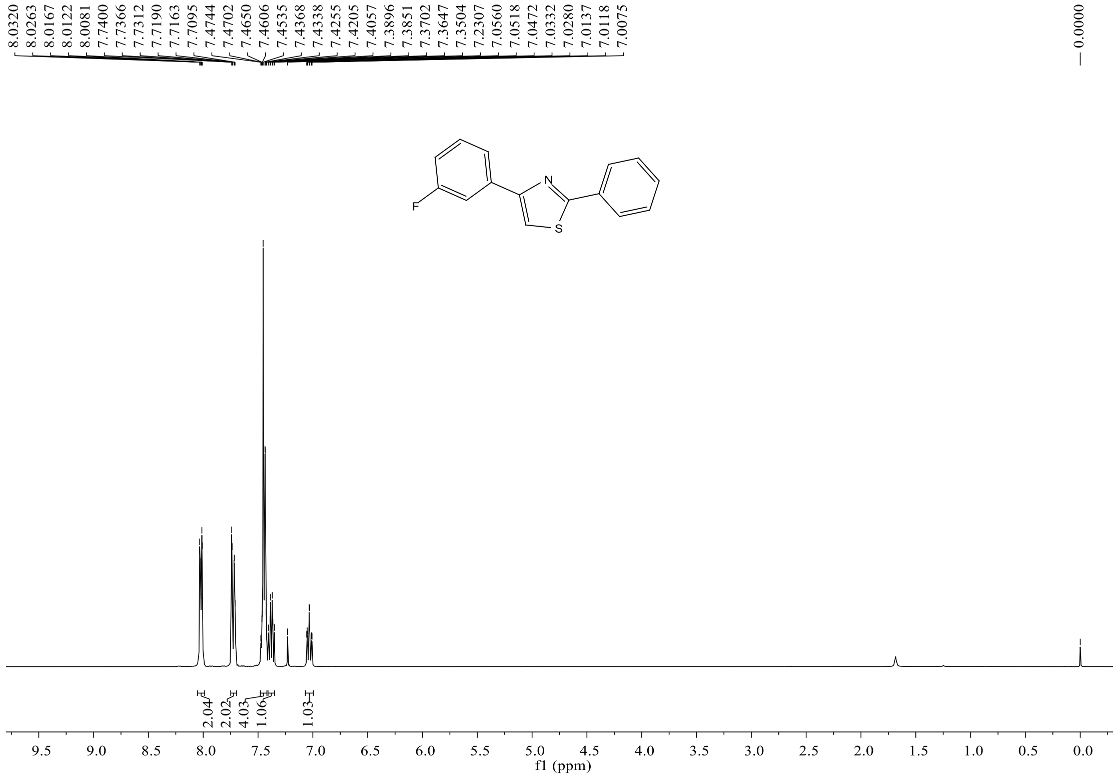

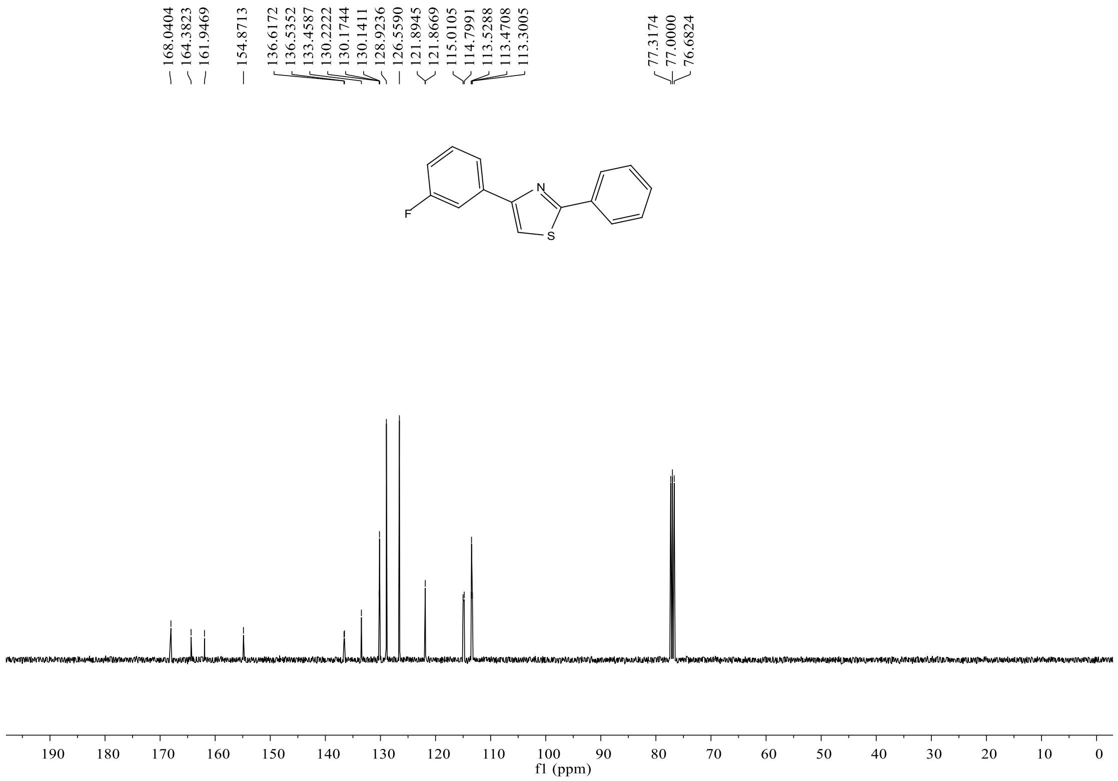
168.0404
164.3823
161.9469
154.8713
136.6172
136.5352
133.4587
130.2222
130.1744
130.1411
128.9236
126.5590
121.8945
121.8669
115.0105
114.7991
113.5288
113.4708
113.3005
77.3174
77.0000
76.6824
F
N
S
190 180 170 160 150 140 130 120 110 100 90 80 70 60 50 40 30 20 10 0
f1 (ppm)

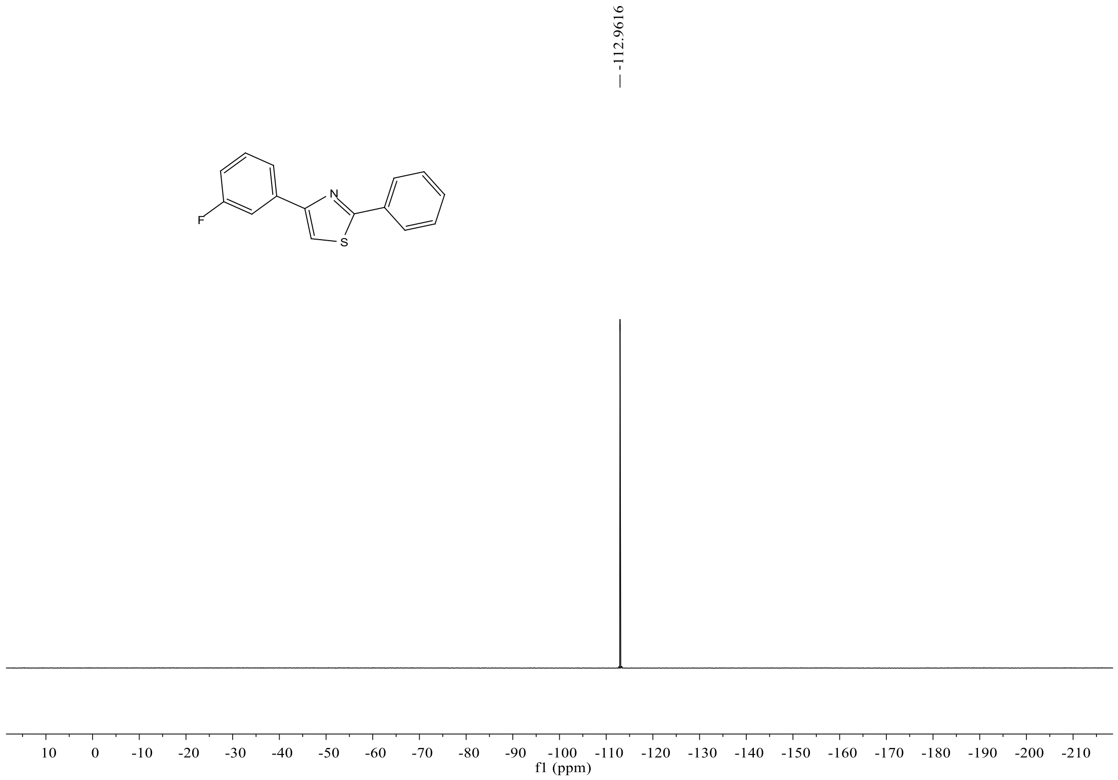
-112.9616
F
N
S
10 0 -10 -20 -30 -40 -50 -60 -70 -80 -90 -100 -110 -120 -130 -140 -150 -160 -170 -180 -190 -200 -210
f1 (ppm)

^{1}H and ^{13}C NMR spectra of **3as**

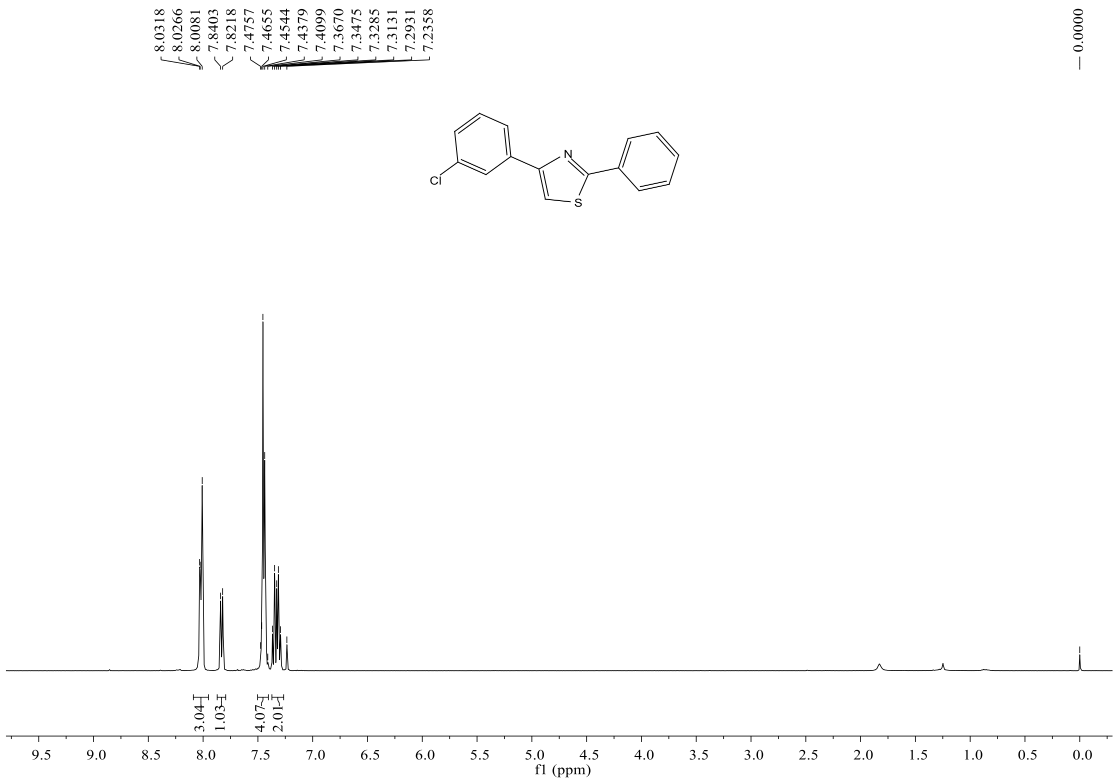

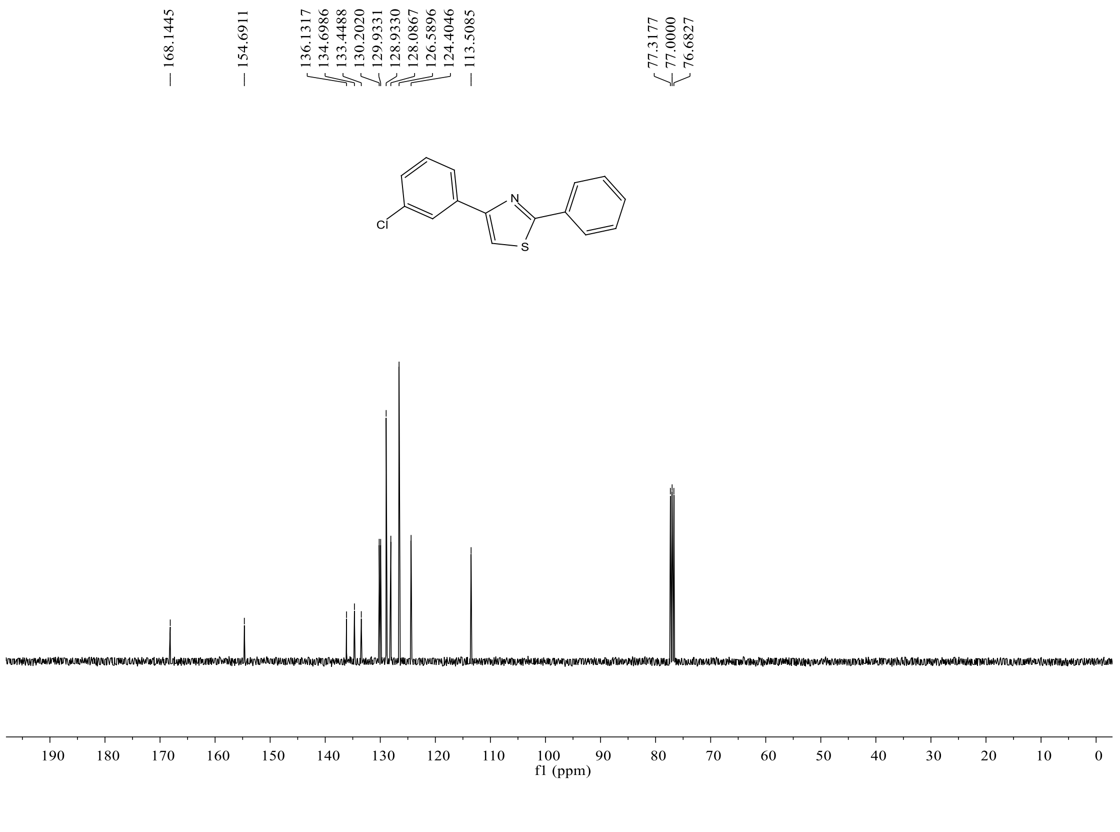

^{1}H and ^{13}C NMR spectra of **3at**

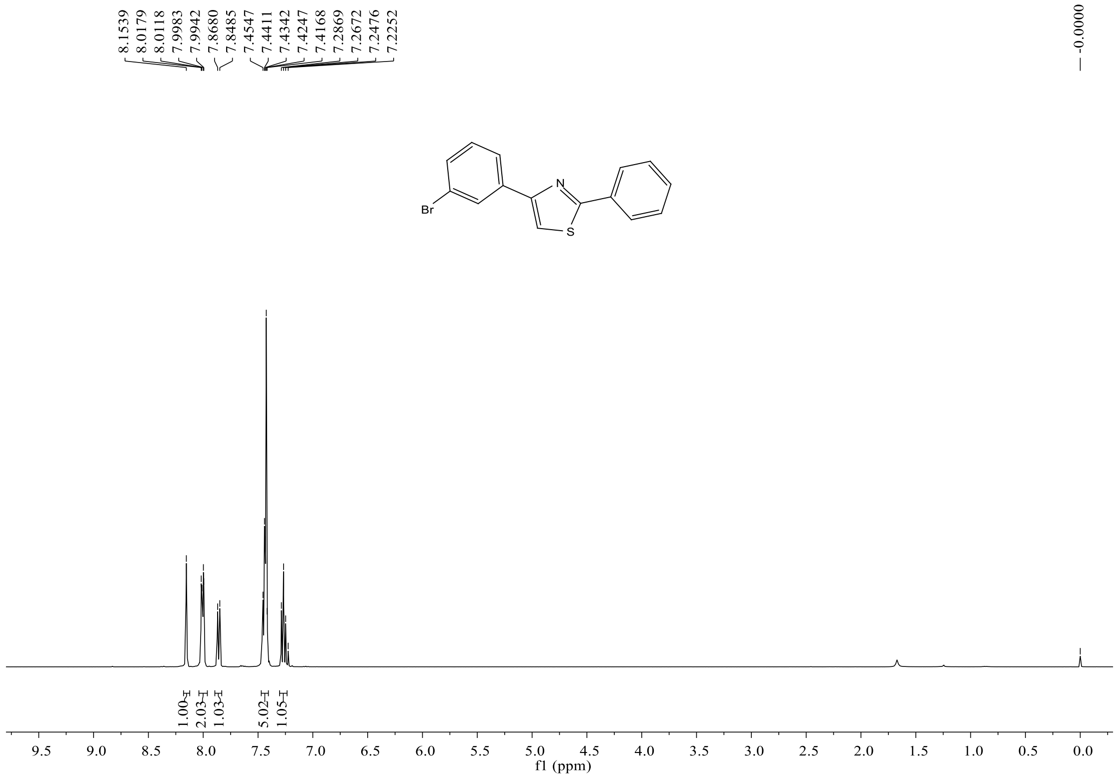

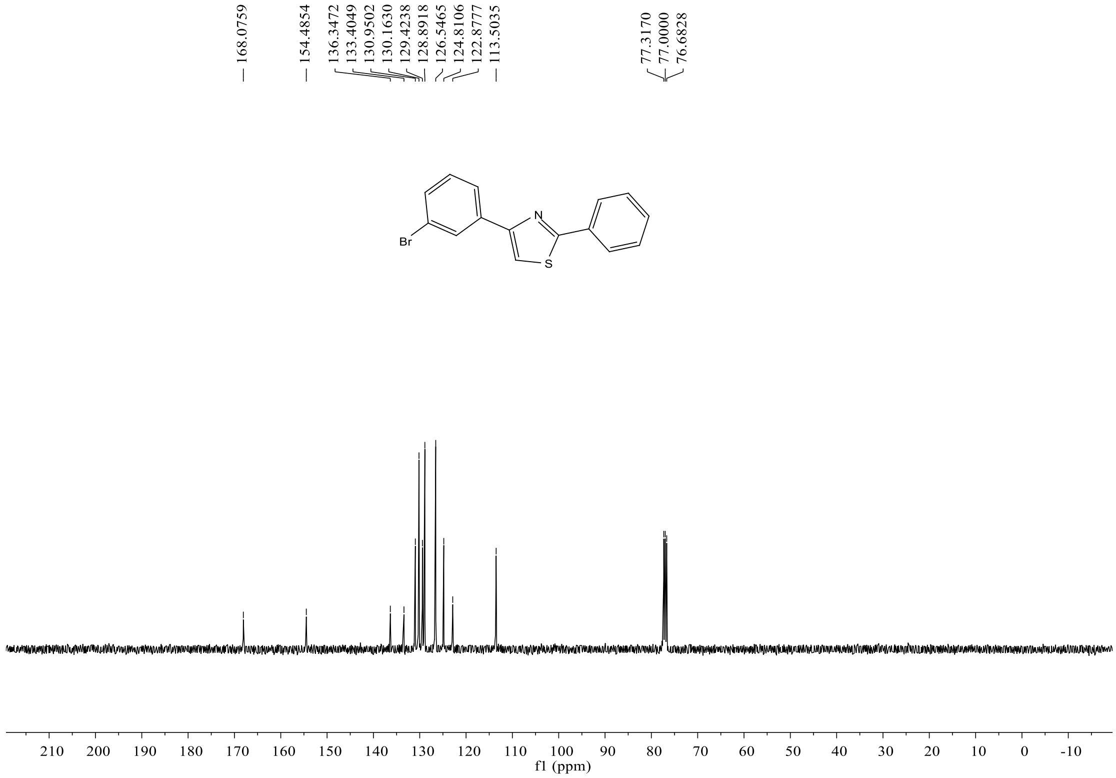

^{1}H and ^{13}C NMR spectra of **3au**

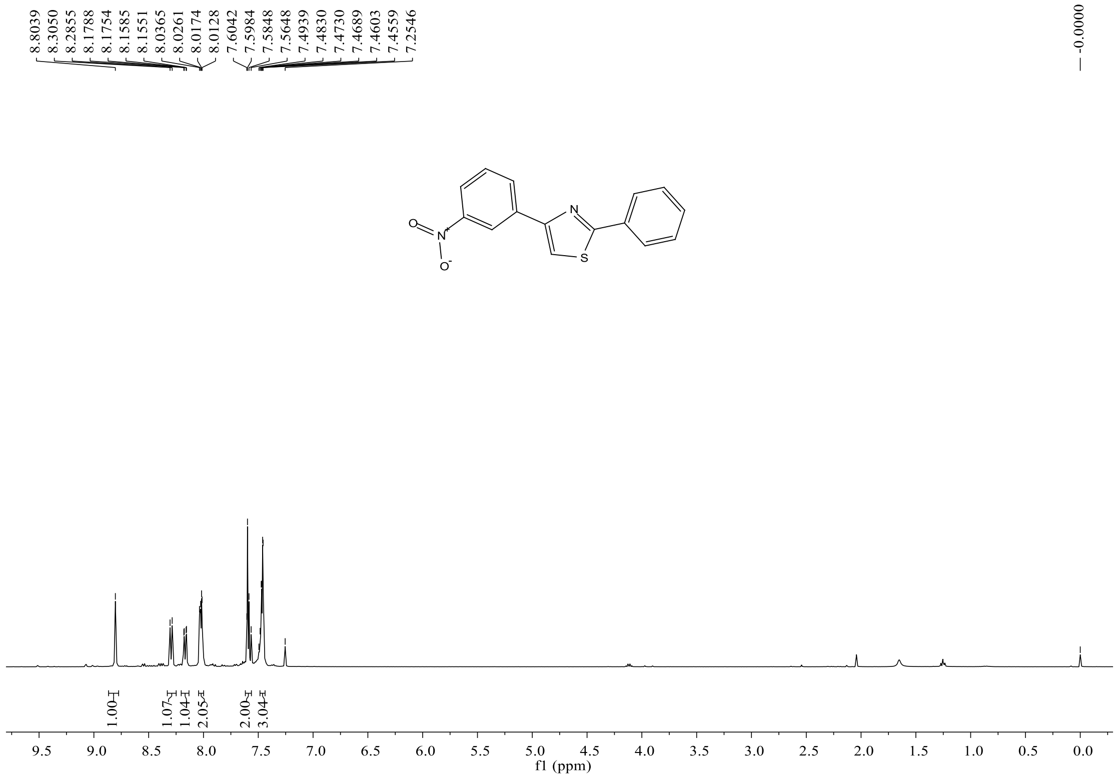

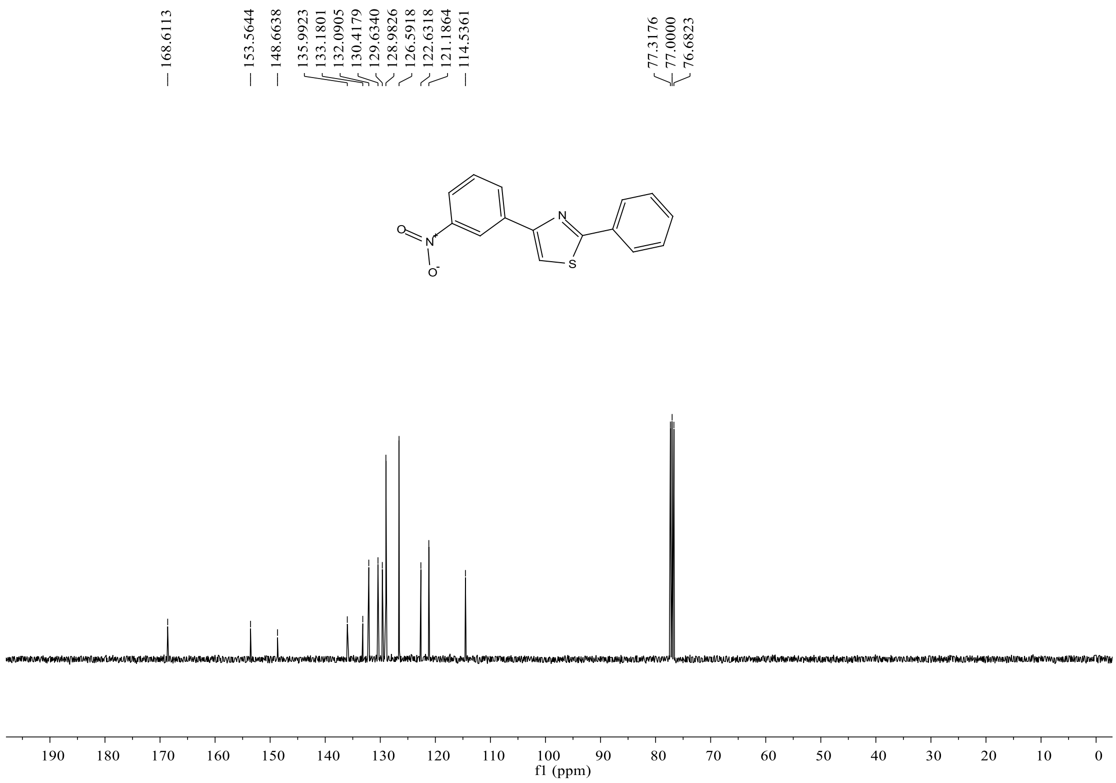

^{1}H, ^{13}C and ^{19}F NMR spectra of **3av**

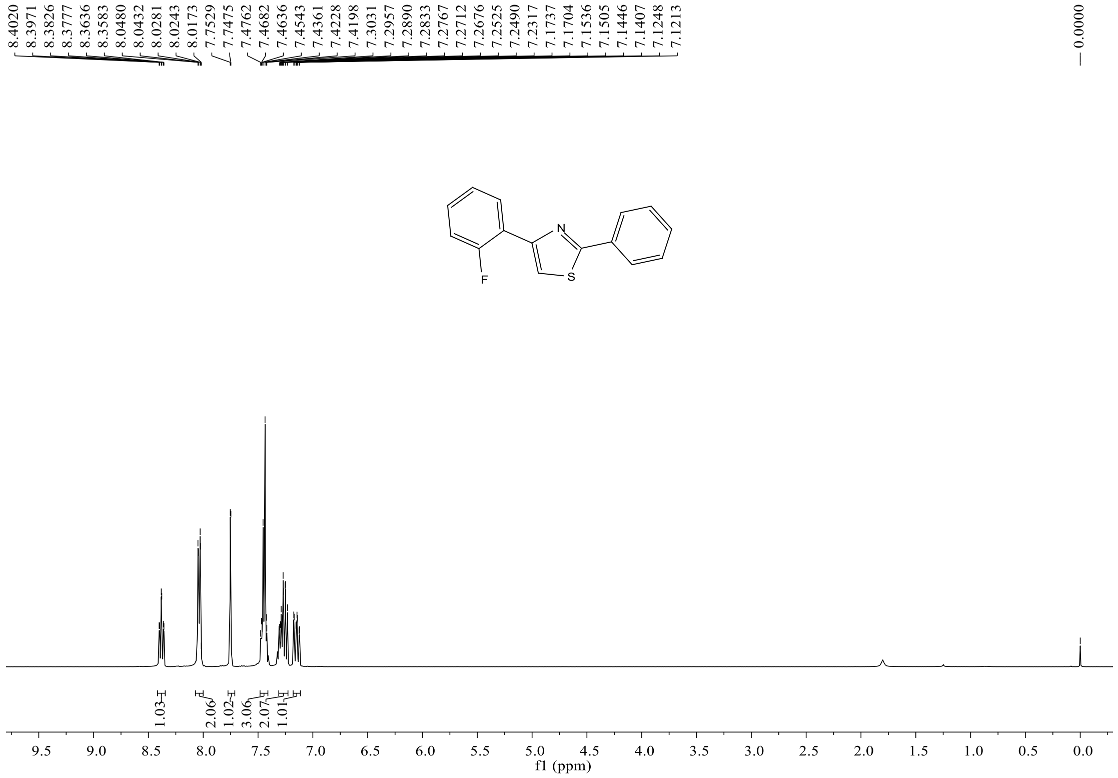

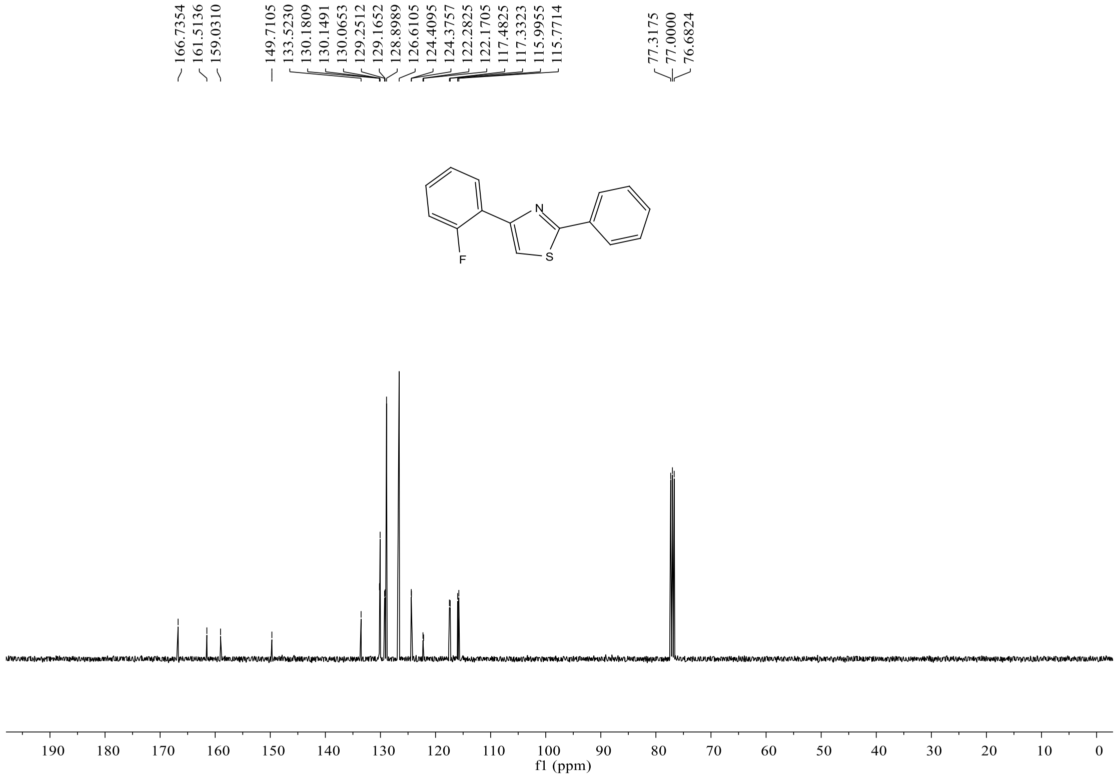

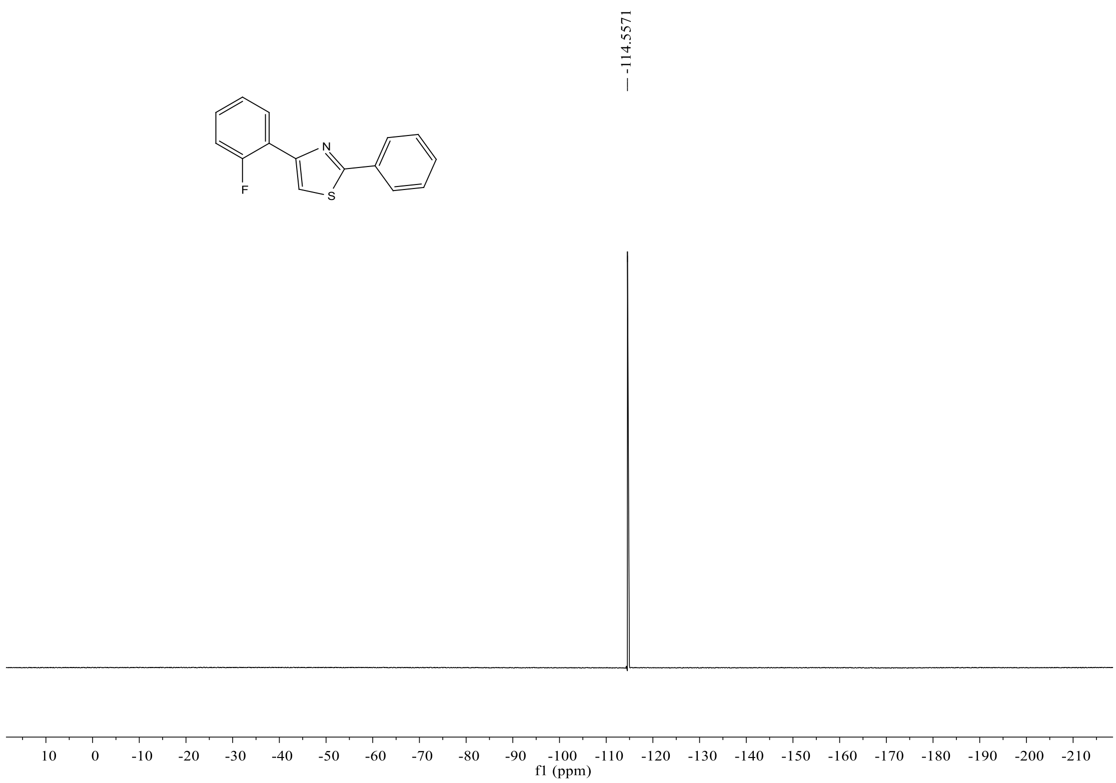

^{1}H and ^{13}C NMR spectra of **3aw**

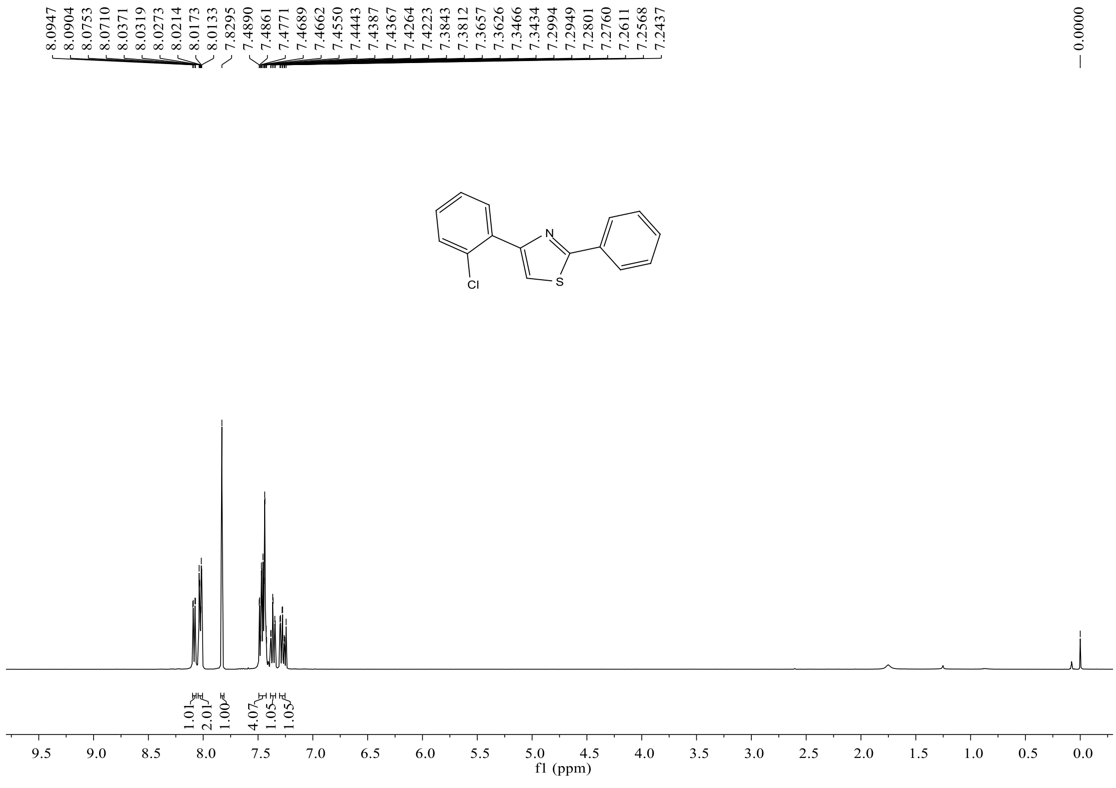

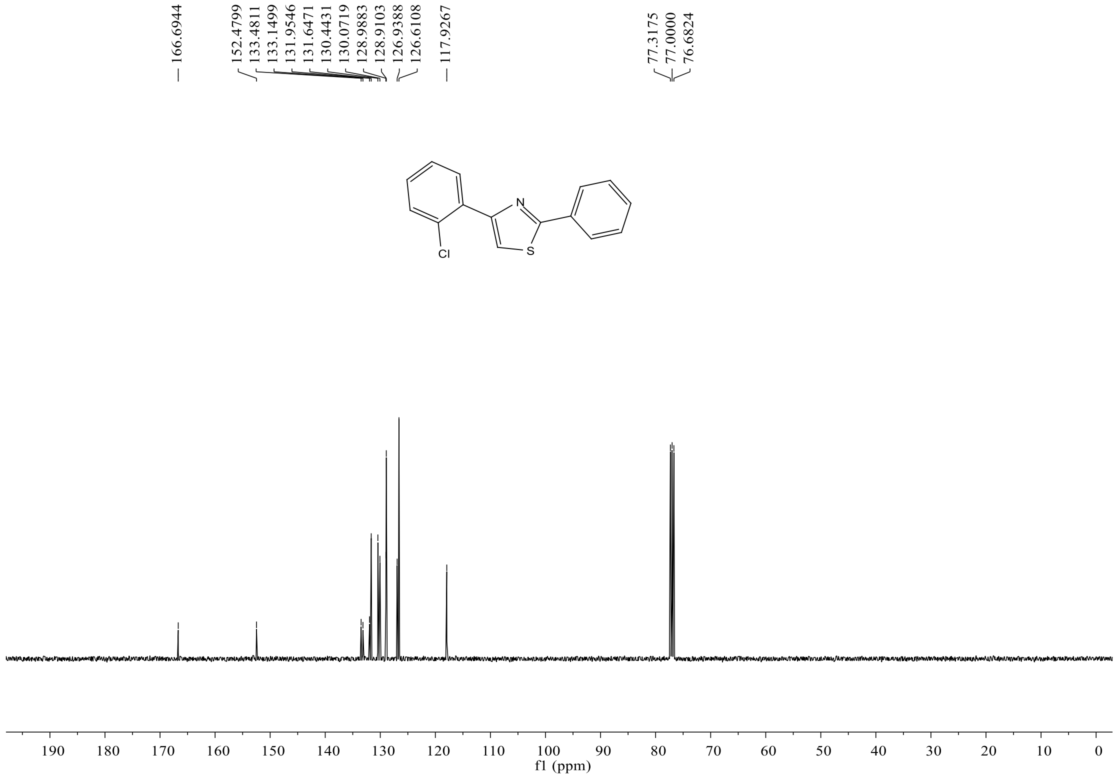

^{1}H and ^{13}C NMR spectra of **3ax**

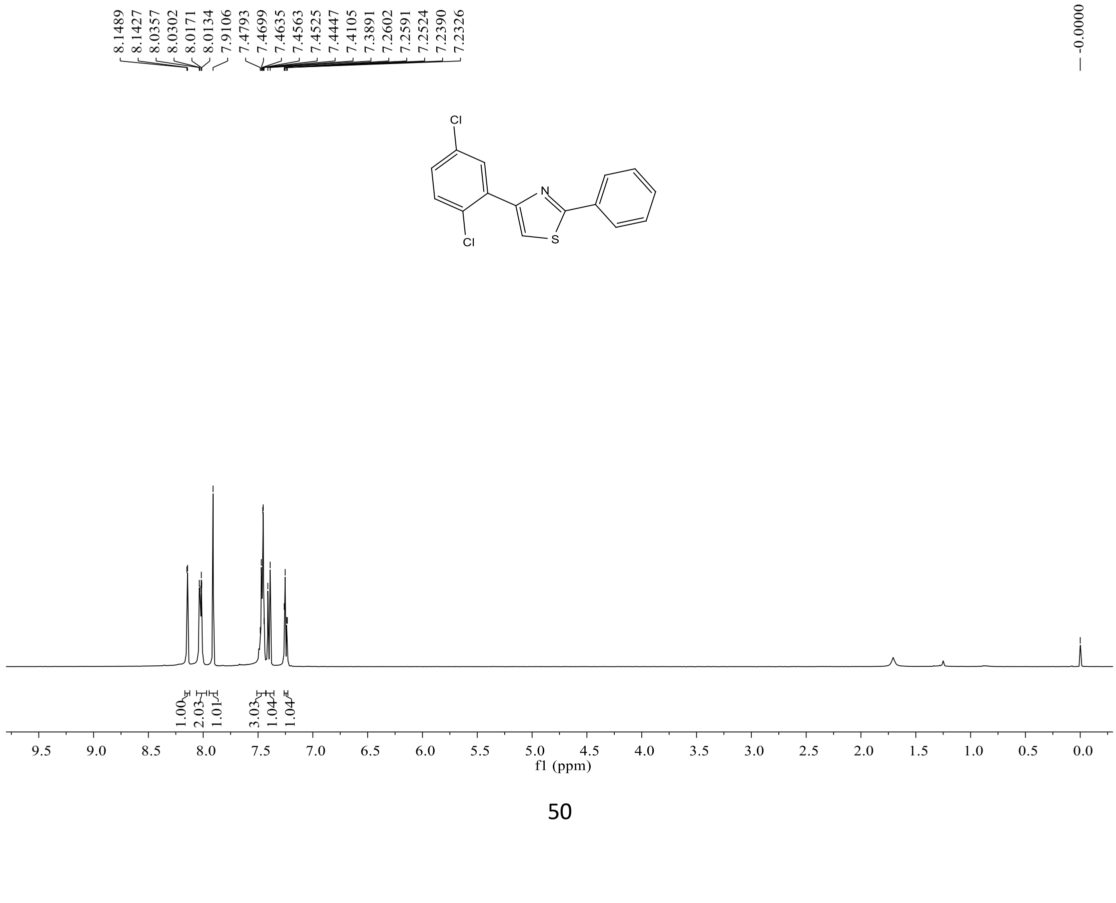

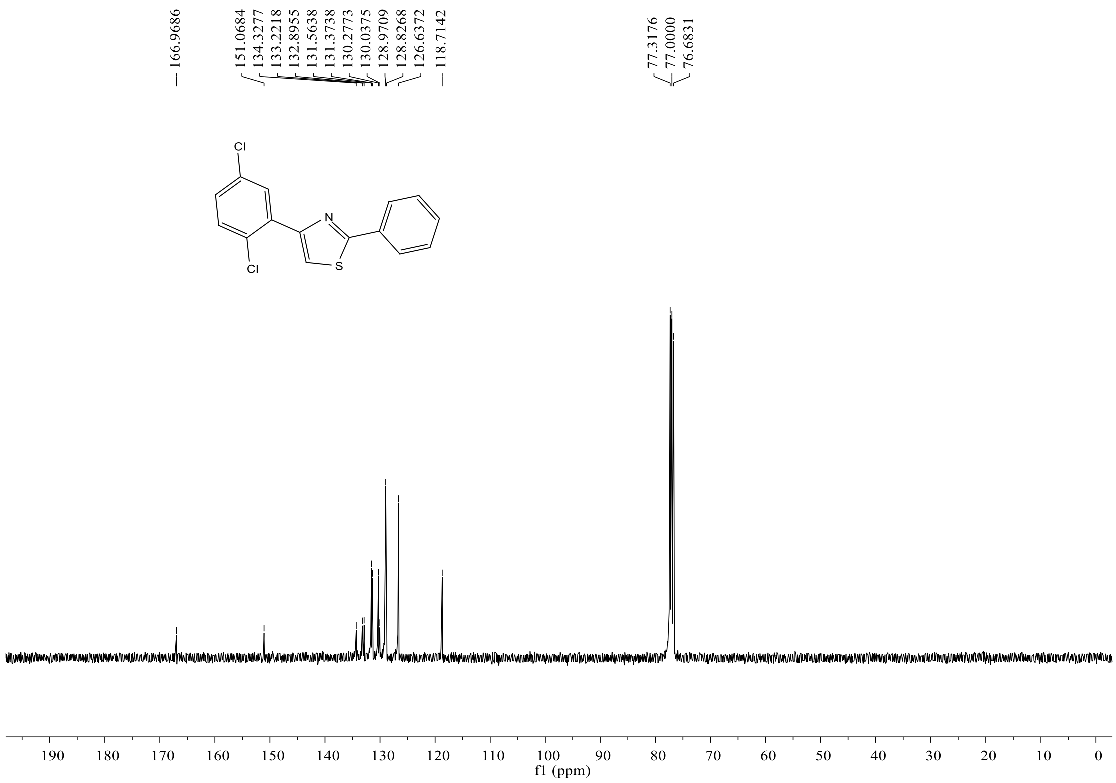

^{1}H and ^{13}C NMR spectra of **3ay**

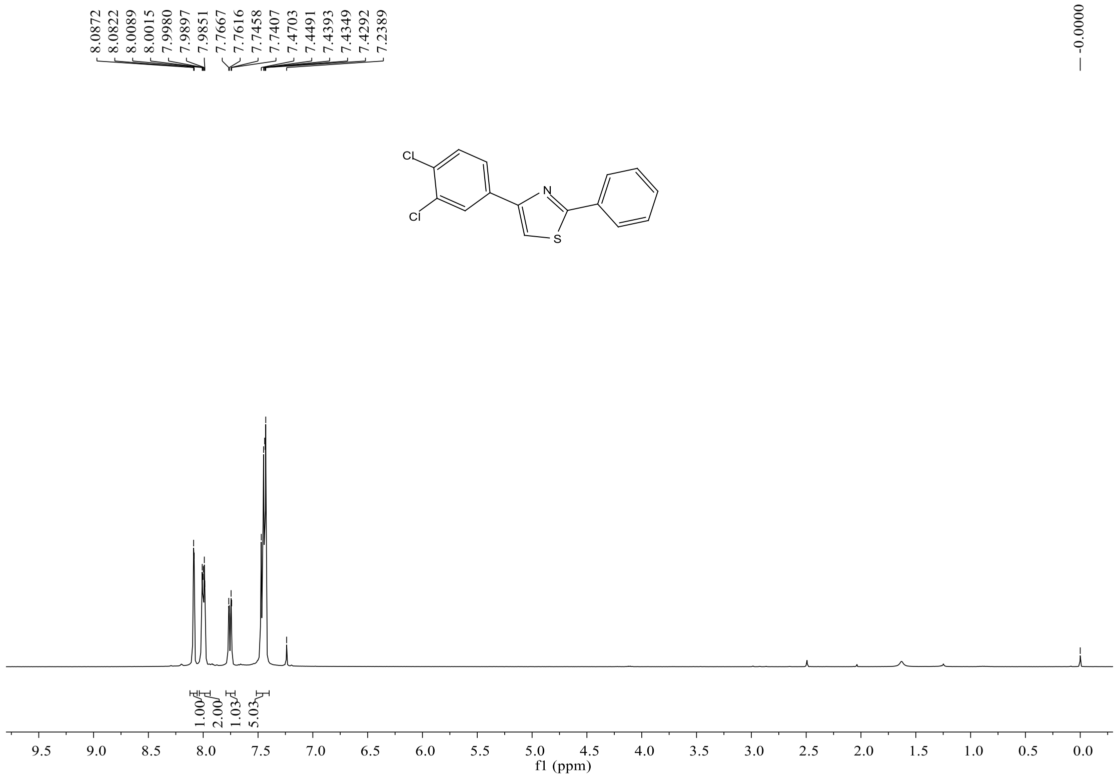

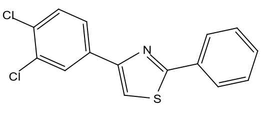

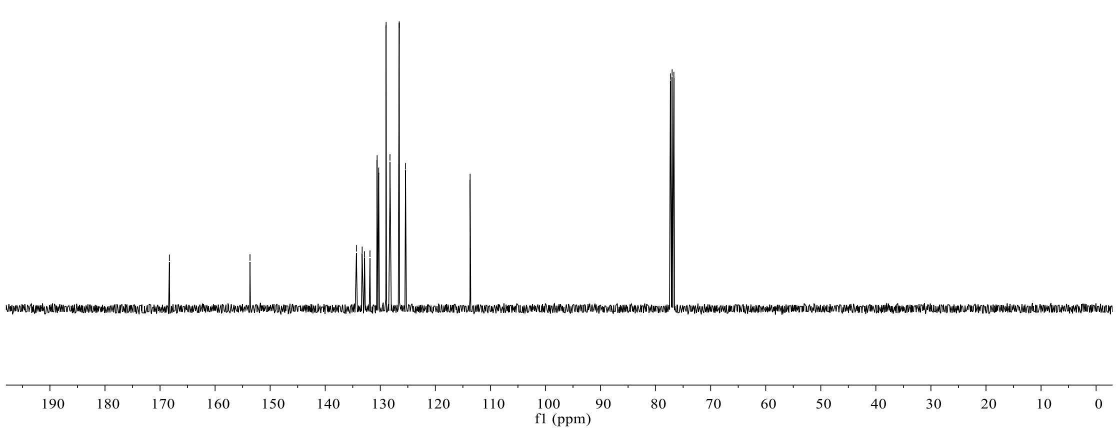

^{1}H and ^{13}C NMR spectra of **3az**

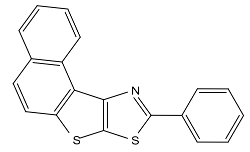

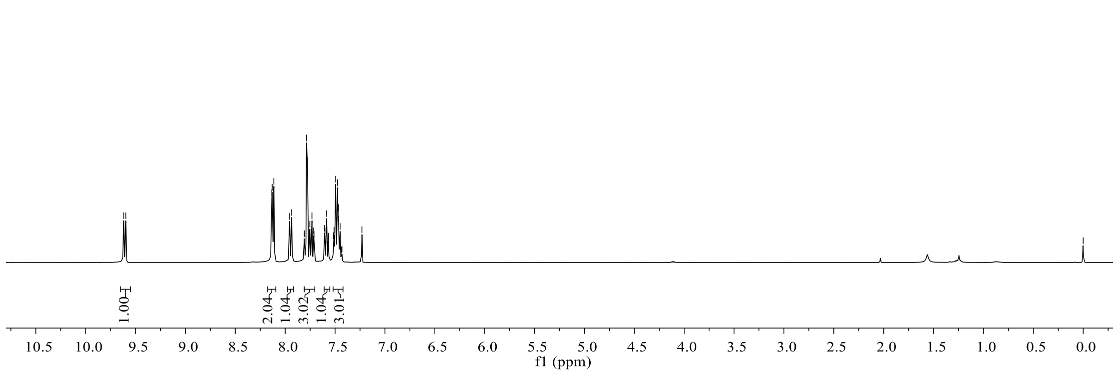

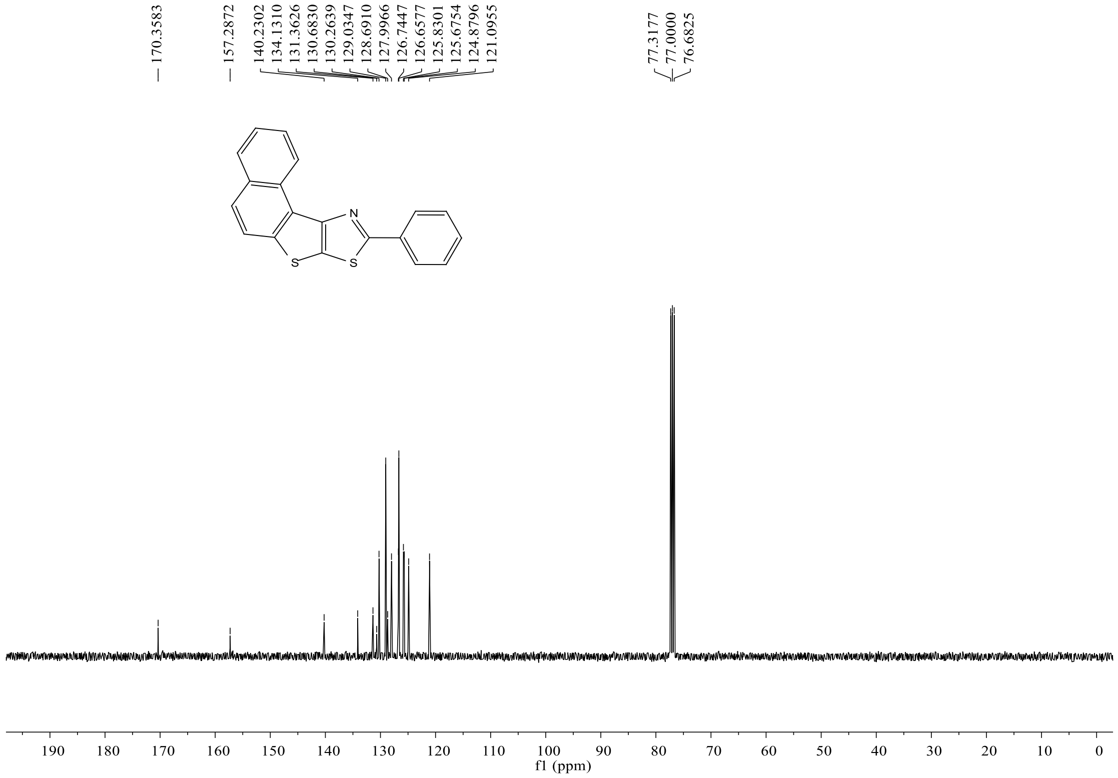

^{1}H and ^{13}C NMR spectra of **3aa'**

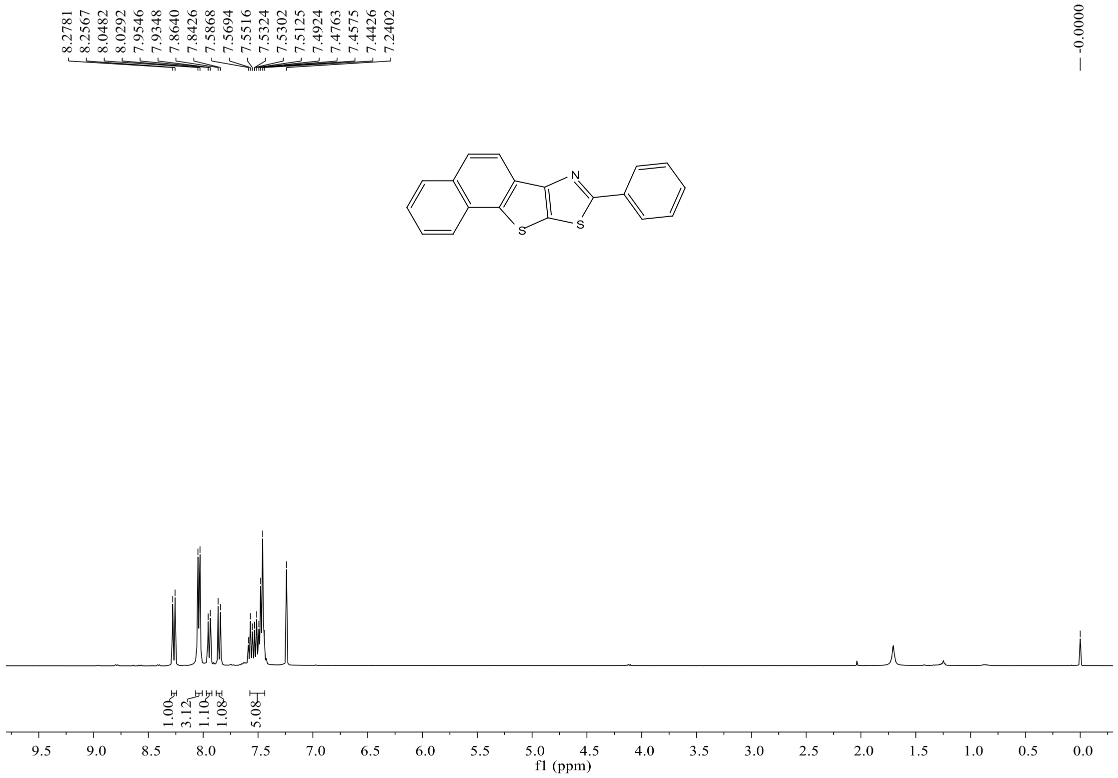

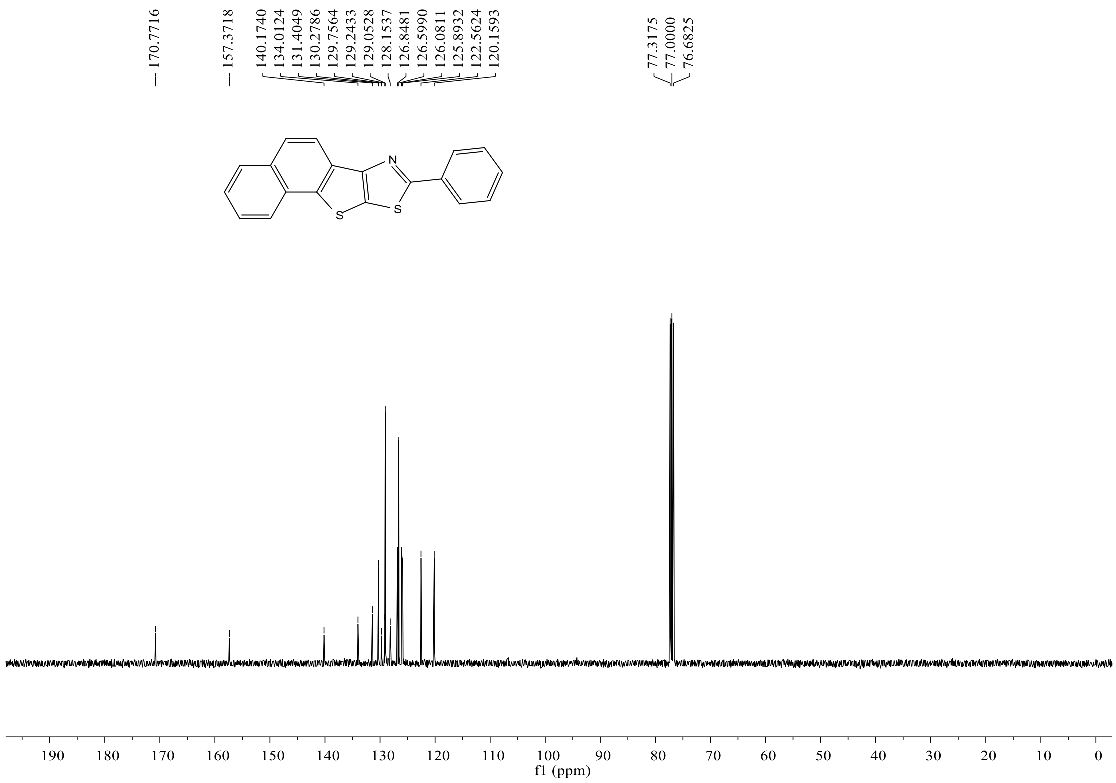

^{1}H and ^{13}C NMR spectra of **3ab'**

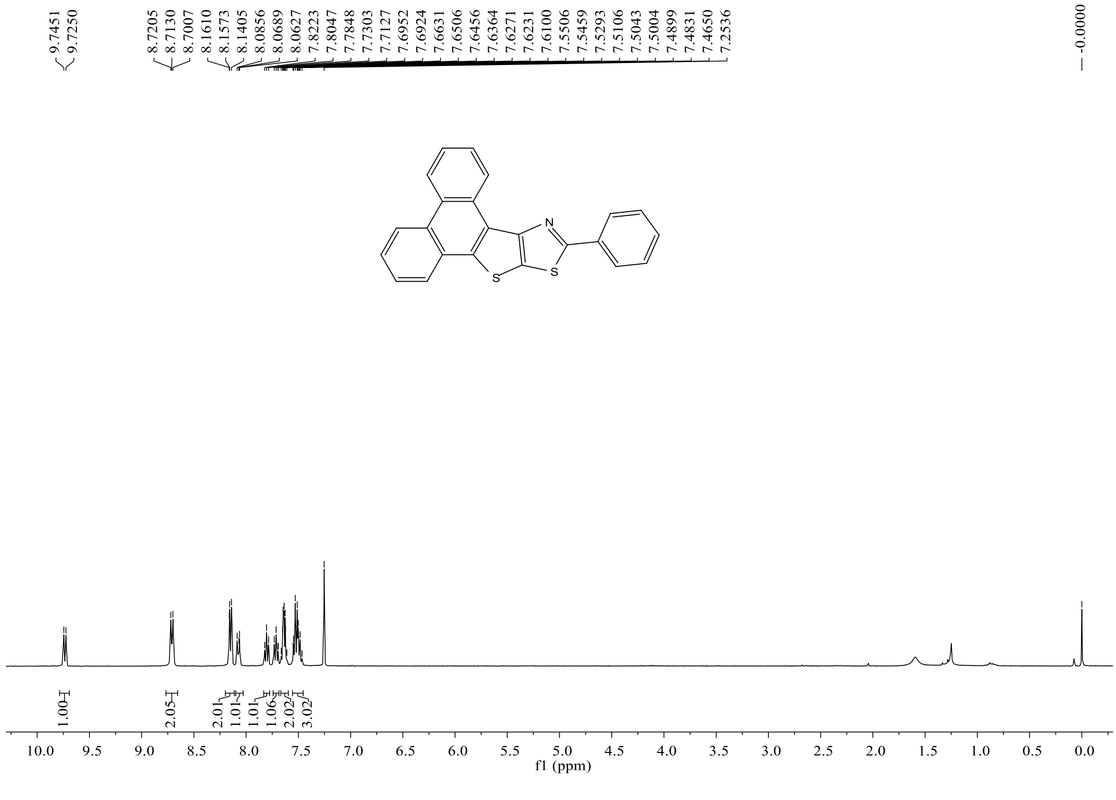

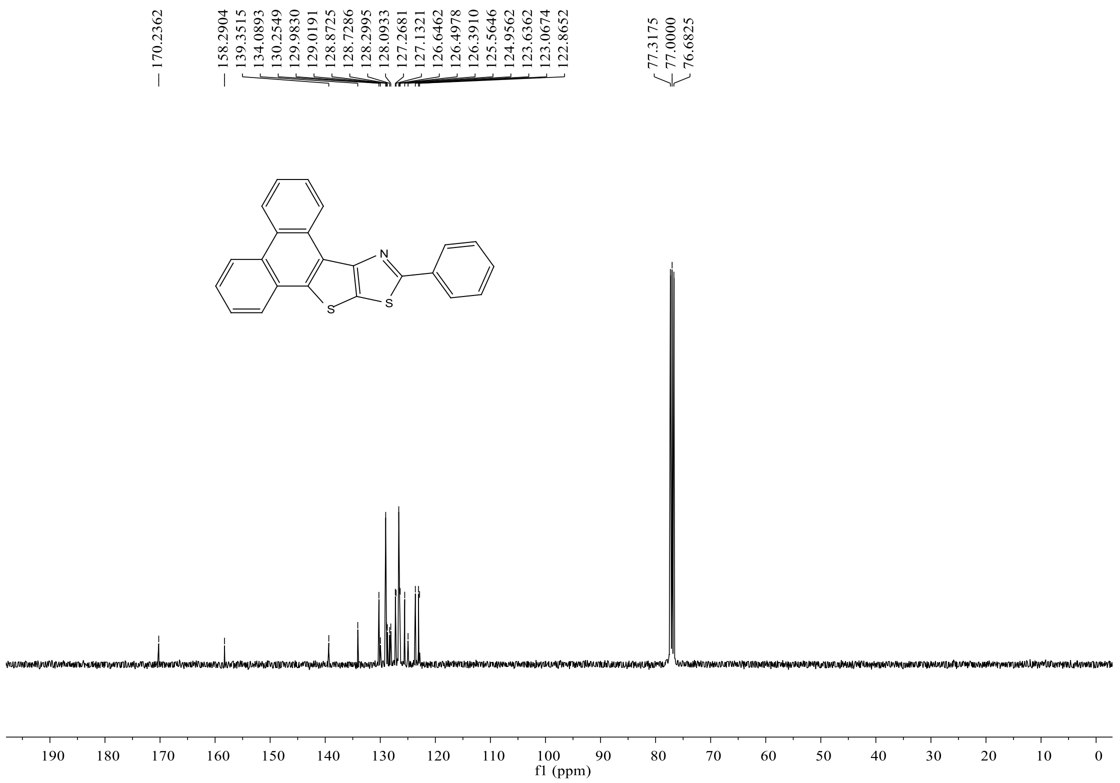

^{1}H and ^{13}C NMR spectra of **3ac’**

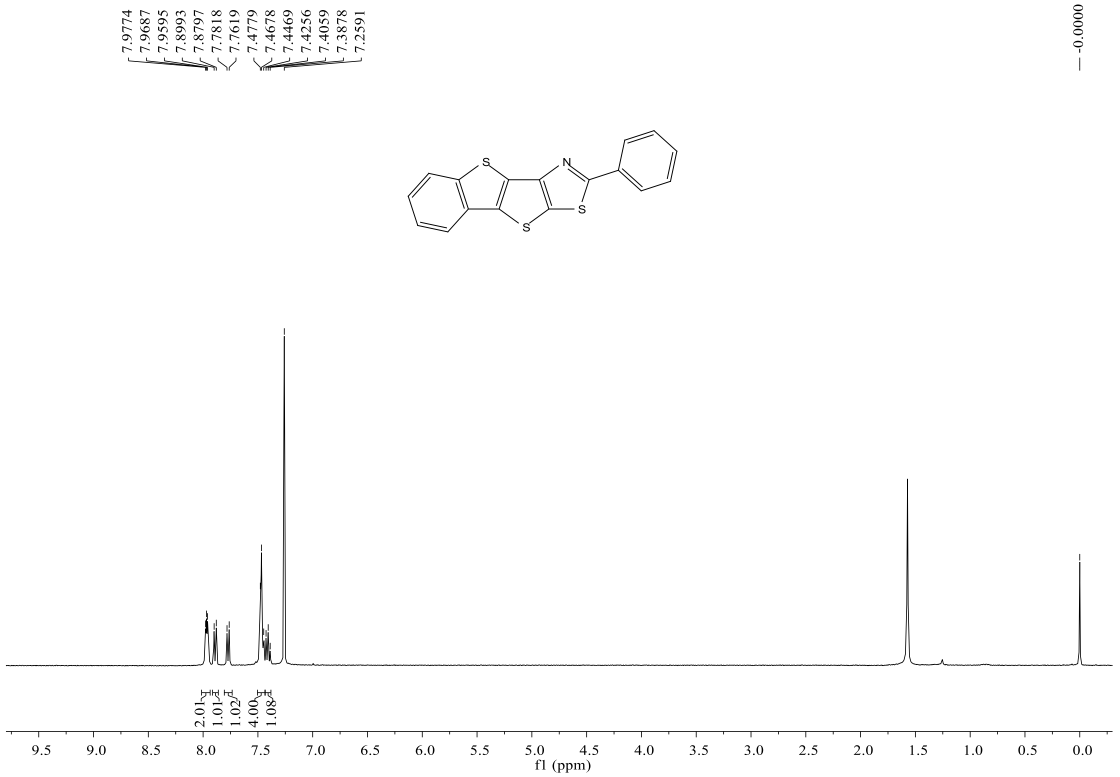

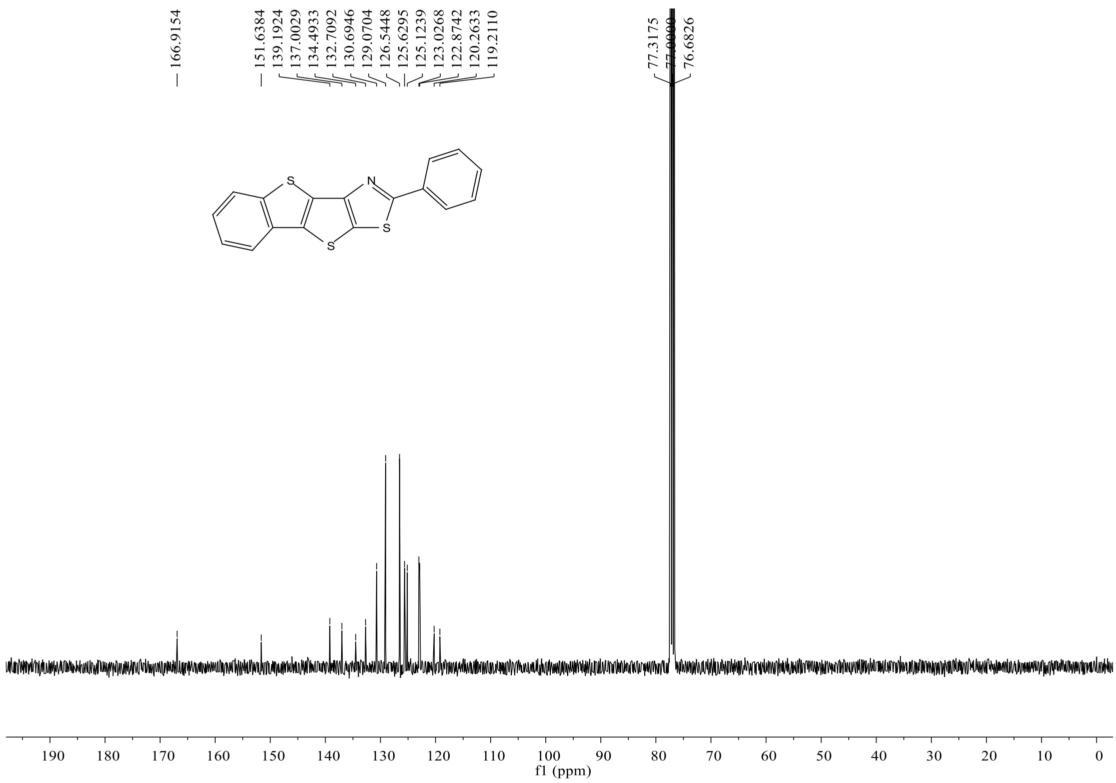

^{1}H and ^{13}C NMR spectra of **3ba**

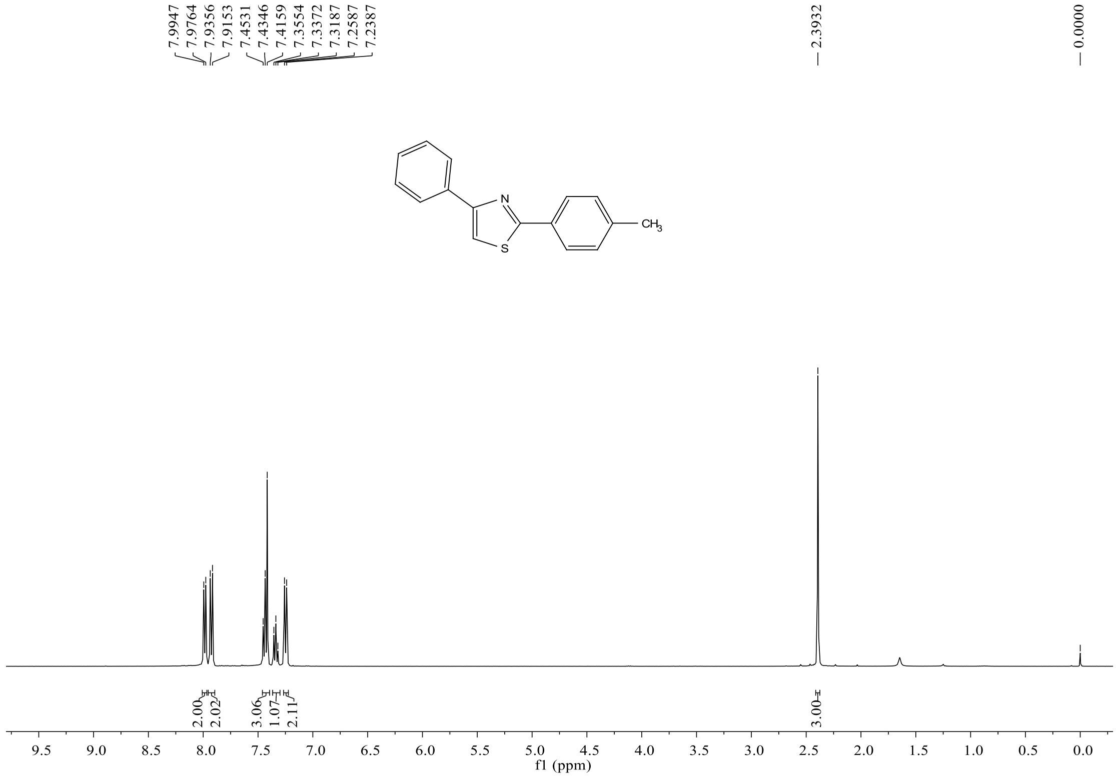

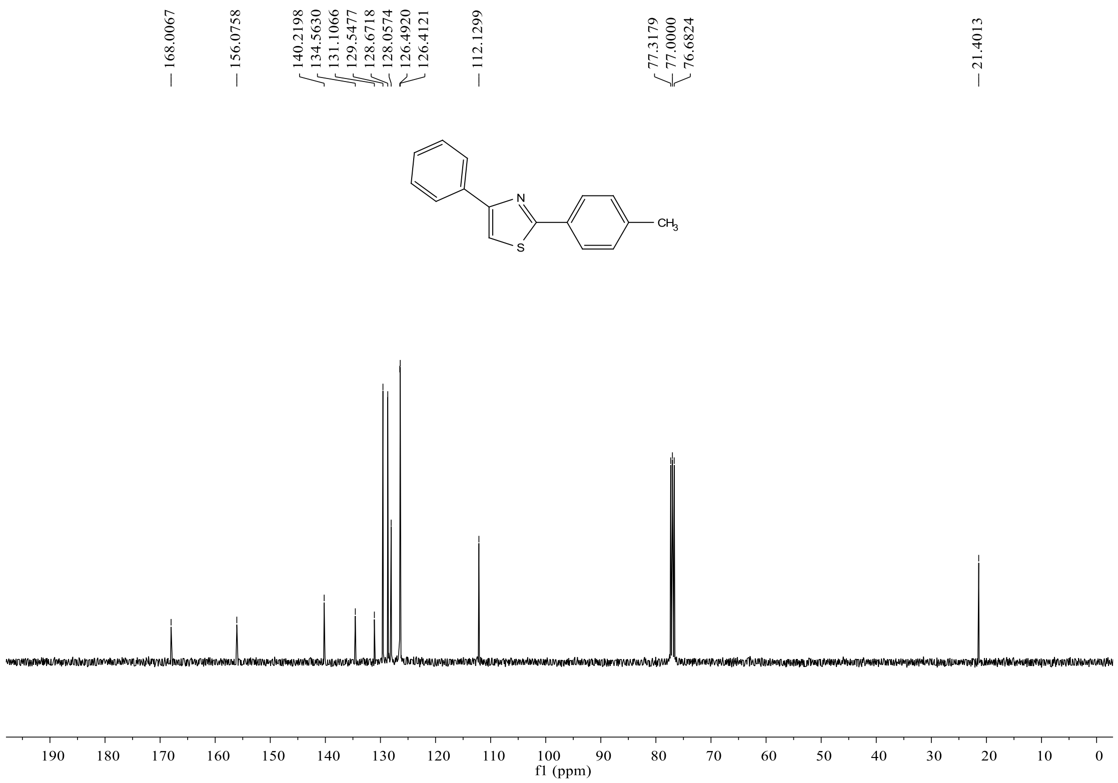

^{1}H, ^{13}C and ^{19}F NMR spectra of **3ca**

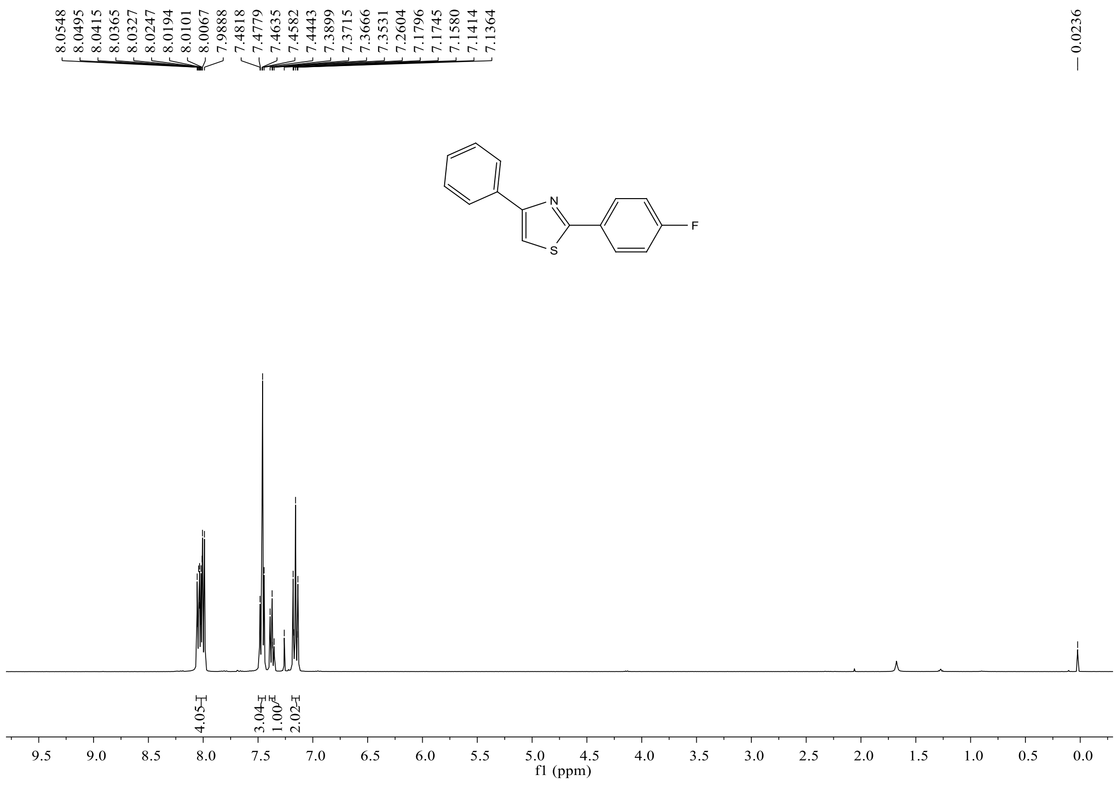

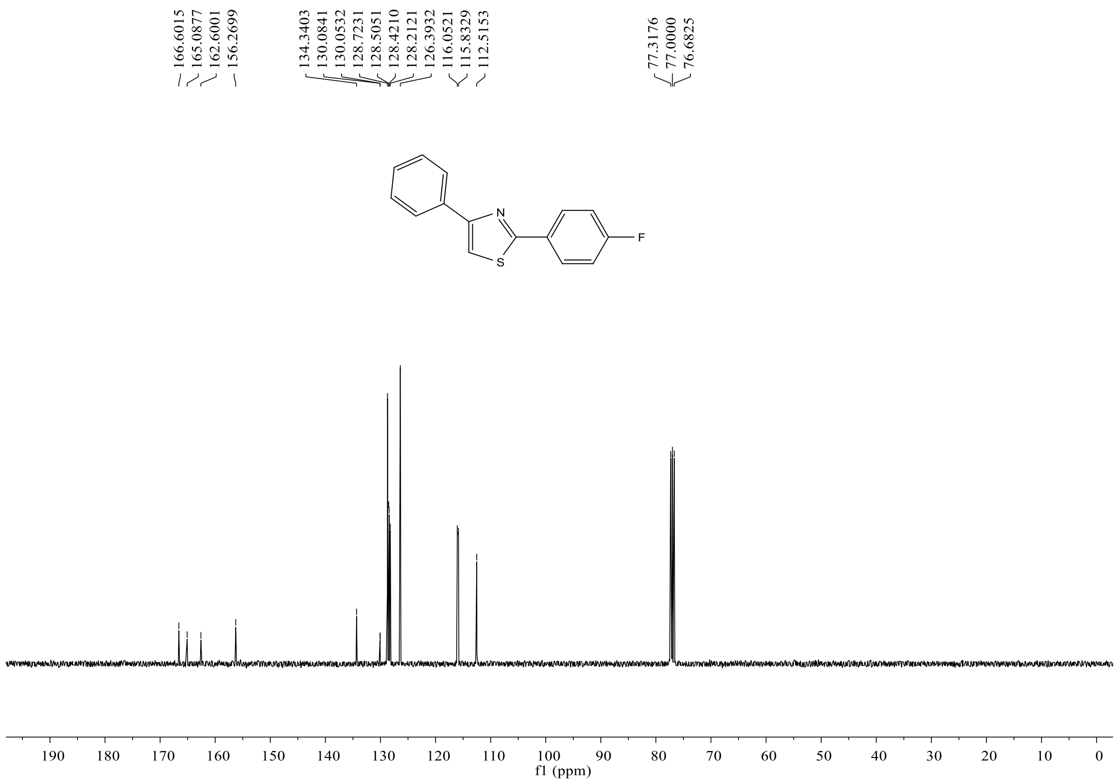
166.6015
165.0877
162.6001
156.2699
134.3403
130.0841
130.0532
128.7231
128.5051
128.4210
128.2121
126.3932
116.0521
115.8329
112.5153
77.3176
77.0000
76.6825
N
S
F
190 180 170 160 150 140 130 120 110 100 90 80 70 60 50 40 30 20 10 0
f1 (ppm)

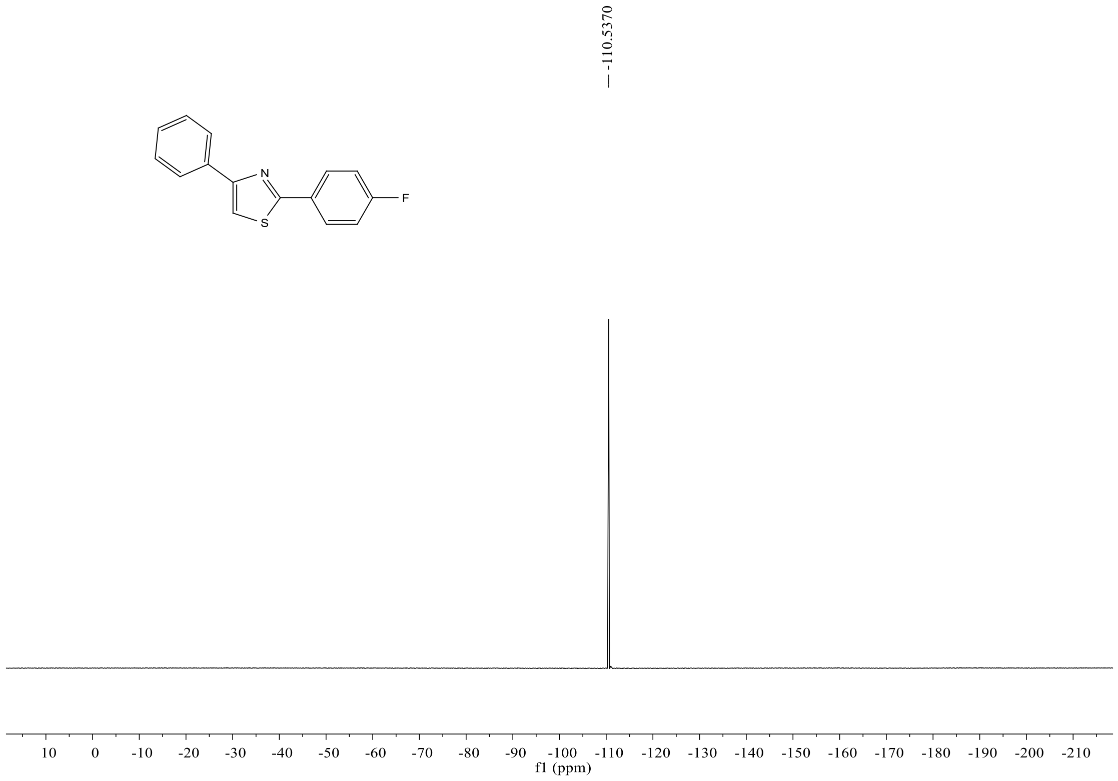
-110.5370
N
S
F
10 0 -10 -20 -30 -40 -50 -60 -70 -80 -90 -100 -110 -120 -130 -140 -150 -160 -170 -180 -190 -200 -210
f1 (ppm)

^{1}H and ^{13}C NMR spectra of **3da**

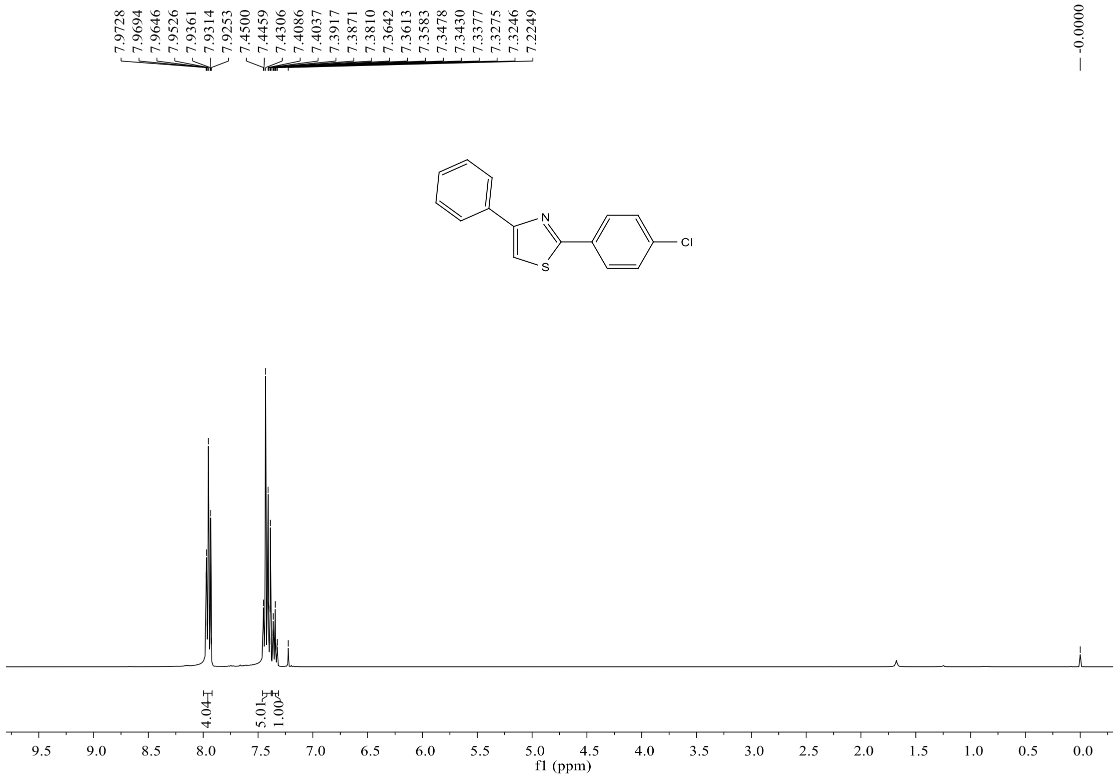

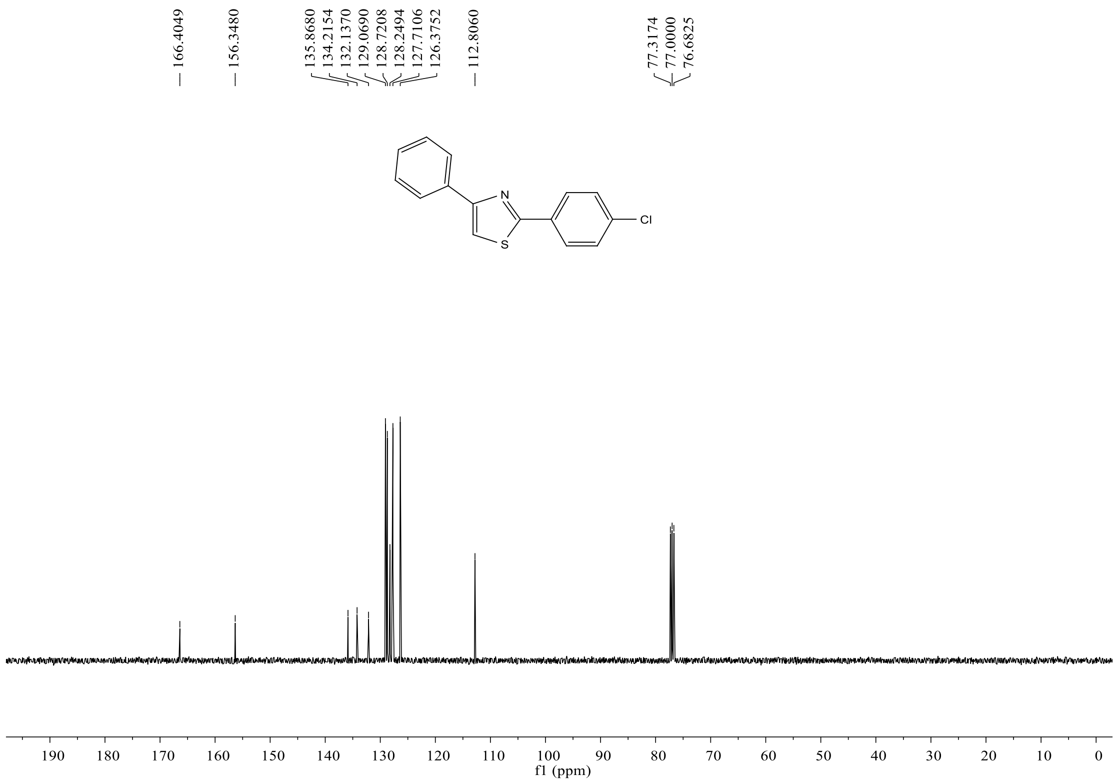

^{1}H and ^{13}C NMR spectra of **3ea**

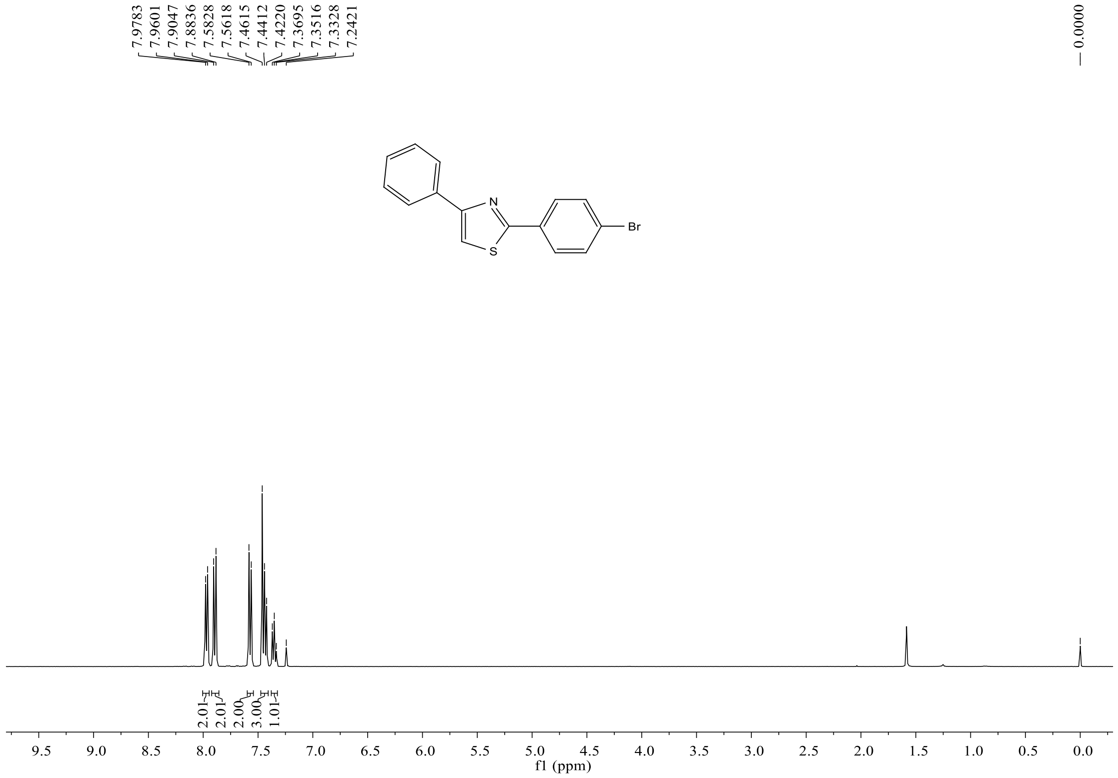

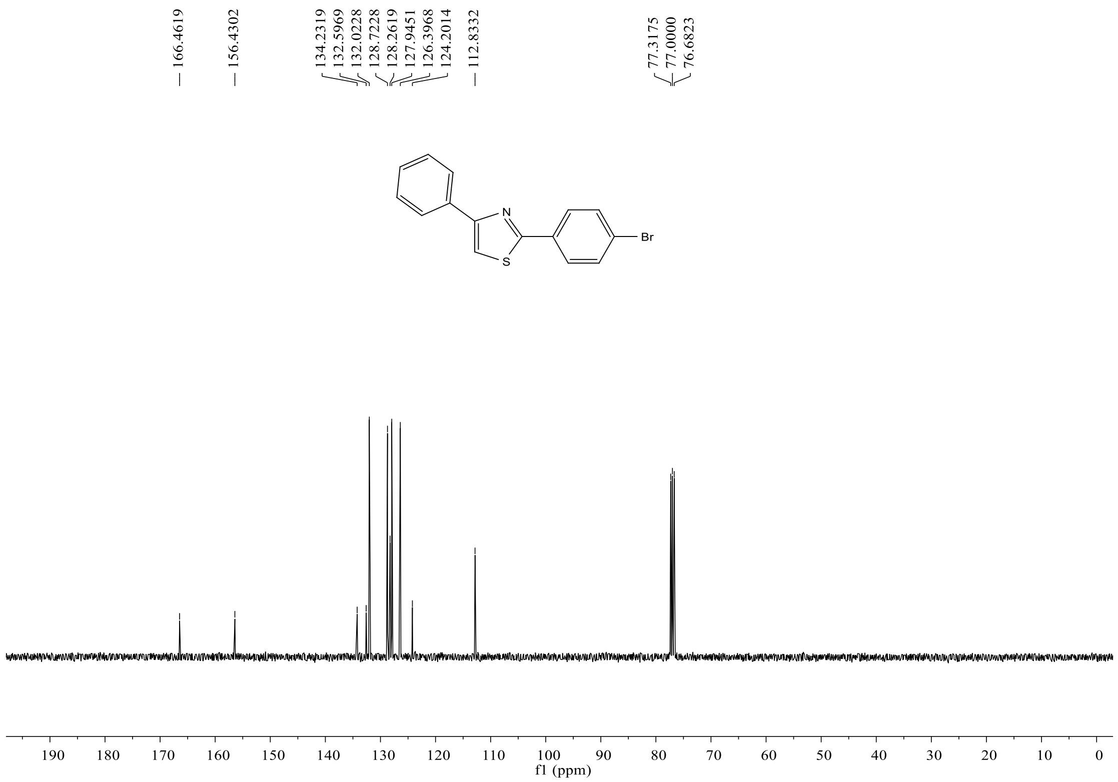

^{1}H, ^{13}C and ^{19}F NMR spectra of **3fa**

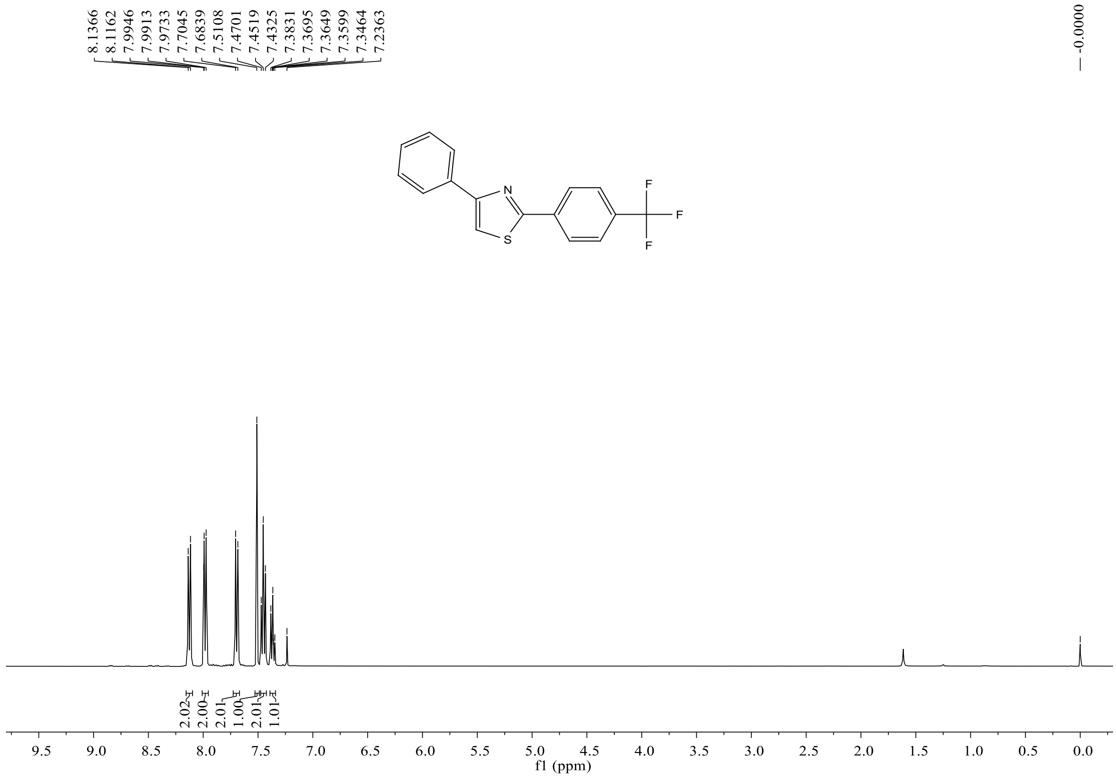

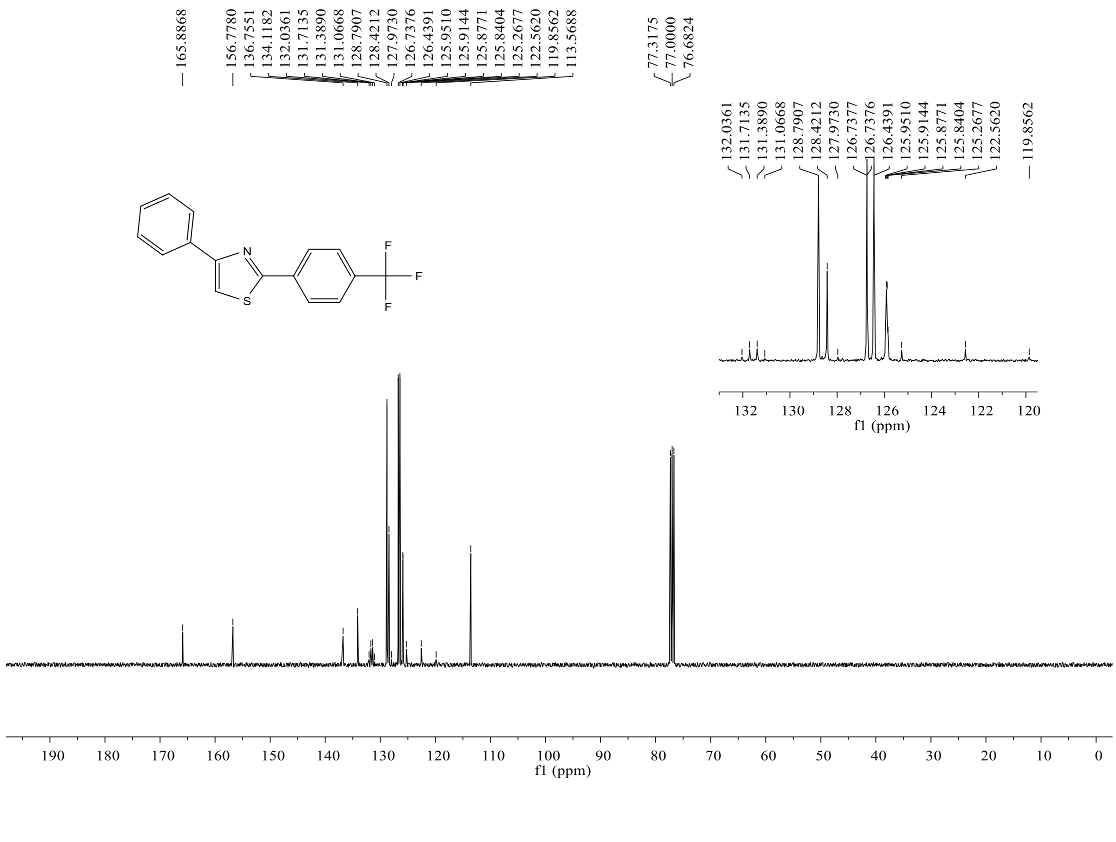

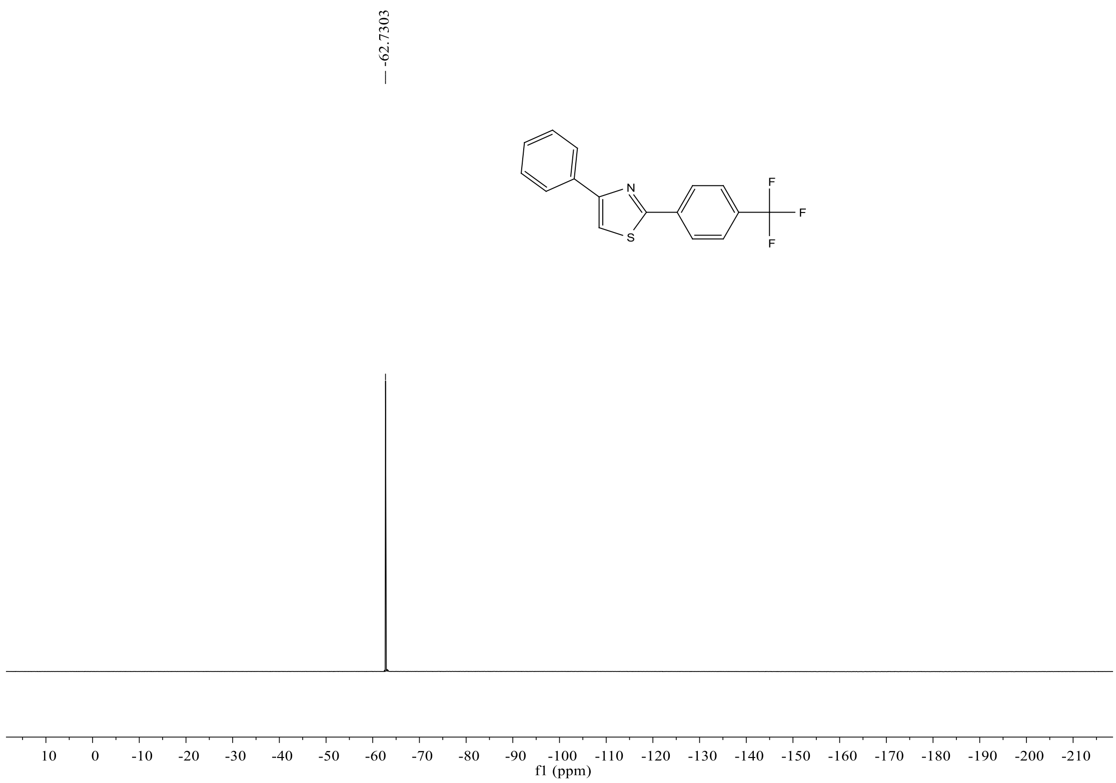

^{1}H, ^{13}C and ^{19}F NMR spectra of **3ga**

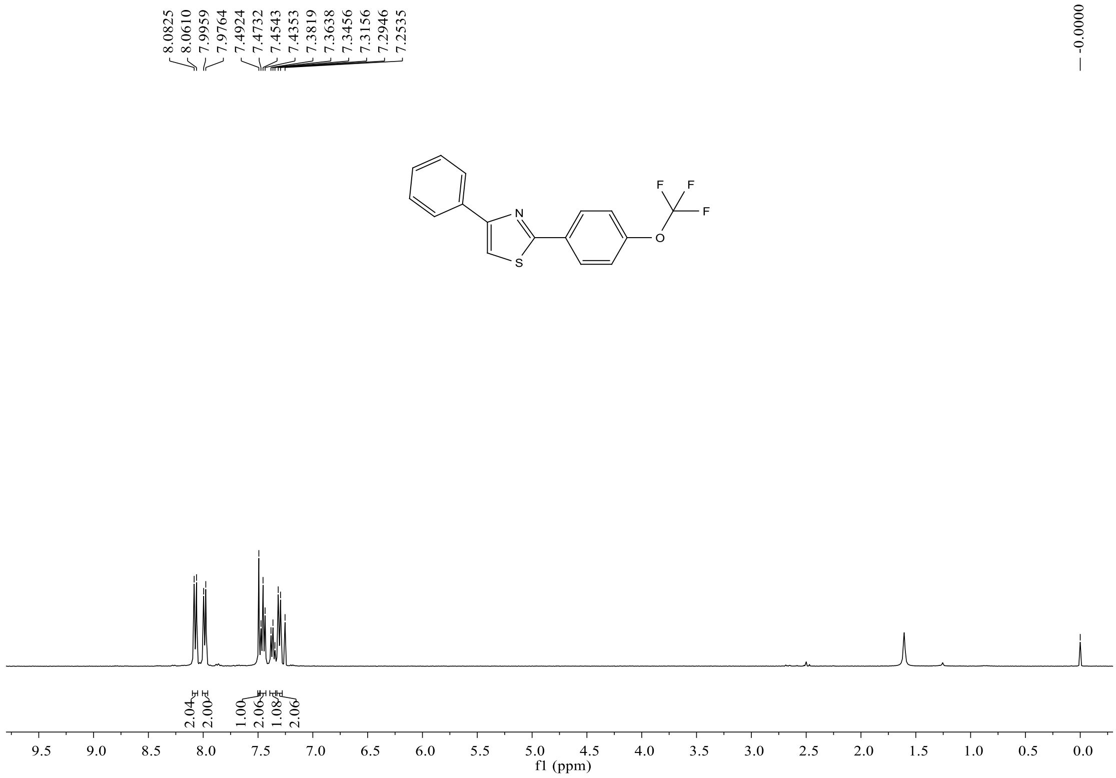

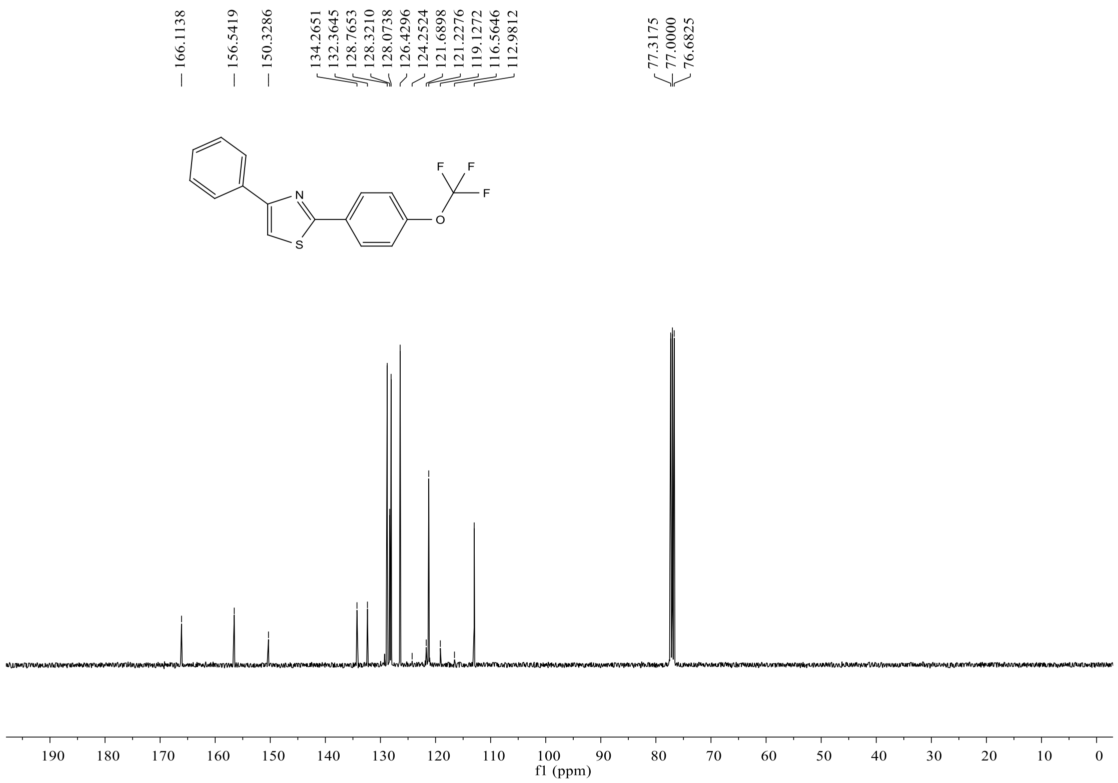
166.1138
156.5419
150.3286
134.2651
132.3645
128.7653
128.3210
128.0738
126.4296
124.2524
121.6898
121.2276
119.1272
116.5646
112.9812
77.3175
77.0000
76.6825
N
S
O
F
F
F
190 180 170 160 150 140 130 120 110 100 90 80 70 60 50 40 30 20 10 0
f1 (ppm)

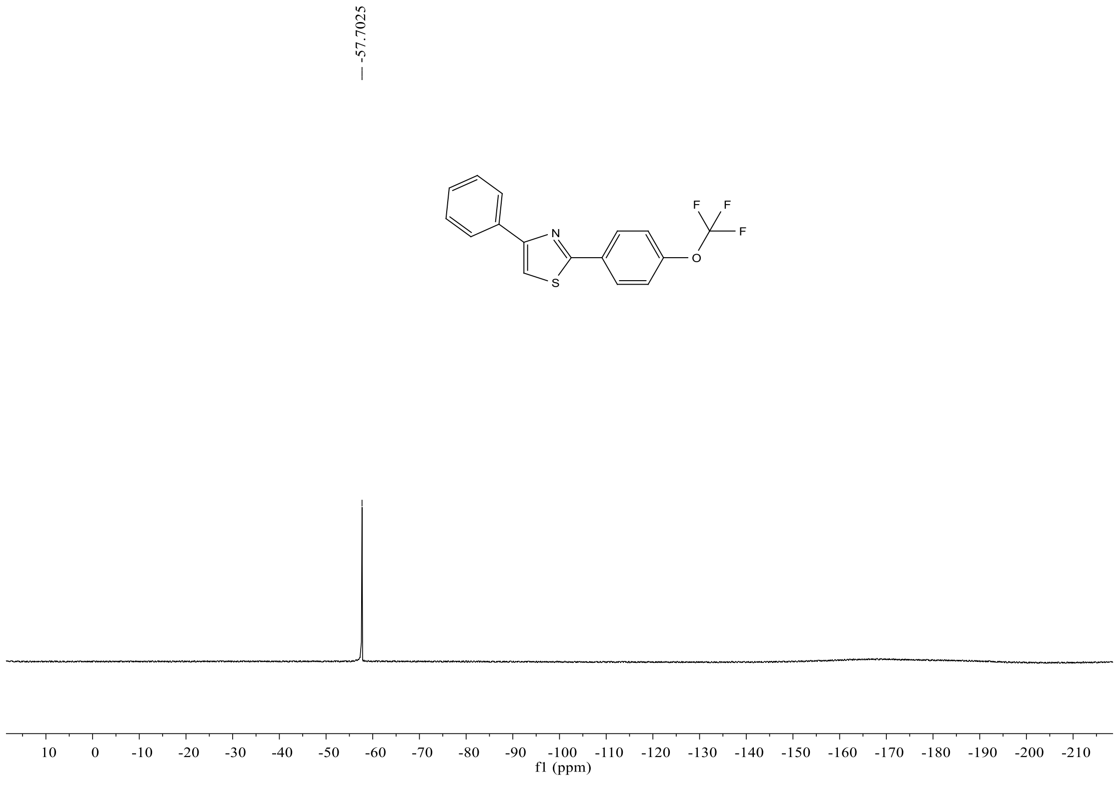
-57.7025
N
S
O
F
F
F
10 0 -10 -20 -30 -40 -50 -60 -70 -80 -90 -100 -110 -120 -130 -140 -150 -160 -170 -180 -190 -200 -210
f1 (ppm)

^{1}H and ^{13}C NMR spectra of **3ha**

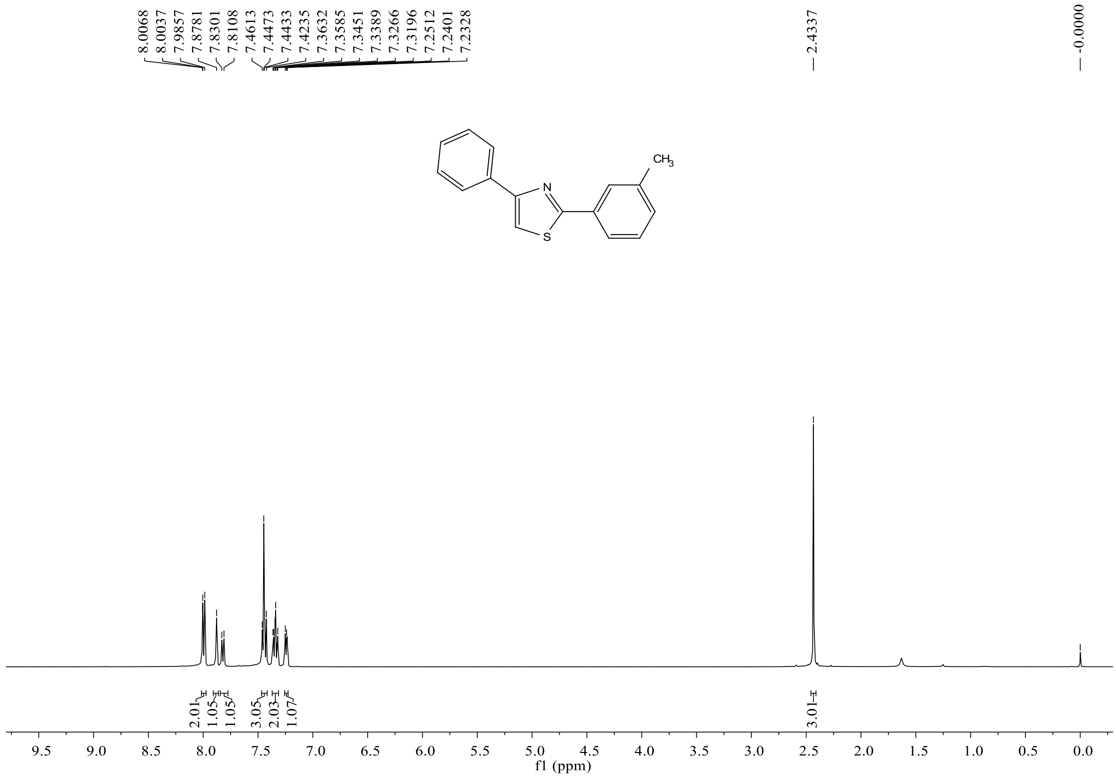

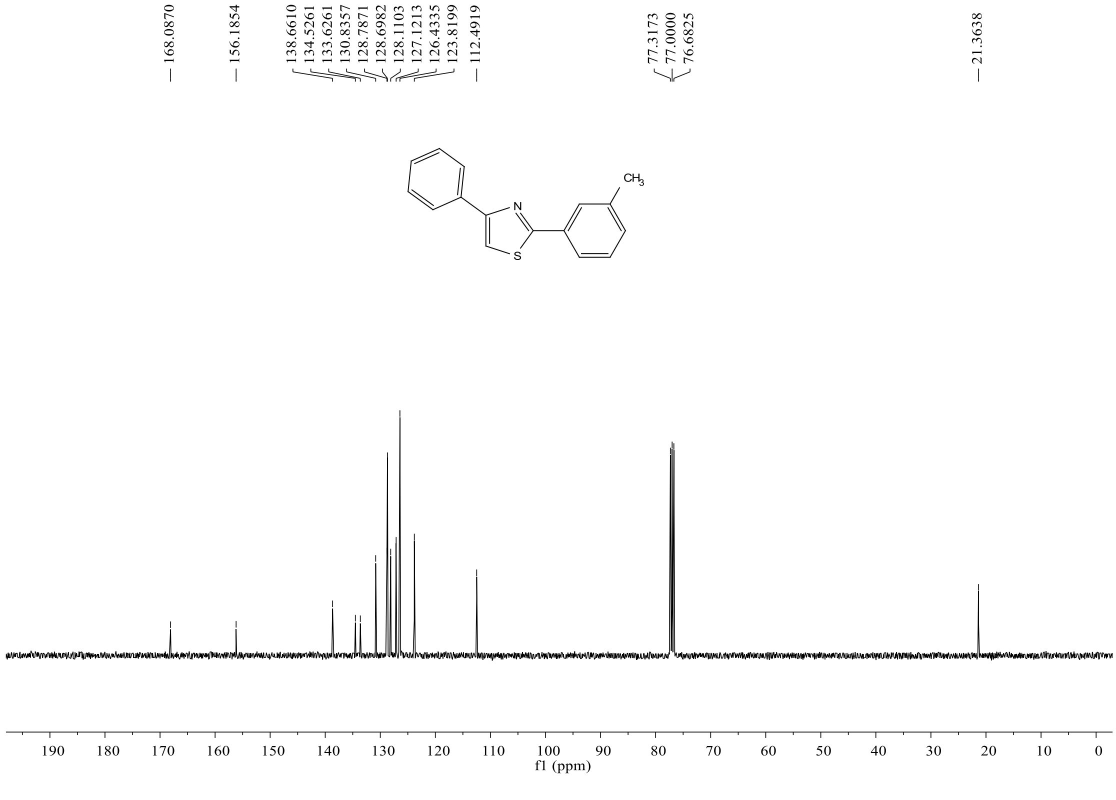

^{1}H, ^{13}C and ^{19}F NMR spectra of **3ia**

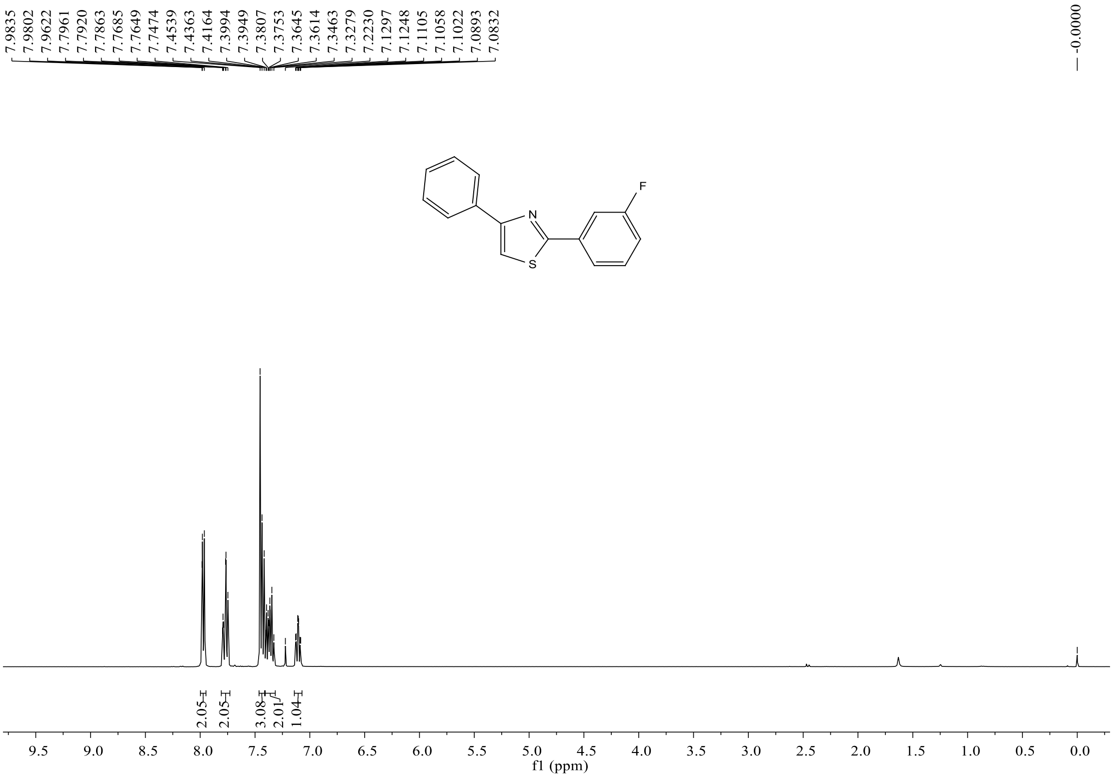

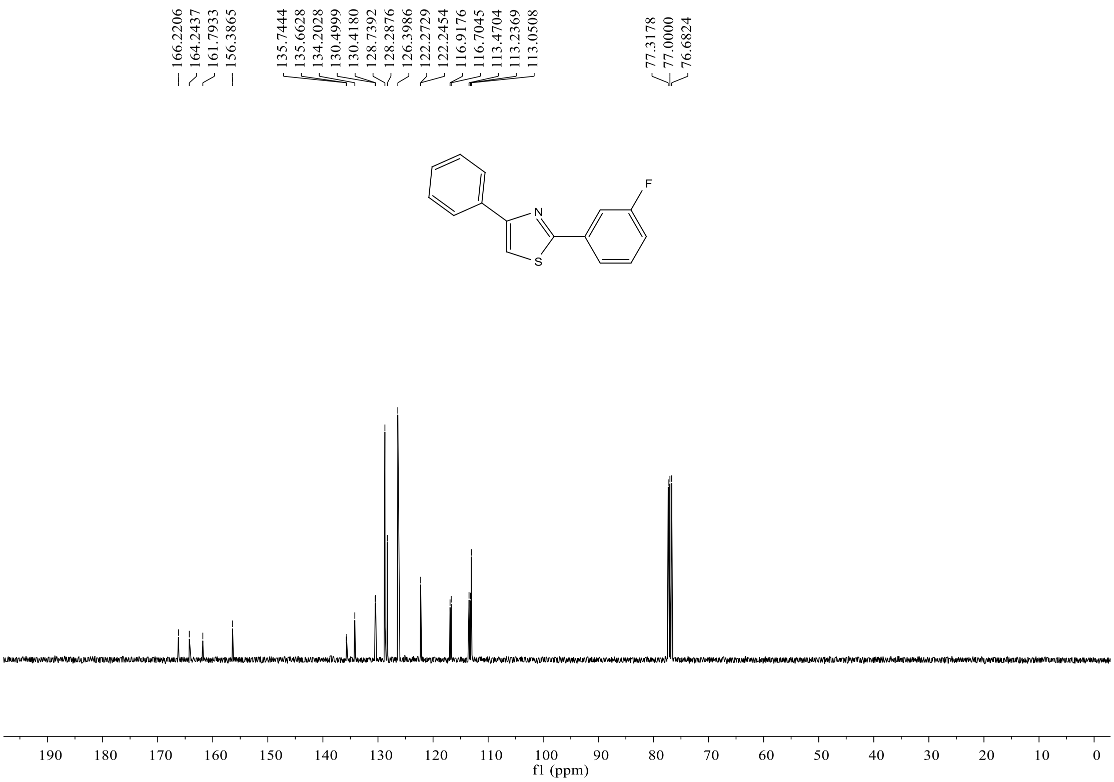

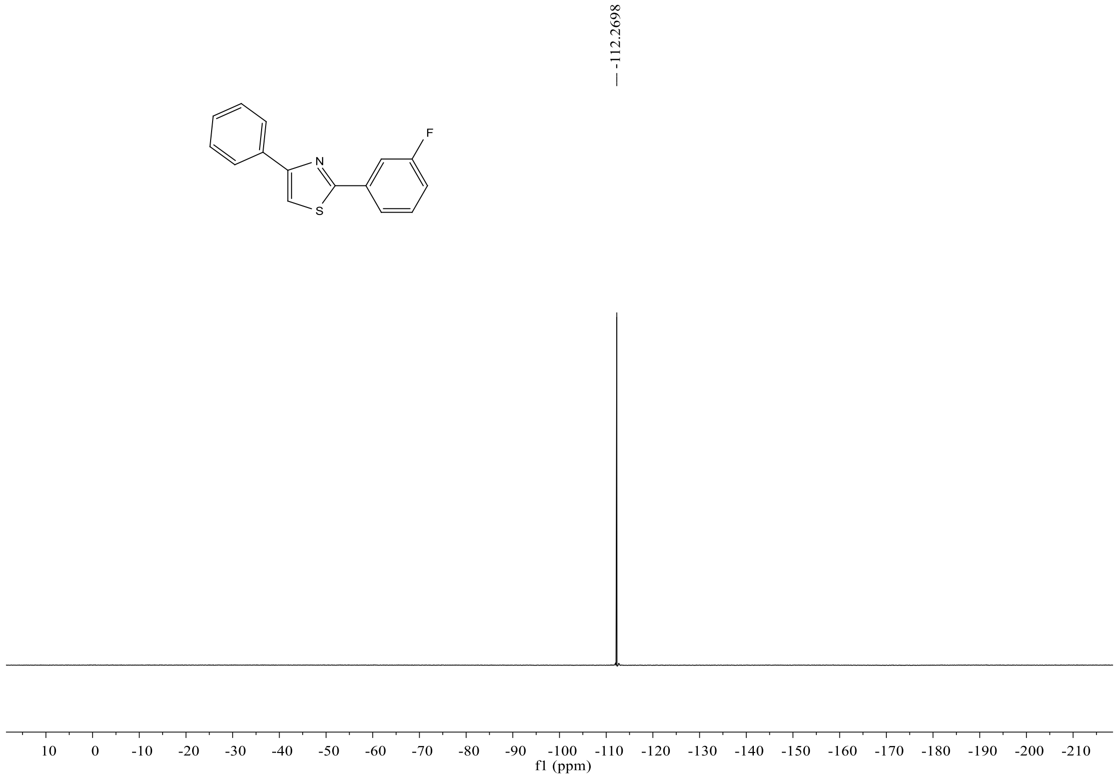

^{1}H and ^{13}C NMR spectra of **3ja**

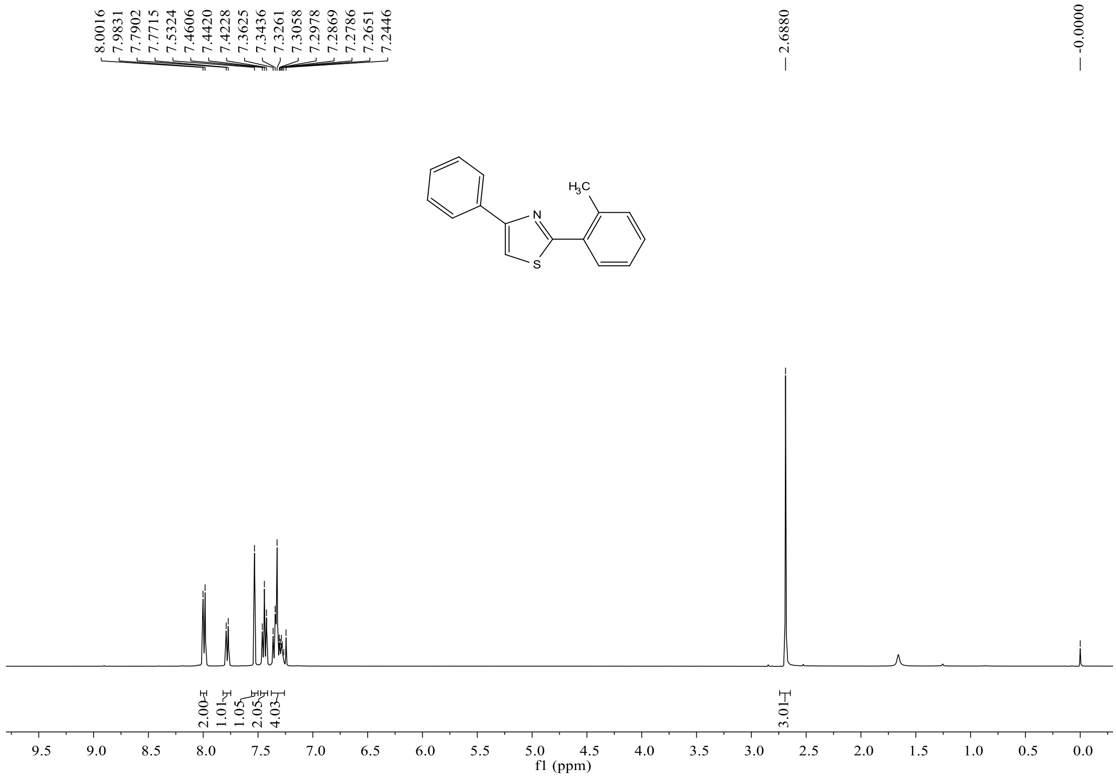

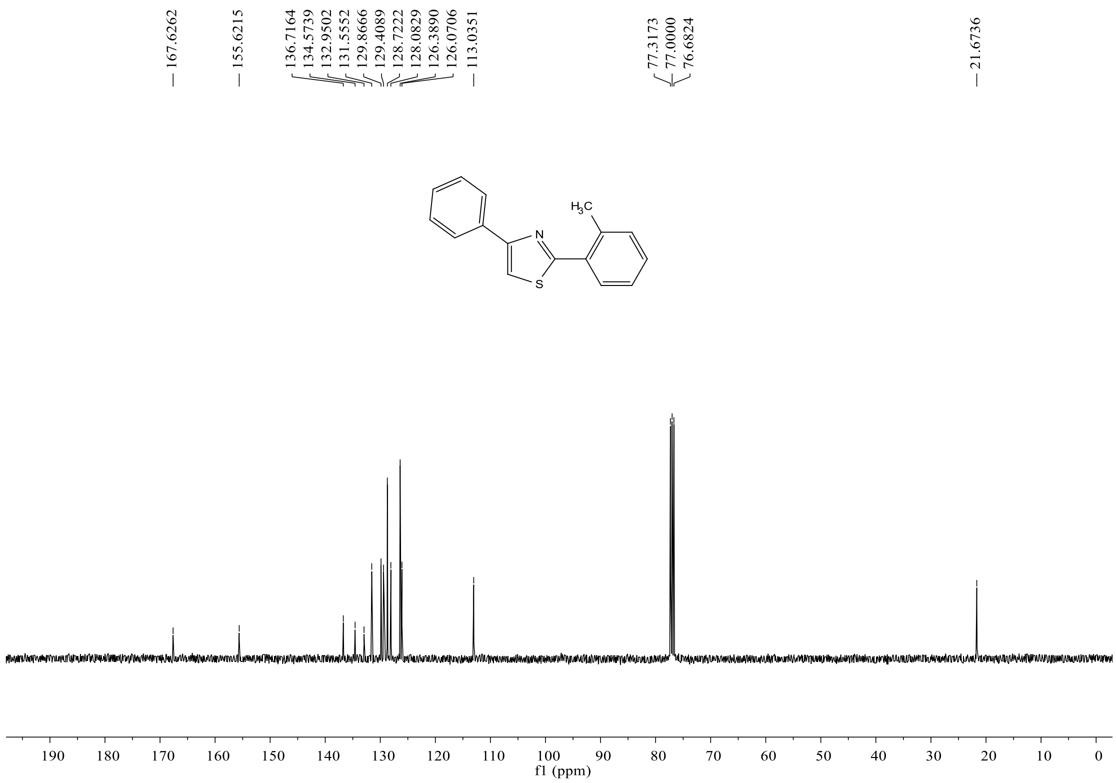

^{1}H and ^{13}C NMR spectra of **3ka**

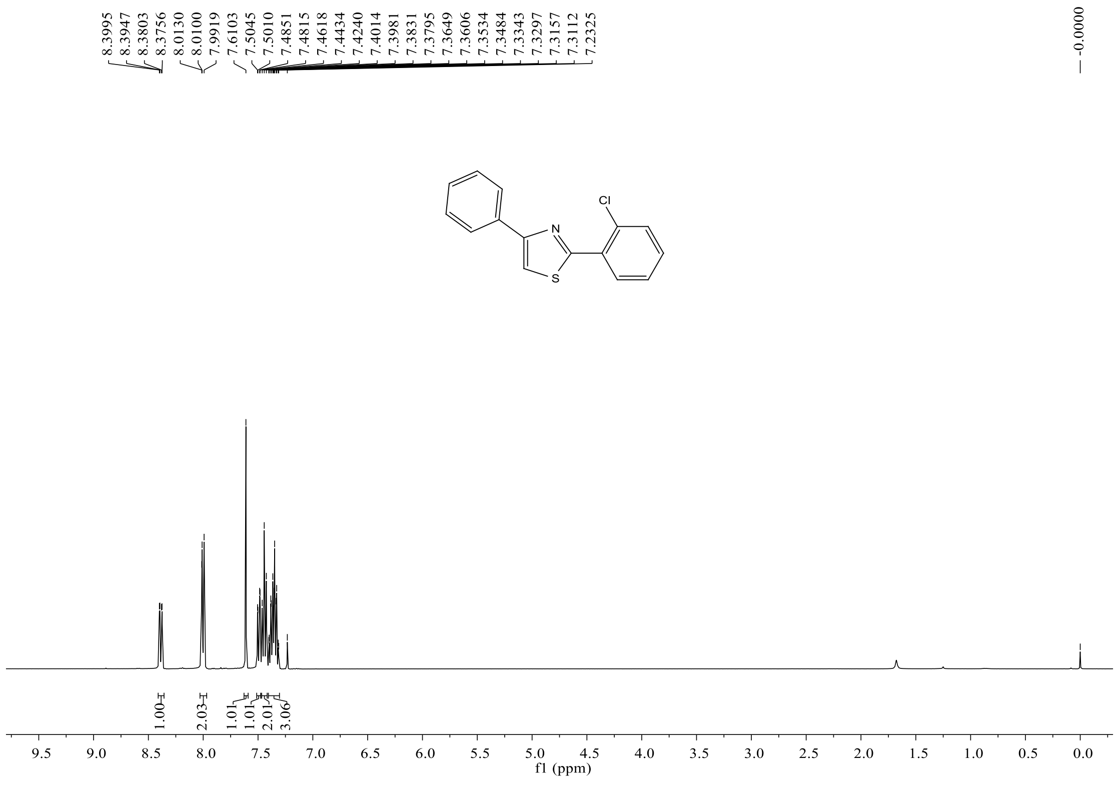

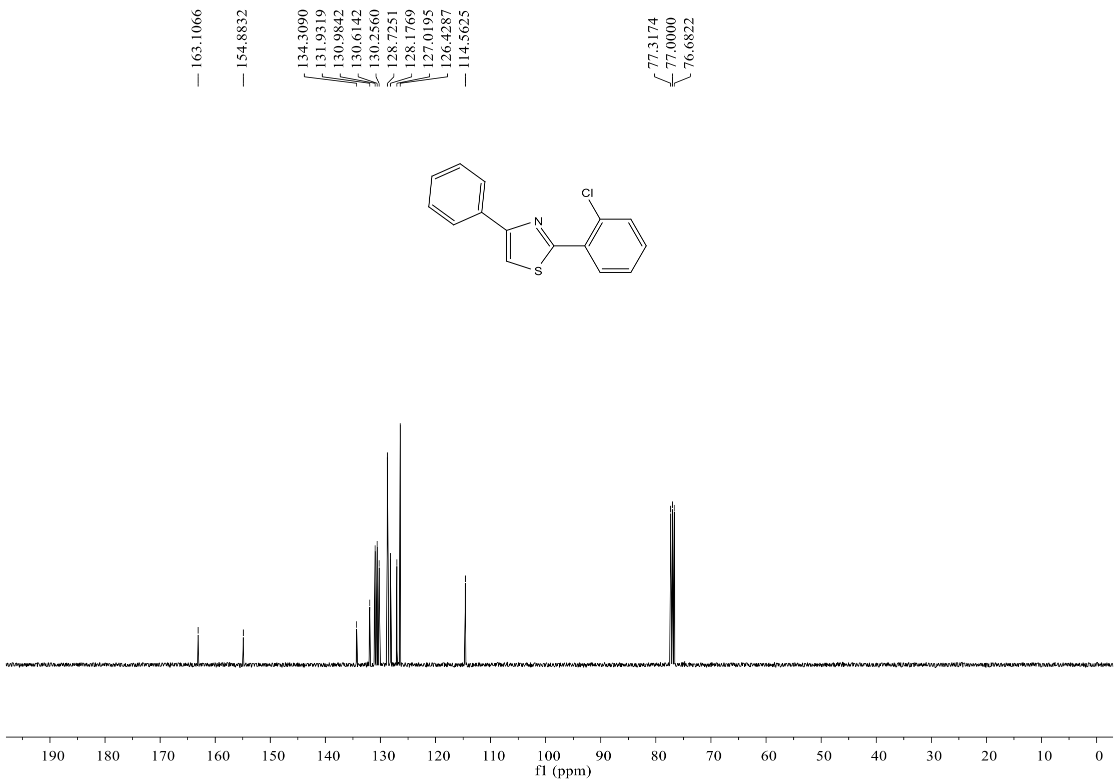

^{1}H, ^{13}C and ^{19}F NMR spectra of **3la**

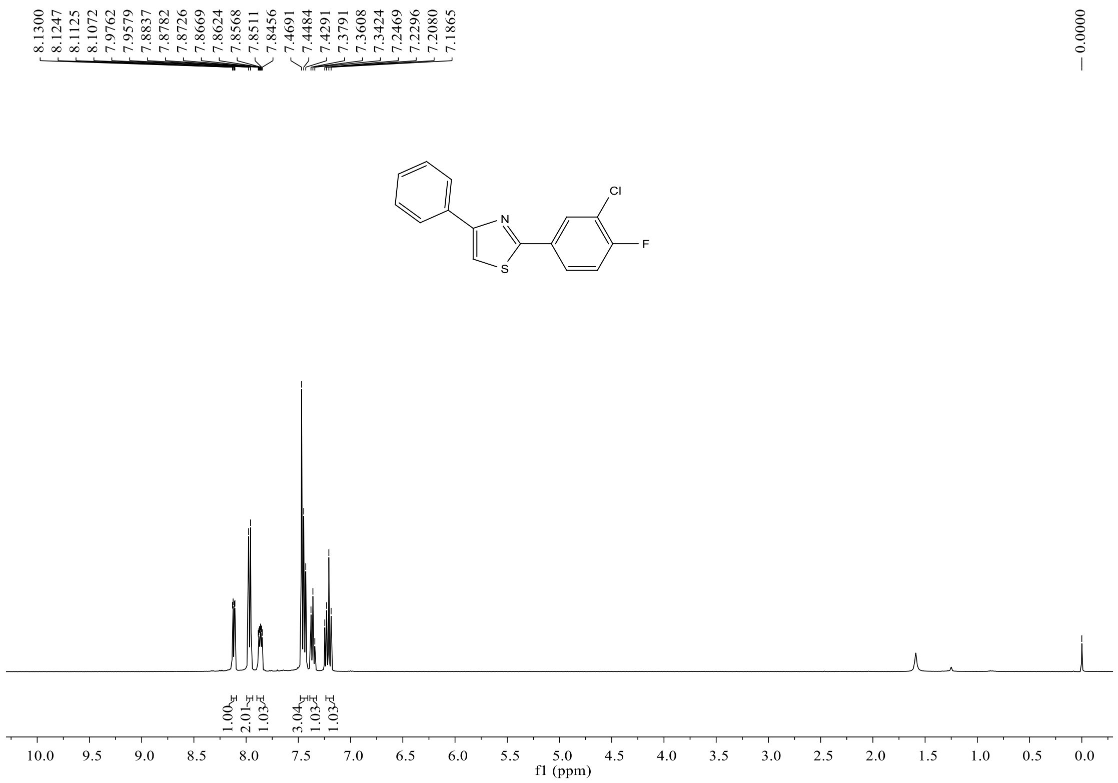

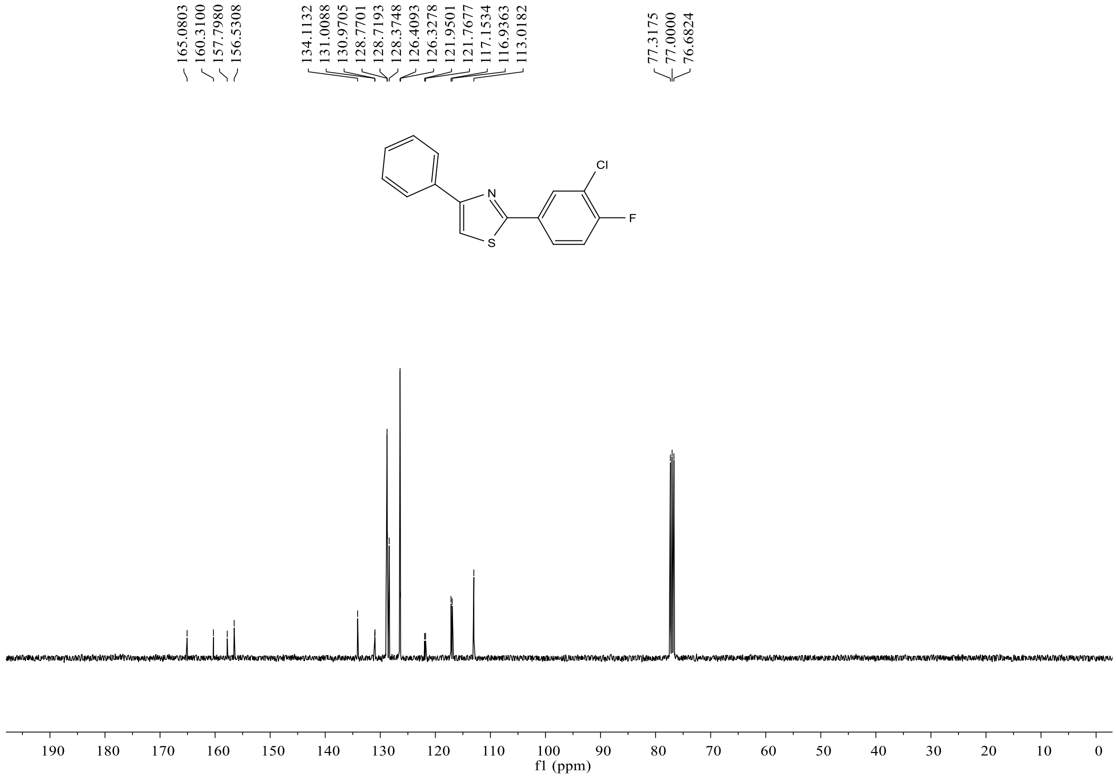
165.0803
160.3100
157.7980
156.5308
134.1132
131.0088
130.9705
128.7701
128.7193
128.3748
126.4093
126.3278
121.9501
121.7677
117.1534
116.9363
113.0182
77.3175
77.0000
76.6824
N
S
Cl
F
190 180 170 160 150 140 130 120 110 100 90 80 70 60 50 40 30 20 10 0
f1 (ppm)

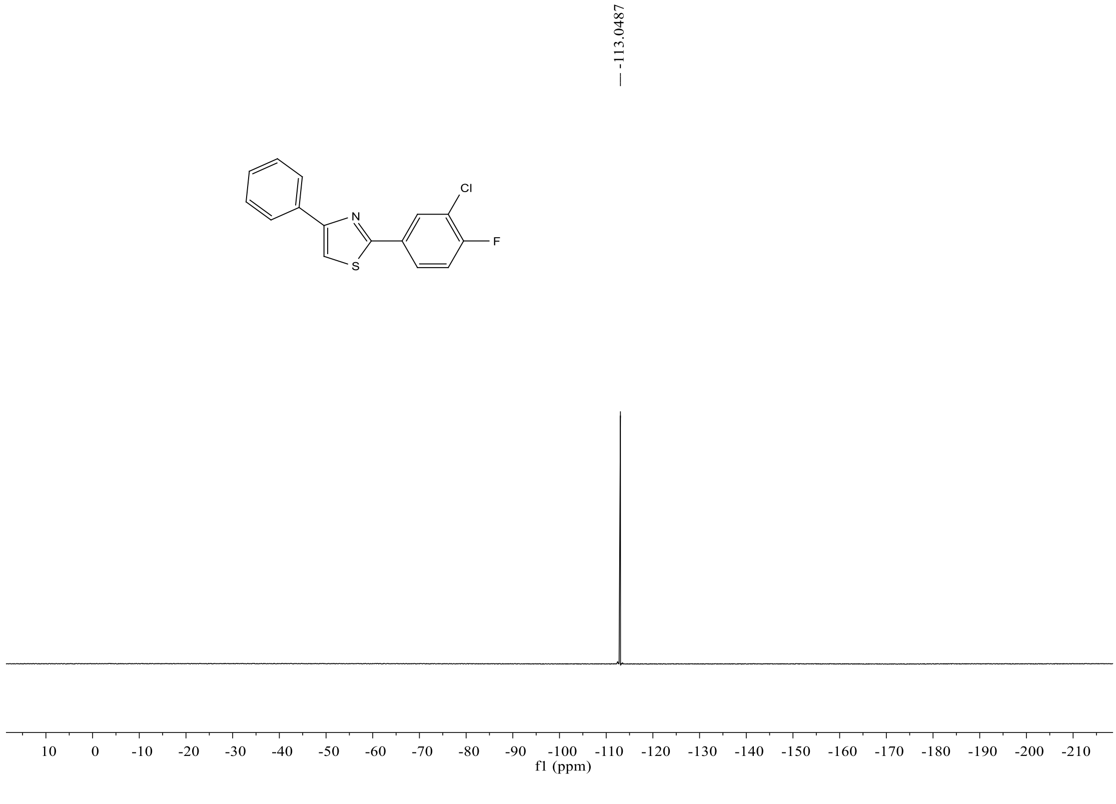
-113.0487
N
S
Cl
F
10 0 -10 -20 -30 -40 -50 -60 -70 -80 -90 -100 -110 -120 -130 -140 -150 -160 -170 -180 -190 -200 -210
f1 (ppm)

^{1}H, ^{13}C and ^{19}F NMR NMR spectra of **3ma**

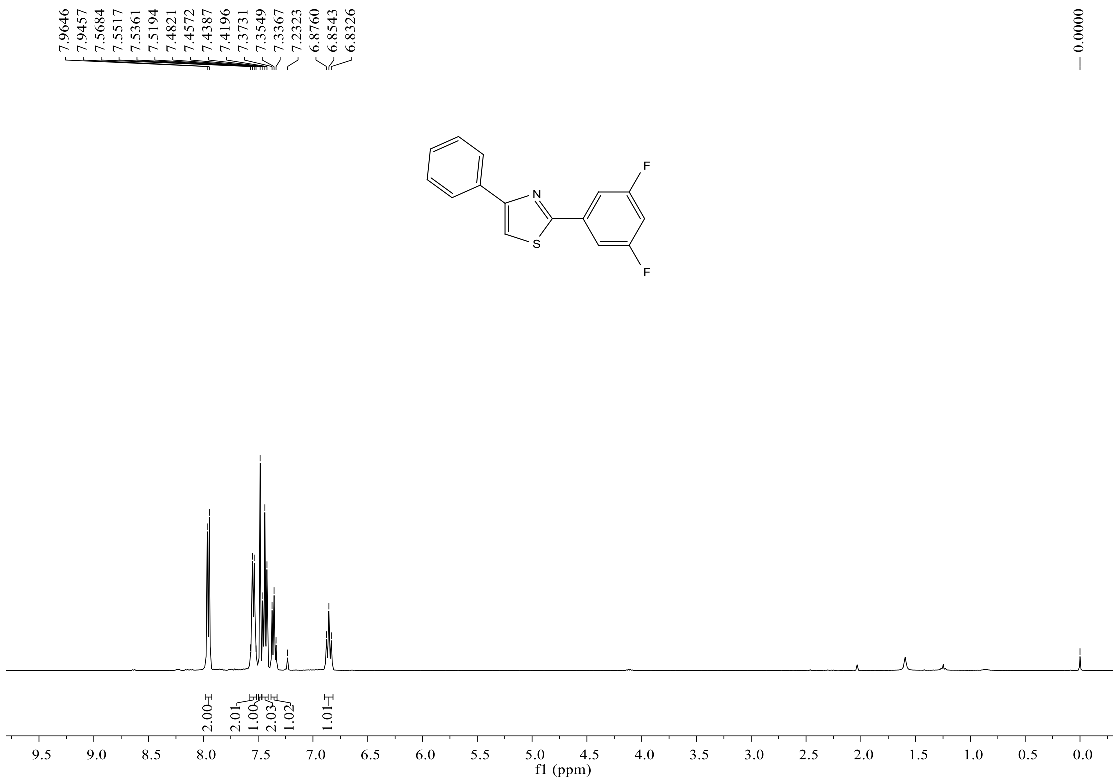

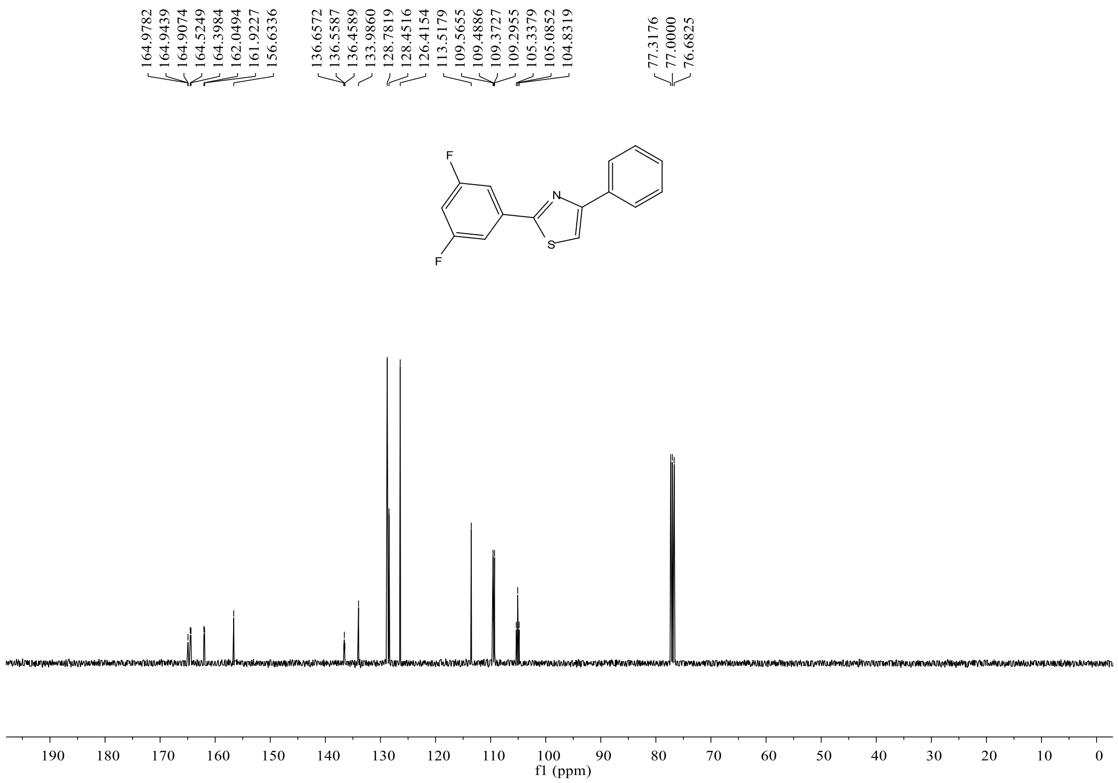

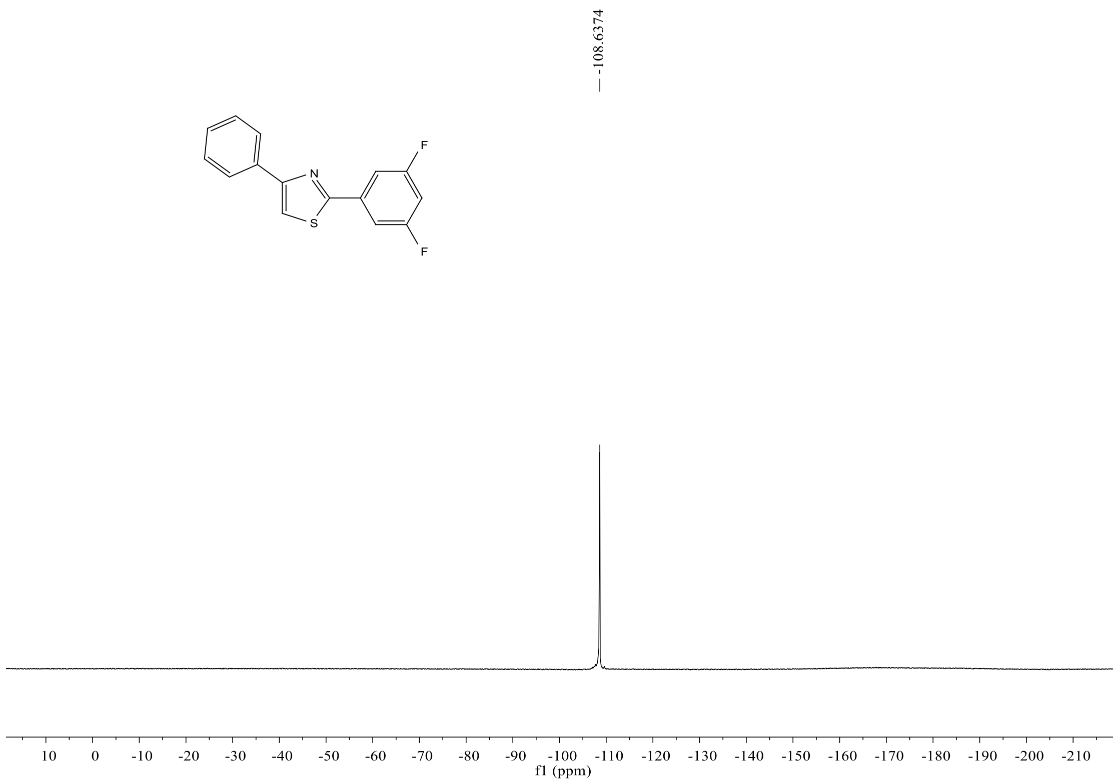

^{1}H and ^{13}C NMR NMR spectra of **3na**

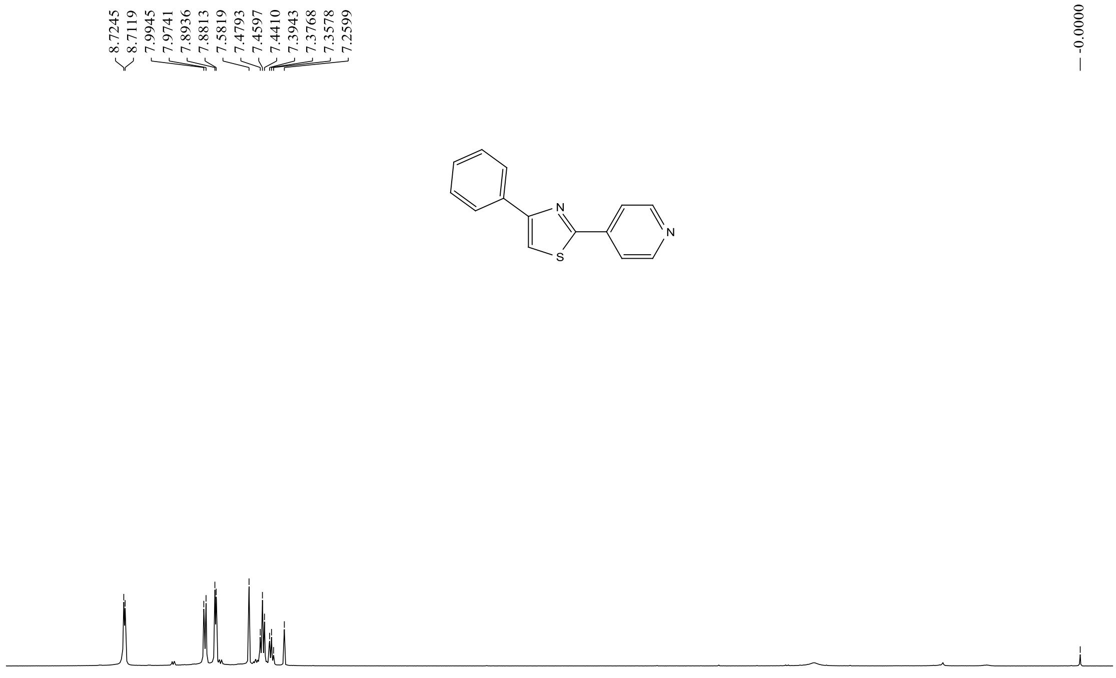

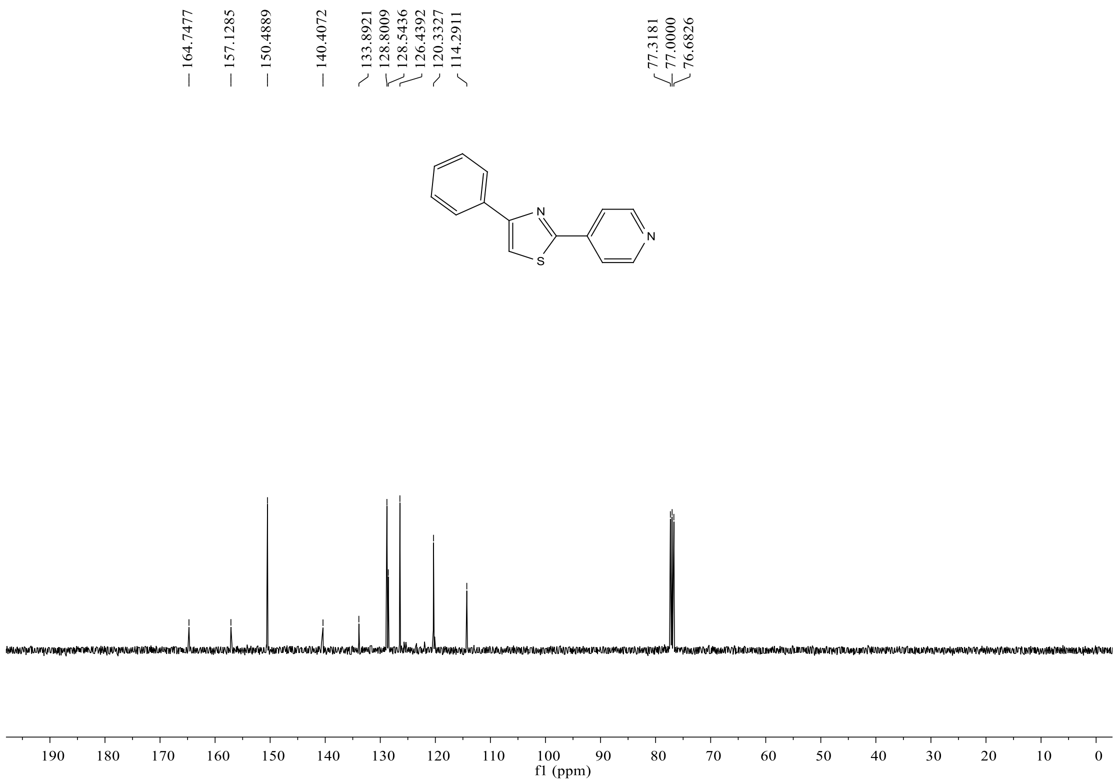

^{1}H and ^{13}C NMR NMR spectra of **7**

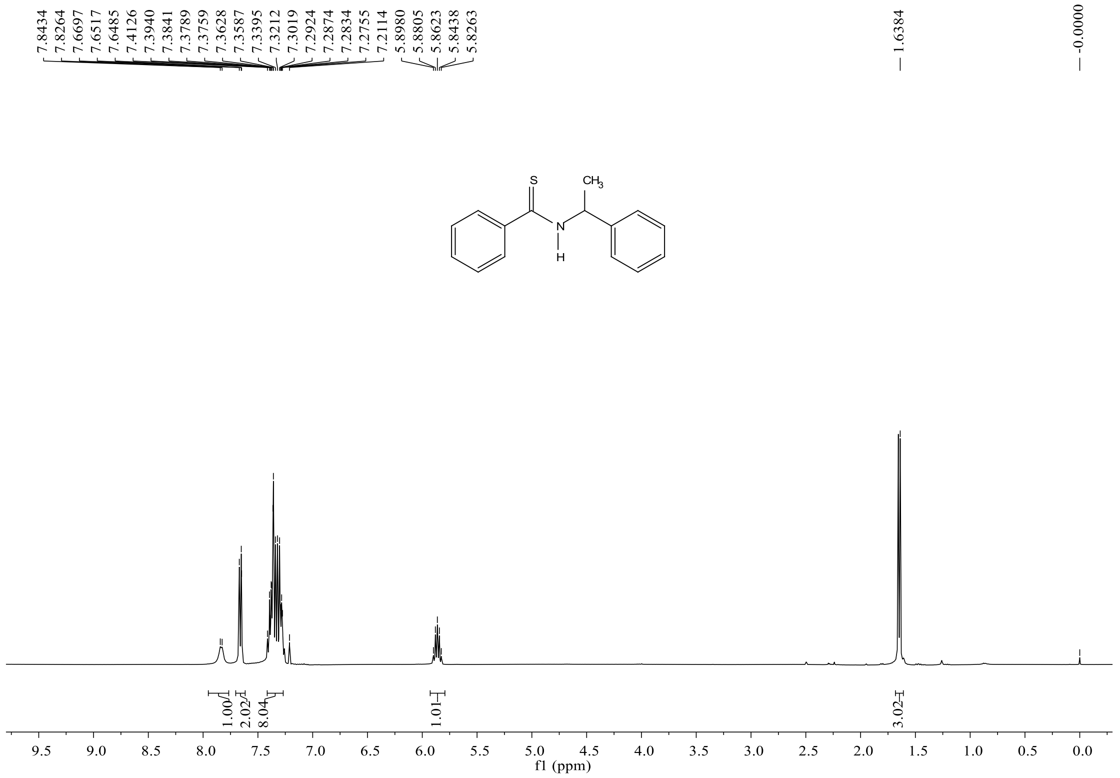

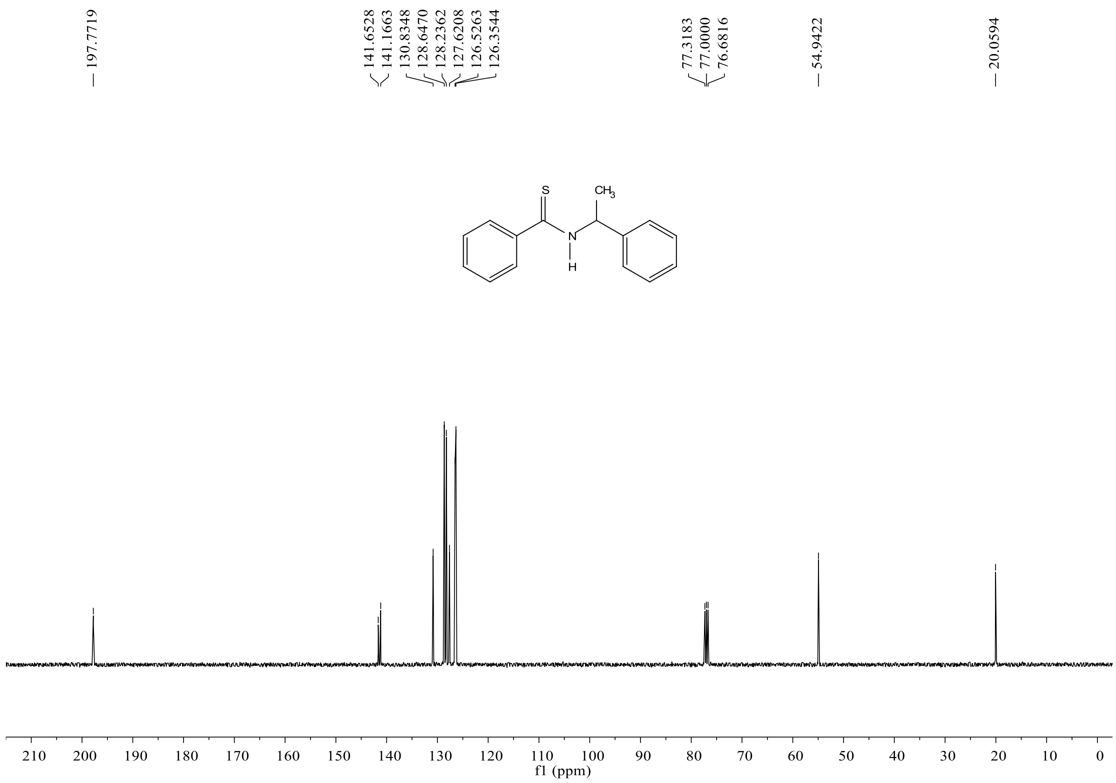

^{1}H and ^{13}C NMR NMR spectra of **8** (CD_3OD-d_4)

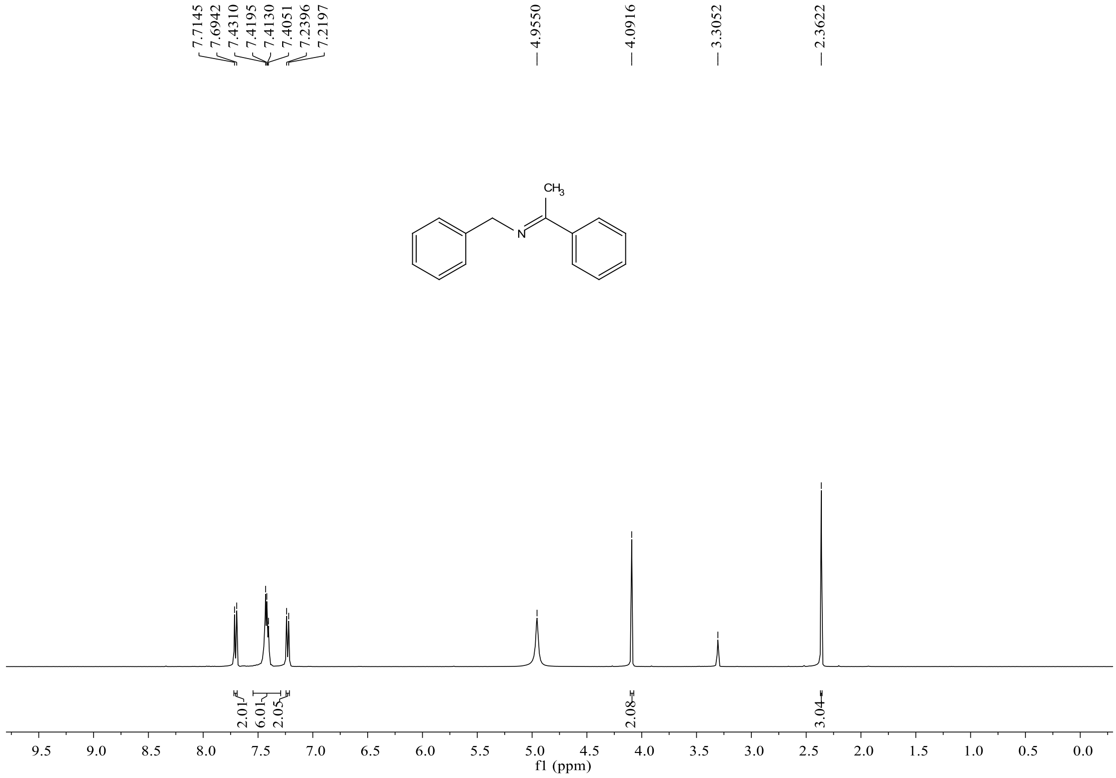

^{1}H and ^{13}C NMR NMR spectra of **9**

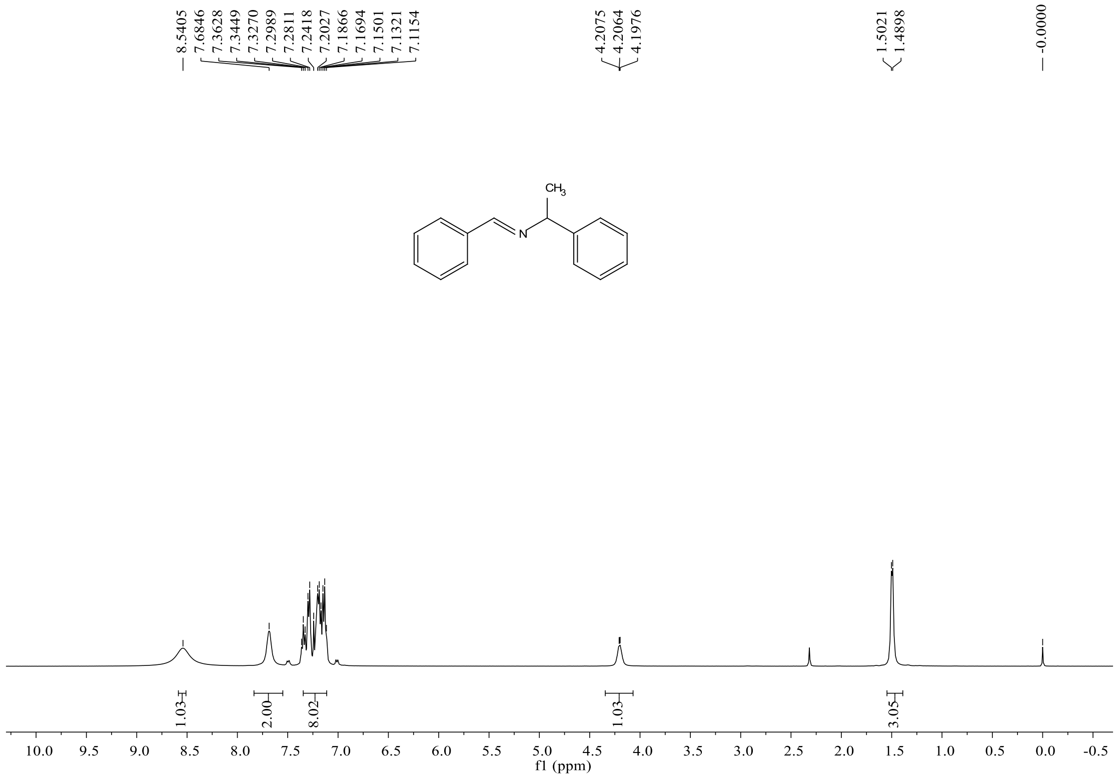

Supplement: RA-010-C9RA09656F-s001 [file RA-010-C9RA09656F-s001.pdf]
